# Comparison of immune checkpoint inhibitors related to pulmonary adverse events: a retrospective analysis of clinical studies and network meta-analysis

**DOI:** 10.1186/s12916-024-03285-3

**Published:** 2024-02-19

**Authors:** Baohui Hong, Bin Du, Rong Chen, Caiyun Zheng, Ruping Ni, Maobai Liu, Jing Yang

**Affiliations:** 1 Department of Pharmacy, Fujian Medical University Union Hospital, Fuzhou, China; 2 Department of Pharmacy, The Second Hospital of Sanming City, Sanming, China; 3 Department of Oncology, Fujian Medical University Union Hospital, Fuzhou, China; 4 Department of Anesthesiology, The Second Hospital of Sanming City, Sanming, China; 5 Fuqing City Hospital Affiliated to Fujian Medical University, Fuzhou, China; 6 Department of Pharmacy, The First Hospital of Nanping City, Nanping, China; 7 The School of Pharmacy, Fujian Medical University, Fuzhou, China

**Keywords:** Tumor, Immune checkpoint inhibitors, Treatment-related adverse events, Pulmonary adverse events

## Abstract

**Background:**

Immune checkpoint inhibitors (ICIs) have transformed tumor treatment. However, the risk of pulmonary adverse events (PAEs) associated with ICI combination therapy is still unclear. We aimed to provide a PAE overview and risk ordering of ICIs used in tumor treatment.

**Methods:**

We searched the databases of PubMed, PsycINFO, Embase, Cochrane Library, CINAHL, Web of Science, Scopus, and clinical trial websites during January 2011–April 2023 to identify phase II and III randomized clinical trials (RCTs) and single-arm clinical trials wherein at least one treatment arm received ICIs (e.g., ICI monotherapy, a combination of two ICIs, or ICIs in combination with conventional cancer therapy). We reported the results of PAEs. Additionally, we compared risks of PAEs between different drug classes using a Bayesian network meta-analysis.

**Results:**

Among 143 RCTs and 24 single-arm trials, the incidence of all-grade and grade 3–4 PAEs were highest with programmed death L1 (PD-L1) plus cytotoxic T-lymphocyte-associated antigen 4 (CTLA-4) and plus chemotherapy and anti-PD1 plus anti-CTLA4, the lowest with targeted therapy drug plus chemotherapy and anti-PD1 plus anti-PDL1. Anti-PD1 plus anti-CTLA4 and plus chemotherapy was the intervention with the highest risk for all-grade and 3–4 grade PAEs, and the intervention with the lowest risk was chemotherapy and anti-PD1 plus anti-PDL1. In terms of all-grade PAEs, chemotherapy was safer than ICI monotherapy. Except for the anti-PD1 plus anti-PDL1 regimen, no significant difference in the risk of grade 3–4 PAEs was detected between dual-ICIs and single-ICIs. Furthermore, the risk of PAEs associated with nivolumab, pembrolizumab, and atezolizumab may be dose dependent.

**Conclusions:**

In the single-drug regimen, anti-PD1 caused the greatest incidence of PAEs. The risk of PAEs was higher with all single-ICIs than with chemotherapy. However, no significant difference in the risk of PAEs was detected between single-ICIs. In the combined regimen, anti-PD1 plus anti-CTLA4 and plus chemotherapy showed the greatest risk of PAEs, but there were no significant differences in risk between dual-ICIs and single-ICIs.

**Supplementary Information:**

The online version contains supplementary material available at 10.1186/s12916-024-03285-3.

## Background

Immune checkpoint inhibitors (ICIs) have dramatically updated tumor therapeutic regimens, including therapies targeting programmed cell death 1 (PD-1), programmed cell death-ligand 1 (PD-L1), and cytotoxic T-lymphocyte antigen 4 (CTLA-4). The inhibition of ICIs reverses T-cell immune tolerance and restores immune system antitumor activity. This phenomenon increases the immune response of the body to the tumor and prevents tumor cells from escaping detection and destruction by T cells [1, 2]. Previous clinical trials have confirmed that ICIs can delay tumor progression and notably improve the overall survival rate of patients with various cancers [3–5]. Recently, indications for ICIs have expanded dramatically [6], and since 2011, the Food and Drug Administration (FDA) has approved the use of multiple ICIs as treatment options for various cancer types. Notably, ICIs have a better safety profile than chemotherapy [7, 8]. However, the widespread use of ICIs for tumors has immune-related adverse events (irAEs), and the incidence of irAEs associated with single-agent ICI therapy is considerable (15–90%) [9, 10]. Notably, a combination of two ICIs or ICIs combined with traditional therapy (such as chemotherapy or targeted therapy) further improves their clinical efficacy [11–13]. However, these complex treatment strategies also come with immune-related issues, and it is inconclusive whether these combination treatments increase the risk of adverse events.

ICI-related pulmonary adverse events (PAEs) are relatively rare irAEs. They mainly occur because of ICI-related pneumonitis, which is a non-infectious inflammatory response localized to the interstitium and alveoli, resulting in variable computed tomography findings and changes in histopathological patterns. ICI-related PAEs are a great concern for clinicians because they can lead to treatment discontinuation and notable mortality rates. However, with timely interventions, PAEs can be controlled, and ICI treatment can be continued after symptoms have subsided [2]. In this study, network meta-analysis (NMA) was performed, combining direct and indirect evidence to compare all ICI-based treatment regimens pairwise and evaluate the risk of PAEs in patients with tumors. Drawing reliable conclusions will help individualize clinical treatment decisions and lower the risk of PAEs in patients.

## Methods

We conducted our study based on registered drafts and followed the guidelines of Preferred Reporting Items for Systematic Reviews and Meta-Analyses (PRISMA) [14]. This study was registered in PROSPERO (registration no.: CRD42023444109).

### Data sources and study selection

We searched the PubMed, Embase, Cochrane Library, PsycINFO, CINAHL, Scopus, and Web of Science databases for relevant articles published from January 2011 to April 2023, with no language restrictions (see Additional File 1: Supplementary Methods for the specific search strategies employed). We identified additional studies by searching the ClinicalTrials.gov website (https://clinicaltrials.gov/). Eligible studies had to be phase II and III randomized clinical trials (RCTs) and single-arm clinical trials. In addition, the following inclusion criteria had to be met: (1) patients: ≥ 18 years of age with a tumor; (2) intervention: at least one treatment arm received ICIs (e.g., ICI monotherapy, a combination of two ICIs, ICIs in combination with chemotherapy or targeted therapy); (3) comparison: blank control group/placebo, chemotherapy, targeted therapy drug or any intervention containing an ICI; and (4) results: PAEs (pneumonitis, interstitial lung disease) determined using Common Terminology Criteria for Adverse Events. We excluded non-randomized studies, cohort studies, and phase I trials.

### Data extraction and quality assessment

Two independent investigators (BH and BD) extracted and assessed the data. BH used standardized data extraction forms to extract relevant information from the included trials, and BD independently reviewed the data. Any disagreements were resolved through discussion. The collected information included basic study characteristics (author, publication year, National Clinical Trial number, trial name, sample size, age, tumor type, line of treatment, disease stage, follow-up time, smoking history, and previous radiation therapy), specific medication regime, and PAEs.

PAEs relating to pulmonary toxicity include pneumonitis and interstitial lung disease, excluding infection-related pulmonary diseases. The outcome measures were treatment-related PAEs. Treatment-related PAEs refer to any PAEs for which treatment cannot be ruled out as a cause. The primary outcome of interest was treatment-related PAEs. The secondary outcomes were fatal PAEs (i.e., Grade 5 PAEs). Treatment-related PAEs were further divided into all-grade and grades 3–4. Fatal PAEs caused death from treatment-related pulmonary toxicities. We only extracted fatal events associated with treatment-related pulmonary toxicities, which were stated in the respective studies.

We independently assessed the quality of the RCTs blinded. BH and BD used the Cochrane Risk of Bias Tool v.2 to assess the risk of bias in each RCT in the NMA.

### Data synthesis and analysis

The incidence rates for all outcomes were calculated using the SPSS software version 26.0. We fit the raw data to a normal distribution after the logit transformation [15]. Further, to determine the corresponding 95% confidence intervals (CIs) for each incidence, we used mixed-effects logistic regression.

Our Bayesian NMA was performed using the Markov Chain Monte Carlo method [16] with R software version 3.5.3, which compared all included interventions. We used a random effect generalized model, running 100,000 inference iterations on each of the four chains. The first 50,000 iterations were discarded as burn-in to obtain the posterior distribution. The Gelman–Rubin method was used to check the convergence of the model by combining density and bundle maps [17]. Evidence relationships were summarized using a network diagram. The PAEs of the different interventions were ranked according to their surface under the cumulative ranking curve (SUCRA) values [18], and the ranking results were visualized using a heat map. The league table compared all treatment regimens. Based on whether the log odds ratio (LOR) passed the zero value, we judged whether there was a significant difference between different interventions [19]. We used bias information criteria to provide a measure of model fit to select a random effects model, a fixed effects model, or an inconsistent model [20]. We then tested the assumption of local consistency via node segmentation [20, 21]. To explore heterogeneity sources, we performed subgroup analyses according to the cancer type and different doses of drugs. To rule out small studies that produced different results, we explored the possibility of publication bias by visually comparing the adjusted funnel plots and performing Egger’s test [22, 23]. Differences were considered statistically significant at *P* < 0.05.

## Results

### Study selection and characteristics

An initial literature search yielded 100,932 records. After excluding duplicate studies, further screening of the titles and abstracts yielded 185 potentially eligible studies. Of these, 167 were included in this meta-analysis (Additional File 1: Figure S1), with 83,181 participants. Additional File 1: Table S1 shows the PRISMA flow diagram, and Additional File 1: Table S2 presents the baseline characteristics of the patients. Additional File 1: Table S3 shows the results of individual studies.

### Risk of bias of the included studies

The quality of the trials was considered acceptable. Many studies had an unclear risk of bias in outcome measurements (37.8%) and missing outcome data (41.3%) (Additional File 1: Figure S2). Moreover, the funnel plot in this NMA showed rough symmetry (Additional File 1: Figure S3). Egger’s test did not reveal significant publication bias in most outcomes, showing *P* < 0.05 only in fatal PAEs and all-grade PAEs of respiratory system cancer.

### PAE incidence

**Fig. 1:**
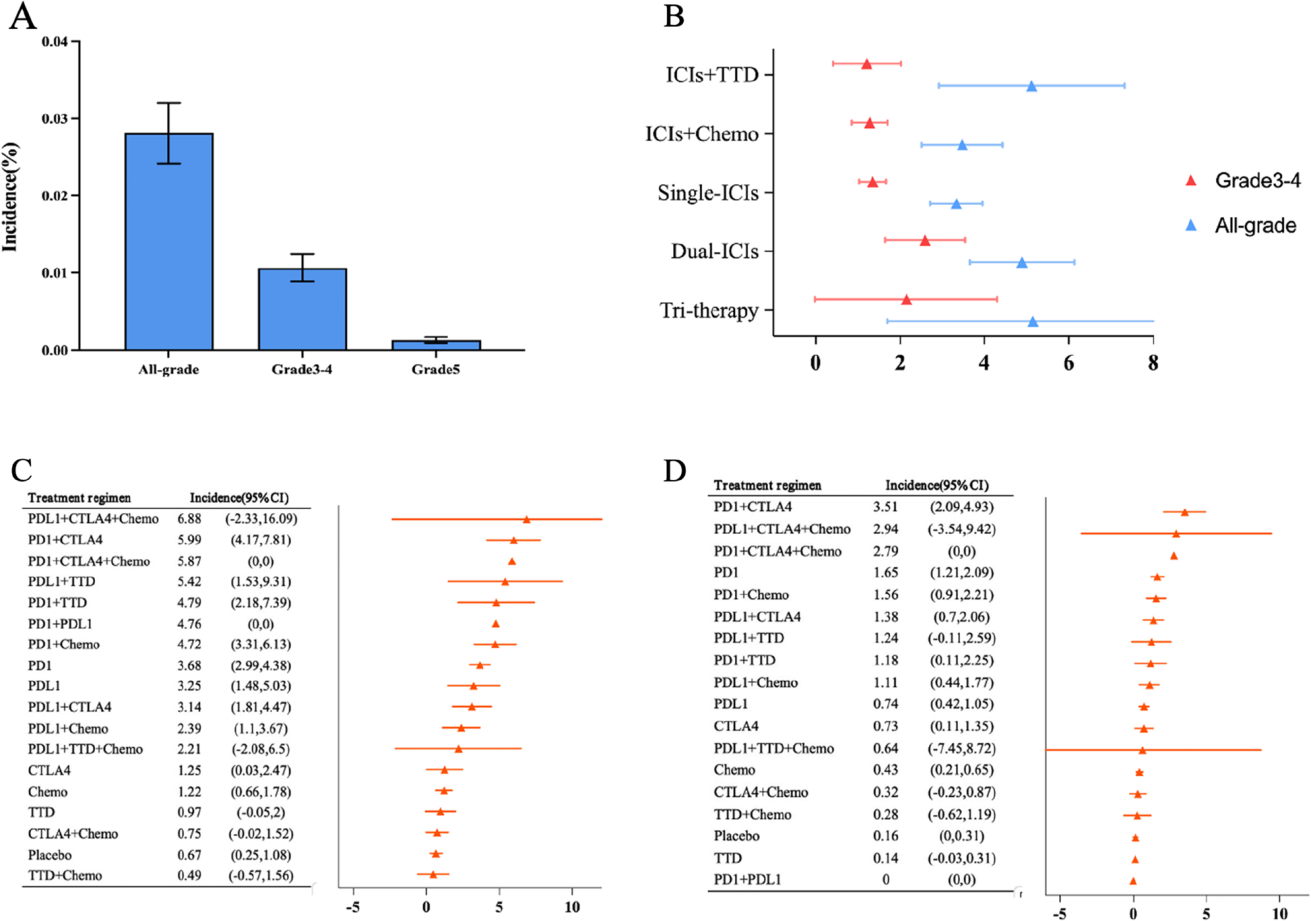
Incidence of pulmonary adverse events (PAEs) in this meta-analysis. **a** Incidence of PAEs by adverse event level. **b** Incidence of PAEs according to the treatment class. **c** Incidence of all-grade PAEs according to the treatment regimen. **d** Incidence of grades 3–4 PAEs according to the treatment regimen

The incidence of grade 3–4 PAEs was 1.06%. The incidence of all-grade PAEs was 2.81%. The incidence of fatal PAEs was 0.13% (Fig. 1a). The incidence of PAEs was highest in patients receiving triple therapy (all-grade: 5.15%; grades 3–4: 2.15%) and lowest in patients receiving single-ICIs (all-grade: 3.33%) and ICI plus targeted therapy drug (grade 3–4:1.21%) (Fig. 1b). From the perspective of the treatment regimen, patients receiving anti-PDL1 plus anti-CTLA4 and plus chemotherapy (all-grade: 6.88%) and anti-PD1 plus anti-CTLA4 (grades 3–4: 3.51%) had the most elevated incidence rate of PAEs; patients receiving targeted therapy drug plus chemotherapy (all-grade: 0.49%) and anti-PD1 plus anti-PDL1 (grades 3–4: 0%) had the lowest incidence of PAEs (Fig. 1c and d).

### NMA

#### Treatment-related PAEs

**Fig. 2:**
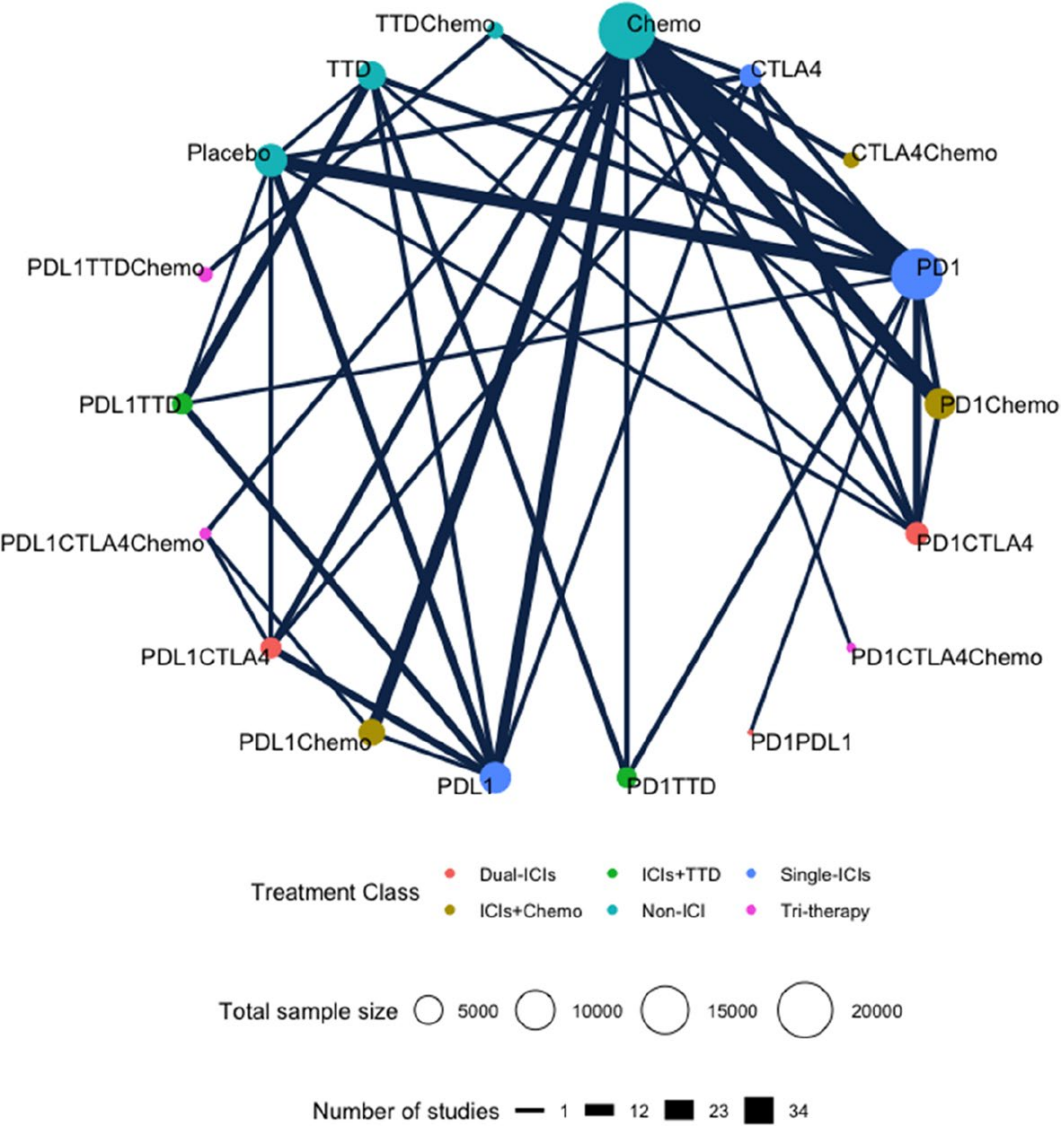
Network plots for PAEs. Different colored nodes represent different types of treatment. The size of the node is positively correlated with the sample size. The width of the lines is positively correlated with the number of studies that directly compared the two treatment regimens

**Fig. 3:**
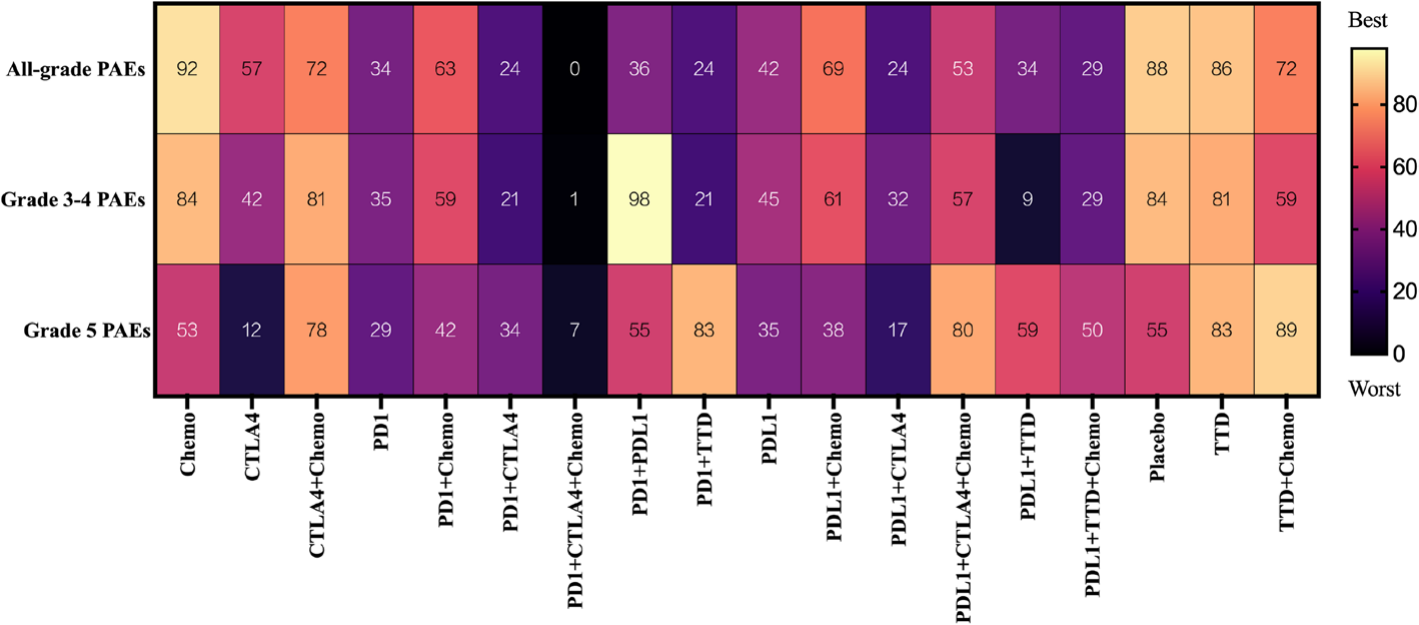
Heat maps of risk for each treatment regimen at different grades of PAEs. In particular, treatment regimens were ranked according to all-grade PAEs, grades 3–4 PAEs, and grade 5 PAEs. The numbers in the squares represent the surface under the cumulative ranking curve (SUCRA) values. These values are measured using a scale from 0 (worst) to 100 (best). The SUCRA value is inversely proportional to the risk of adverse events

Overall, 167 studies were included for the analysis of treatment-related PAEs, comparing 18 interventions (Fig. 2). The SUCRA and RANK rankings of PAEs are shown in Fig. 3. Table 1 shows the corresponding league tables.

**Table 1:**
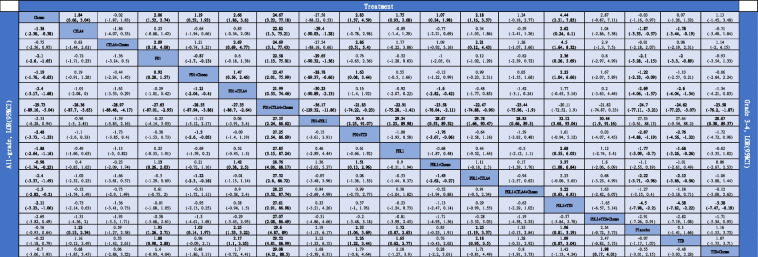
League table for all-grade pulmonary adverse events (PAEs) and grades 3–4 PAEs. Comparisons are based on log odds ratios (LORs) (95% CI). The results on the lower triangle represent LORs (95% CI) for all-grade PAEs, while those on the upper triangle represent LORs (95% CI) for grade 3–4 PAEs. For all-grade PAEs, LORs < 0 favored the column-defining intervention. For grades 3–4 PAEs, LORs < 0 favored the row-defining intervention. Bold cells indicate statistical significance

Chemotherapy was associated with a significantly lower risk of all-grade PAEs than ICI monotherapy. Specifically, it showed significantly lower risk than anti-CTLA4 (LOR: − 1.38, 95% CI: − 2.38 to − 0.38), anti-PD1 (LOR: − 2.10, 95% CI: − 2.60 to − 1.65), and anti-PDL1 (LOR: − 1.88, 95% CI: − 2.64 to − 1.16). The estimated effects were not significant between anti-PD1 and anti-PDL1 (LOR: 0.23, 95% CI: − 0.55–1.01). Except for anti-CTLA4 plus chemotherapy, the risk of PAEs was significantly higher with dual-ICIs than with ICI plus chemotherapy. Anti-PD1 plus chemotherapy (LOR: − 1.21, 95% CI: − 2.04 to − 0.40) and anti-PDL1 plus chemotherapy (LOR: − 1.43, 95% CI: − 2.62 to − 0.27) significantly reduced the risk of PAEs compared with anti-PD1 plus anti-CTLA4 and anti-PDL1 plus anti-CTLA4, respectively. The risk of PAEs was significantly higher with anti-PD1 than with anti-PD1 plus chemotherapy (LOR: 0.92, 95% CI: 0.28–1.57). The SUCRA values showed that anti-PD1 plus anti-CTLA4 and plus chemotherapy (0%) had the greatest risk, and there were significant differences with each treatment regimen. In contrast, chemotherapy (92%) had the least risk and significantly differed from all ICI-based treatment, except for anti-CTLA4 plus chemotherapy, anti-PD1 plus anti-PDL1, and anti-PDL1 plus targeted therapy drug and plus chemotherapy.

Among grade 3–4 treatment-related PAEs, all treatment strategies that included ICIs, except for anti-CTLA4 plus chemotherapy, anti-PD1 plus anti-PDL1, anti-PDL1 plus anti-CTLA4 and plus chemotherapy, and anti-PDL1 plus targeted therapy drug and plus chemotherapy, were associated with a significantly higher risk of PAEs compared with chemotherapy. Except for anti-PD1 plus anti-PDL1, single-ICIs did not have significant differences in the risk of PAEs compared with dual-ICIs. No significant differences were also noted between anti-PD1 plus anti-CTLA4 and anti-PD1 (LOR: 0.6, 95% CI: − 0.18–1.38) and between anti-PDL1 plus anti-CTLA4 and anti-PDL1 (LOR: 0.44, 95% CI: − 0.52–1.44). The SUCRA values showed that anti-PD1 plus anti-CTLA4 and plus chemotherapy (1%) had the greatest risk of PAEs. That differed significantly from the other treatment regimens except for anti-PDL1 plus targeted therapy drug and anti-PDL1 plus targeted therapy drug and plus chemotherapy. Anti-PD1 plus anti-PDL1 (98%) was the regimen with the least risk and differed significantly from the other interventions except for chemotherapy, anti-CTLA4 plus chemotherapy, targeted therapy drug, and placebo.

#### Fatal PAEs

For fatal PAEs, 167 studies compared 18 interventions (Fig. 2). The SUCRA and RANK rankings of fatal PAEs are shown in Fig. 3. Additional File 1: Table S4 shows the corresponding league table. Anti-PD1, anti-PDL1 plus anti-CTLA4, and anti-PD1 plus anti-CTLA4 and plus chemotherapy are associated with a higher risk of fatal PAEs than chemotherapy. Except for anti-CTLA4, the risk of fatal PAEs was significantly higher with single-ICIs and dual-ICIs than with ICI plus targeted therapy drug. The SUCRA ranking indicated that anti-PD1 plus anti-CTLA4 and plus chemotherapy was the deadliest regimen. Except for anti-CTLA4 plus chemotherapy, anti-PD1 plus targeted therapy drug, and anti-PDL1 plus targeted therapy drug, it was not significantly different from the other treatments based on ICI. The least lethal PAEs were related to targeted therapy drug plus chemotherapy. It differed significantly from the other interventions except for anti-CTLA4 plus chemotherapy, anti-PD1 plus anti-PDL1, anti-PD1 plus targeted therapy drug, anti-PDL1 plus anti-CTLA4 and plus chemotherapy, anti-PDL1 plus targeted therapy drug, anti-PDL1 plus targeted therapy drug and plus chemotherapy, and targeted therapy drug.

### NMA of PAEs by cancer type

Subgroup analysis of PAEs was performed according to the tumor location, such as the respiratory, genitourinary, skin, head and neck, and digestive systems. Additional File 1: Figure S4a–e show the network plots, and Additional File 1: Figure S5 shows the SUCRA values. Additional File 1: Tables S5 and S6 are league tables. Among patients with respiratory tumors, those who received anti-PD1 plus anti-CTLA4 and plus chemotherapy showed the greatest risk of all-grade PAEs, which was significantly different from the other interventions. The regimen with the greatest risk of grade 3–4 PAEs was anti-PD1 plus anti-CTLA4 and plus chemotherapy, which differed significantly from the other interventions except for targeted therapy drug and anti-PDL1 plus targeted therapy drug. Patients with urogenital tumors treated with anti-PDL1 plus targeted therapy drug had the greatest risk of developing all-grade PAEs, and it differed significantly from the risks associated with anti-PDL1 plus chemotherapy, anti-PD1 plus chemotherapy, targeted therapy drug, and placebo. The greatest risk of grade 3–4 PAEs in patients with urogenital cancer was related to anti-PDL1 plus targeted therapy drug, which had significant differences with the other interventions except for anti-PD1, anti-PD1 plus targeted therapy drug, and anti-PDL1 plus anti-CTLA4. The greatest risk of all-grade PAEs in patients with skin cancer was related to anti-PDL1 plus targeted therapy drug, which differed significantly from the other interventions except for targeted therapy drug and anti-PD1 plus anti-CTLA4. The greatest risk of grade 3–4 PAEs in patients with skin cancer was from anti-PDL1 plus targeted therapy drug, which differed significantly from the other interventions except for anti-CTLA4 plus chemotherapy and anti-PD1 plus anti-CTLA4. The greatest risk of all-grade PAEs in patients with head and neck cancer was linked to anti-PD1 plus targeted therapy drug, which had significant differences with the other interventions except for anti-PDL1 plus chemotherapy. The greatest risk of grade 3–4 PAEs in patients with head and neck cancer was linked to anti-PD1 plus targeted therapy drug, which had significant differences with the other interventions except for anti-PD1 plus chemotherapy. Furthermore, the greatest risk of all-grade PAEs in patients with digestive system tumors was linked to anti-PDL1 plus anti-CTLA4, which was significantly different from the other interventions except for anti-PD1-based interventions. Finally, the greatest risk of grade 3–4 PAEs in patients with digestive system tumors was associated with anti-PD1 plus anti-CTLA4, which differed significantly from the other interventions except for anti-PDL1 plus anti-CTLA4, and anti-PD1 plus targeted therapy drug.

### ICI-induced PAEs at different doses

We also performed subgroup analyses based on ICI doses (Additional File 1: Figure S6). Compared with placebo, nivolumab 360 mg q3w showed a significant difference in the risk of PAEs in all-grade, whereas nivolumab 240 mg q2w did not. When the intervention group was atezolizumab plus targeted therapy drug and the control group was targeted therapy drug, the 840 mg q2w atezolizumab regimen was significantly different in the risk of all-grade PAEs compared with targeted therapy drug. There was no significant difference between the 1200 mg q3w atezolizumab treatment and targeted therapy drug. Compared with chemotherapy, the pembrolizumab 2 mg/kg q3w regimen had a statistically significant difference in the risk of grade 3–4 PAEs, and the treatment with pembrolizumab 10 mg/kg q3w treatment did not differ significantly from the control group.

### Additional analyses

According to the DIC values, the stochastic consistency model is the preferred model with a good complexity trade-off between model fitting and model (Additional File 1: Table S7). Bayesian network analysis revealed that the shrinkage factors of Brooks–Gelman–Rubin diagnostic graphs were < 1.2 (Additional File 1: Table S7), indicating that the research model had good convergence. Values with I^2^ > 50% were not observed; consequently, significant heterogeneity was not observed in PAEs. The node splitting method demonstrated no significant inconsistencies in the results (Additional File 1: Table S8). Therefore, the overall results were considered relatively robust.

## Discussion

To the best of our knowledge, this is the most comprehensive and largest NMA that has investigated the incidence of PAEs in patients with ICI-treated cancer. The incidence rates of PAEs and fatal PAEs were 2.81% and 0.13%, respectively, which is consistent with those reported in previous studies [24, 25]. Simultaneously, we found differences in the safety rankings of treatment regimens for different cancers regarding PAEs. These findings can help clinicians increase the awareness regarding risk of PAEs when using different drugs to treat specific tumors.

### Treatment-related PAEs

For ICI monotherapy, anti-PD1 had the highest incidence of all-grade PAEs. However, there was no significant difference between anti-PD1 and anti-PDL1 in the incidence of all-grade PAEs. In addition, the incidence of ICI monotherapy varied across studies owing to the different dose regimens. Here, the risk of all-grade PAEs was dose-dependent for nivolumab, pembrolizumab, and atezolizumab, whereas no significant difference in PAEs was observed between dose groups for the other ICIs. By contrast, Wu et al. hypothesized that the incidence and intensity of PAEs induced by anti-PD1 are independent of drug dose [26]. However, this difference may be due to the inclusion of more recent studies in our analysis and consideration of treatment-related PAE differentiation. In addition, our control group was administered the same drug. Wu et al. only included studies published until 2016, and the types of drugs in the control group were not consistent. Therefore, it is not surprising that the incidence of PAEs varied with regimens. Higher doses of nivolumab and a low dose and high frequency of atezolizumab administration in combination with targeted therapy may also be a factor affecting the risk of all-grade PAEs. Therefore, to prevent further aggravation of PAEs in patients using anti-PD1/anti-PDL1 with different usage and dosage, clinicians must focus on minor changes in these patients, including timely diagnosis and intervention.

Recently, the FDA and EMA have approved several ICIs in combination with chemotherapy or targeted therapies. In addition, some cancers already use combination therapy as the standard of care. For combination treatment regimens, we have found the following points. First, anti-PD1 plus anti-CTLA4 and plus chemotherapy had the greatest risk of PAEs, which differed significantly from the other regimens. This may be due to the increased risk of PAEs associated with drug combinations with different mechanisms of action. Association of anti-CTLA4 and anti-PD1 enhances T cell activation and proliferation. This increases the production of proinflammatory cytokines [27]. Second, the incidence of PAEs was much higher with anti-PD1 plus anti-CTLA4 than with ICI monotherapy, but there was no significant difference in the risk of PAEs. By contrast, Nishino et al. suggested that the incidence of developing pneumonitis is higher with combination therapy than with ICI monotherapy. This may be because their study was limited to three ICI molecules, the anti-PD1 nivolumab, pembrolizumab, and the anti-CTLA4 agent ipilimumab. However, we also included atezolizumab, avelumab, durvalumab, tremelimumab, camrelizumab, cemiplimab, sintilimab, and tislelizumab. Third, the risk of all-grade PAEs was significantly lower with ICI plus chemotherapy than with dual-ICIs, and the risk of all-grade PAEs was significantly lower with anti-PD1 plus chemotherapy than with anti-PD1 monotherapy. These observations are in agreement with those of Chen et al. [28]. A possible reason is that the traditional chemotherapy regimen primarily consists of cytotoxic drugs, which induce immunosuppression and lead to decreased immune function, thereby reducing the risk of immune-related adverse reactions [29, 30]. Another possible factor is that the combination of pre-treated glucocorticoids in the chemotherapy regimen reduces the risk of PAEs. Glucocorticoids can not only suppress the immune system [31, 32] but also have certain therapeutic effects on some lung diseases, including ICI-related pneumonitis [33, 34]. However, the mechanism of how cytotoxic drugs and corticosteroids combined with ICI regulate the immune system remains unclear. More prospective studies of the mechanisms described above are essential to maximize anti-cancer benefits while minimizing the risks of PAEs. Moreover, we included the latest drug therapy combination, anti-PD1 plus anti-PDL1. Interestingly the incidence of all-grade PAEs is not low, but the risk of grade 3–4 PAEs is low. Although the underlying mechanism is still unclear, our results provide clinicians with the latest reference for PAEs of drug combinations.

Our study has several strengths as we rigorously and comprehensively searched the literature for patients with ICI-treated cancer. We included cohorts of patients worldwide, making our findings more generalizable. Additionally, several studies with relatively large sample sizes were included, which increased the statistical power of our meta-analysis. However, our study also had a few limitations, which may be methodological flaws. First, the studies we included were all RCTs; therefore, the baseline conditions of the patients were highly selected and may not reflect the real-world situation. Second, PAEs include a variety of pulmonary toxicity outcomes, and the measurement criteria of different outcomes and the number of trials by various authors lacked some transparency in each study. Finally, we did not investigate the performance of different Bayesian meta-analytic methods in rare events [19].

## Conclusions

In the single-drug regimen, anti-PD1 showed the greatest incidence of PAEs, and the risk of PAEs from single-ICIs was higher than that of chemotherapy, whereas no significant difference was observed in the PAE risk between single-ICIs. In the combined regimen, anti-PD1 plus anti-CTLA4 and plus chemotherapy had the greatest risk of PAEs, but there was no significant difference in the risk between dual-ICIs and single-ICIs. Therefore, we recommend a more individualized approach to evaluate the risk of managing PAEs in such patients.

### Supplementary Information

**Additional file 1: Supplementary Methods.** Search Terms. **Table S1.** PRISMA NMA Checklist. **Table S2.** Study and Patient Characteristics. **Table S3.** Results of individual studies. **Table S4.** Fatal Pulmonary Adverse Events Outcome: League Table. **Table S5.** All-grade Treatment-related Pulmonary Adverse Events in Tumors of Different Systems Outcome: League Table. **Table S6.** Grade 3–4 Treatment-related Pulmonary Adverse Events in Tumors of Different Systems Outcome: League Table.** Table S7.** Model selection of All Outcomes. **Table S8.** Inconsistency Test. **Figure S1.** Flowchart of Study Selection. **Figure S2.** Risk of Bias Assessment for Studies. **Figure S3.** Comparison-adjusted funnel plots. **Figure S4.** Network plots. **Figure S5.** The distribution of SUCRA values stratified by cancer types. **Figure S6.** Subgroup analysis by dose of drug.

## Data Availability

All data generated or analyzed during this study are included in this published article and its supplementary information files. Further inquiries can be directed to the corresponding author.

## Abbreviations

CIs: Confidence intervals
CTLA-4: Cytotoxic T-lymphocyte-associated protein-4
FDA: Food and Drug Administration
ICIs: Immune checkpoint inhibitors
irAEs: Immune-related adverse events
LOR: Log odds ratio
NMA: Network meta-analysis
PAEs: Pulmonary adverse events
PD-1: Programmed cell death protein 1
PD-L1: Programmed cell death-ligand 1
PRISMA: Preferred Reporting Items for Systematic Reviews and Meta-Analyses
RCTs: Randomized clinical trials
SUCRA: Surface under the cumulative ranking curve
TTD: Targeted therapy drug

## References

[1] Li X, Shao C, Shi Y, Han W (2018). Lessons learned from the blockade of immune checkpoints in cancer immunotherapy. J Hematol Oncol.

[2] Thompson JA, Schneider BJ, Brahmer J, Andrews S, Armand P, Bhatia S, Budde LE, Costa L, Davies M, Dunnington D (2020). NCCN guidelines insights: management of immunotherapy-related toxicities, version 1.2020. J Natl Compr Canc Netw.

[3] Hodi FS, O'Day SJ, McDermott DF, Weber RW, Sosman JA, Haanen JB, Gonzalez R, Robert C, Schadendorf D, Hassel JC (2010). Improved survival with ipilimumab in patients with metastatic melanoma. N Engl J Med.

[4] Gadgeel SM, Stevenson JP, Langer CJ, Gandhi L, Borghaei H, Patnaik A, Villaruz LC, Gubens M, Hauke R, Yang JC (2018). Pembrolizumab and platinum-based chemotherapy as first-line therapy for advanced non-small-cell lung cancer: phase 1 cohorts from the KEYNOTE-021 study. Lung Cancer.

[5] Nayak L, Iwamoto FM, LaCasce A, Mukundan S, Roemer MGM, Chapuy B, Armand P, Rodig SJ, Shipp MA (2017). PD-1 blockade with nivolumab in relapsed/refractory primary central nervous system and testicular lymphoma. Blood.

[6] Seidel JA, Otsuka A, Kabashima K (2018). Anti-PD-1 and anti-CTLA-4 therapies in cancer: mechanisms of action, efficacy, and limitations. Front Oncol.

[7] Robert C, Long GV, Brady B, Dutriaux C, Maio M, Mortier L, Hassel JC, Rutkowski P, McNeil C, Kalinka-Warzocha E (2015). Nivolumab in previously untreated melanoma without BRAF mutation. N Engl J Med.

[8] Reck M, Rodríguez-Abreu D, Robinson AG, Hui R, Csőszi T, Fülöp A, Gottfried M, Peled N, Tafreshi A, Cuffe S (2016). Pembrolizumab versus chemotherapy for PD-L1-positive non-small-cell lung cancer. N Engl J Med.

[9] Puzanov I, Diab A, Abdallah K, Bingham CO, Brogdon C, Dadu R, Hamad L, Kim S, Lacouture ME, LeBoeuf NR (2017). Managing toxicities associated with immune checkpoint inhibitors: consensus recommendations from the Society for Immunotherapy of Cancer (SITC) Toxicity Management Working Group. J Immunother Cancer.

[10] Kumar V, Chaudhary N, Garg M, Floudas CS, Soni P, Chandra AB (2017). Current diagnosis and management of Immune Related Adverse Events (irAEs) induced by immune checkpoint inhibitor therapy. Front Pharmacol.

[11] Gandhi L, Rodríguez-Abreu D, Gadgeel S, Esteban E, Felip E, De Angelis F, Domine M, Clingan P, Hochmair MJ, Powell SF (2018). Pembrolizumab plus chemotherapy in metastatic non-small-cell lung cancer. N Engl J Med.

[12] Rini BI, Plimack ER, Stus V, Gafanov R, Hawkins R, Nosov D, Pouliot F, Alekseev B, Soulières D, Melichar B (2019). Pembrolizumab plus axitinib versus sunitinib for advanced renal-cell carcinoma. N Engl J Med.

[13] Makker V, Rasco D, Vogelzang NJ, Brose MS, Cohn AL, Mier J, Di Simone C, Hyman DM, Stepan DE, Dutcus CE (2019). Lenvatinib plus pembrolizumab in patients with advanced endometrial cancer: an interim analysis of a multicentre, open-label, single-arm, phase 2 trial. Lancet Oncol.

[14] Moher D, Liberati A, Tetzlaff J, Altman DG (2009). Preferred reporting items for systematic reviews and meta-analyses: the PRISMA statement. PLoS Med.

[15] Barendregt JJ, Doi SA, Lee YY, Norman RE, Vos T (2013). Meta-analysis of prevalence. J Epidemiol Community Health.

[16] Gelman A, Rubin DB (1996). Markov chain Monte Carlo methods in biostatistics. Stat Methods Med Res.

[17] Brooks SP, Gelman A (1998). General methods for monitoring convergence of iterative simulations. J Comput Graph Stat.

[18] Salanti G, Ades AE, Ioannidis JP (2011). Graphical methods and numerical summaries for presenting results from multiple-treatment meta-analysis: an overview and tutorial. J Clin Epidemiol.

[19] Hong H, Wang C, Rosner GL (2021). Meta-analysis of rare adverse events in randomized clinical trials: Bayesian and frequentist methods. Clin Trials.

[20] Lu G, Ades AE (2006). Assessing evidence inconsistency in mixed treatment comparisons. J Am Stat Assoc.

[21] Dias S, Welton NJ, Caldwell DM, Ades AE (2010). Checking consistency in mixed treatment comparison meta-analysis. Stat Med.

[22] Salanti G, Del Giovane C, Chaimani A, Caldwell DM, Higgins JP (2014). Evaluating the quality of evidence from a network meta-analysis. PLoS ONE.

[23] Sterne JA, Sutton AJ, Ioannidis JP, Terrin N, Jones DR, Lau J, Carpenter J, Rücker G, Harbord RM, Schmid CH (2011). Recommendations for examining and interpreting funnel plot asymmetry in meta-analyses of randomised controlled trials. BMJ.

[24] Wang Y, Zhou S, Yang F, Qi X, Wang X, Guan X, Shen C, Duma N, Vera Aguilera J, Chintakuntlawar A (2019). Treatment-related adverse events of PD-1 and PD-L1 inhibitors in clinical trials: a systematic review and meta-analysis. JAMA Oncol.

[25] Yu X, Zhang X, Yao T, Zhang Y, Zhang Y (2021). Fatal adverse events associated with immune checkpoint inhibitors in non-small cell lung cancer: a systematic review and meta-analysis. Front Med (Lausanne).

[26] Wu J, Hong D, Zhang X, Lu X, Miao J (2017). PD-1 inhibitors increase the incidence and risk of pneumonitis in cancer patients in a dose-independent manner: a meta-analysis. Sci Rep.

[27] Ramos-Casals M, Brahmer JR, Callahan MK, Flores-Chávez A, Keegan N, Khamashta MA, Lambotte O, Mariette X, Prat A, Suárez-Almazor ME (2020). Immune-related adverse events of checkpoint inhibitors. Nat Rev Dis Primers.

[28] Chen X, Zhang Z, Hou X, Zhang Y, Zhou T, Liu J, Lin Z, Fang W, Yang Y, Ma Y, et al. Immune-related pneumonitis associated with immune checkpoint inhibitors in lung cancer: a network meta-analysis. J Immunother Cancer. 2020;8(2):e001170. 10.1136/jitc-2020-001170 PMC7462235 32863271

[29] Steele TA (2002). chemotherapy-induced immunosuppression and reconstitution of immune function. Leuk Res.

[30] Krisl JC, Doan VP (2017). Chemotherapy and transplantation: the role of immunosuppression in malignancy and a review of antineoplastic agents in solid organ transplant recipients. Am J Transplant.

[31] Oppong E, Cato AC (2015). Effects of glucocorticoids in the immune system. Adv Exp Med Biol.

[32] Löwenberg M, Tuynman J, Bilderbeek J, Gaber T, Buttgereit F, van Deventer S, Peppelenbosch M, Hommes D (2005). Rapid immunosuppressive effects of glucocorticoids mediated through Lck and Fyn. Blood.

[33] Haanen J, Carbonnel F, Robert C, Kerr KM, Peters S, Larkin J, Jordan K (2017). Management of toxicities from immunotherapy: ESMO clinical practice guidelines for diagnosis, treatment and follow-up. Ann Oncol.

[34] Thompson JA (2018). New NCCN guidelines: recognition and management of immunotherapy-related toxicity. J Natl Compr Canc Netw.

