# Supplementary material for: Comparison of immune checkpoint inhibitors related to pulmonary adverse events: a retrospective analysis of clinical studies and network meta-analysis
Source: BMC Med. 2024 Feb 19;22:75. doi: 10.1186/s12916-024-03285-3 (PMC10875746; doi:10.1186/s12916-024-03285-3)
Supplement: Supplementary file 1 — Additional file 1: Supplementary Methods. Search Terms. Table S1. PRISMA NMA Checklist. Table S2. Study and Patient Characteristics. Table S3. Results of individual studies. Table S4. Fatal Pulmonary Adverse Events Outcome: League Table. Table S5. All-grade Treatment-related Pulmonary Adverse Events in Tumors of Different Systems Outcome: League Table. Table S6. Grade 3–4 Treatment-related Pulmonary Adverse Events in Tumors of Different Systems Outcome: League Table. Table S7. Model selection of All Outcomes. Table S8. Inconsistency Test. Figure S1. Flowchart of Study Selection. Figure S2. Risk of Bias Assessment for Studies. Figure S3. Comparison-adjusted funnel plots. Figure S4. Network plots. Figure S5. The distribution of SUCRA values stratified by cancer types. Figure S6. Subgroup analysis by dose of drug. [file 12916_2024_3285_MOESM1_ESM.pdf]

Supplemental materials

Supplementary Methods. Search Terms
Table S1. PRISMA NMA Checklist
Table S2. Study and Patient Characteristics
Table S3. Results of individual studies
Table S4. Fatal Pulmonary Adverse Events Outcome: League Table
Table S5. All-grade Treatment-related Pulmonary Adverse Events in Tumors of Different Systems Outcome: League Table
Table S6. Grade 3-4 Treatment-related Pulmonary Adverse Events in Tumors of Different Systems Outcome: League Table
Table S7. Model selection of All Outcomes
Table S8. Inconsistency Test
Figure S1. Flowchart of Study Selection
Figure S2. Risk of Bias Assessment for Studies
Figure S3. Comparison-adjusted funnel plots
Figure S4. Network plots
Figure S5. The distribution of SUCRA values stratified by cancer types
Figure S6. Subgroup analysis by dose of drug

This supplementary material has been provided by the authors to give readers additional information about their work.

Supplementary Methods. Search Terms

#1<br>"Neoplasms"[mh] OR Neoplasms[tiab] OR Neoplasm[tiab] OR Cancer[tiab] OR Cancers[tiab] OR Tumors [tiab] OR Tumor[tiab] OR Malignancy[tiab] OR Malignancies[tiab]
#2<br>Programmed death ligand 1[tiab] OR PD-L1[tiab] OR Programmed death 1[tiab] OR PD-1[tiab] OR Cytotoxic T-lymphocyte antigen 4[tiab] OR CTLA-4[tiab] OR Immune Checkpoint Inhibitor[tiab] OR immune therapy[tiab] OR immunotherapy[tiab]<br>OR<br>Nivolumab[tiab] OR MDX-1106[tiab] OR ONO-4538[tiab] OR BMS-936558[tiab] OR Opdivo [tiab] OR<br>Pembrolizumab[tiab] OR lambrolizumab[tiab] OR Keytruda[tiab] OR MK-3475[tiab]<br>OR<br>Cemiplimab[tiab] OR Toripalimab[tiab] OR JS001[tiab]OR Sintilimab[tiab]<br>OR<br>Durvalumab [tiab]<br>OR<br>Atezolizumab[tiab] OR MPDL3280A[tiab] OR tecentriq[tiab] OR RG7446[tiab] OR RG-7446[tiab] OR<br>Avelumab[tiab] OR MSB0010718C[tiab]<br>OR<br>Ipilimumab[tiab] OR Anti CTLA 4 MAb Ipilimumab[tiab] OR Anti-CTLA-4 MAb Ipilimumab[tiab] OR Ipilimumab, Anti-CTLA-4 MAb[tiab] OR Yervoy[tiab] OR MDX010[tiab] OR MDX 010[tiab] OR MDX-010 M[tiab] OR MDX CTLA 4[tiab]<br>OR<br>Tremelimumab[tiab] OR ticilimumab[tiab] OR CP 675[Title/Abstract] OR CP675 cpd[tiab] OR CP-675[tiab] OR CP-675,206[tiab] OR CP-675206[tiab] OR CP675206[tiab] OR CP 675206[tiab]<br>OR<br>Camrelizumab[tiab]OR SHR-1210[tiab]OR SHR 1210[tiab]
#3<br>(randomized controlled trial[pt] OR controlled clinical trial[pt] OR randomized[tiab] OR placebo[tiab] OR clinical trials as topic[mesh:noexp] OR randomly[tiab] OR trial[ti]) NOT (animals[mh] NOT (humans[mh] AND animals[mh]))
#4<br>"2011/01/01"[Date - Publication] : "2023/04/30"[Date - Publication]
#5<br>#1AND#2AND#3AND#4

Table S1 PRISMA NMA Checklist

Section/Topic	Item	Checklist Item	Reported on Page
**TITLE**			
Title	1	Identify the report as a systematic review *incorporating a network meta-analysis (or related form of meta-analysis).*	1
**ABSTRACT**			
Structured summary	2	Provide a structured summary including, as applicable:<br>**Background:** main objectives<br>**Methods:** data sources; study eligibility criteria, participants, and interventions; study appraisal; and *synthesis methods, such as network meta-analysis.*<br>**Results:** number of studies and participants identified; summary estimates with corresponding confidence/credible intervals; *treatment rankings may also be discussed. Authors may choose to summarize pairwise comparisons against a chosen treatment included in their analyses for brevity.*<br>**Discussion/Conclusions:** limitations; conclusions and implications of findings.<br>**Other:** primary source of funding; systematic review registration number with registry name.	2-3
**INTRODUCTION**			
Rationale	3	Describe the rationale for the review in the context of what is already known*, including mention of why a network meta-analysis has been conducted.*_	3
Objectives	4	Provide an explicit statement of questions being addressed, with reference to participants, interventions, comparisons, outcomes, and study design (PICOS).	3
**METHODS**			
Protocol and registration	5	Indicate whether a review protocol exists and if and where it can be accessed (e.g., Web address); and, if available, provide registration information, including registration number.	4
Eligibility criteria	6	Specify study characteristics (e.g., PICOS, length of follow-up) and report characteristics (e.g., years considered, language, publication status) used as criteria for eligibility, giving rationale. *Clearly describe eligible treatments included in the treatment network, and note whether any have been clustered or merged into the same node (with justification).*_	4-5
Information sources	7	Describe all information sources (e.g., databases with dates of coverage, contact with study authors to identify additional studies) in the search and date last searched.	5
Search	8	Present full electronic search strategy for at least one	Supplemnt

Section/Topic	Item	Checklist Item	Reported on Page
		database, including any limits used, such that it could be repeated.	eMethods
Study selection	9	State the process for selecting studies (i.e., screening, eligibility, included in systematic review, and, if applicable, included in the meta-analysis).	Figure S1
Data collection process	10	Describe method of data extraction from reports (e.g., piloted forms, independently, in duplicate) and any processes for obtaining and confirming data from investigators.	5
Data items	11	List and define all variables for which data were sought (e.g., PICOS, funding sources) and any assumptions and simplifications made.	5
**Geometry of the network**	**S1**	Describe methods used to explore the geometry of the treatment network under study and potential biases related to it. This should include how the evidence base has been graphically summarized for presentation, and what characteristics were compiled and used to describe the evidence base to readers.	6
Risk of bias within individual studies	12	Describe methods used for assessing risk of bias of individual studies (including specification of whether this was done at the study or outcome level), and how this information is to be used in any data synthesis.	6
Summary measures	13	State the principal summary measures (e.g., risk ratio, difference in means). *Also describe the use of additional summary measures assessed, such as treatment rankings and surface under the cumulative ranking curve (SUCRA) values, as well as modified approaches used to present summary findings from meta-analyses.*	6
Planned methods of analysis	14	Describe the methods of handling data and combining results of studies for each network meta-analysis. This should include, but not be limited to:<br>• *Handling of multi-arm trials;*<br>• *Selection of variance structure;*<br>• *Selection of prior distributions in Bayesian analyses; and*<br>• *Assessment of model fit.*	6
**Assessment of Inconsistency**	**S2**	Describe the statistical methods used to evaluate the agreement of direct and indirect evidence in the treatment network(s) studied. Describe efforts taken to address its presence when found.	6
Risk of bias across studies	15	Specify any assessment of risk of bias that may affect the cumulative evidence (e.g., publication bias, selective reporting within studies).	6
Additional	16	Describe methods of additional analyses if done, indicating	6

Section/Topic	Item	Checklist Item	Reported on Page
analyses		which were pre-specified. This may include, but not be limited to, the following:<br>• Sensitivity or subgroup analyses;<br>• Meta-regression analyses;<br>• *Alternative formulations of the treatment network; and*<br>• *Use of alternative prior distributions for Bayesian analyses (if applicable).*	
**RESULTS**			
Study selection	17	Give numbers of studies screened, assessed for eligibility, and included in the review, with reasons for exclusions at each stage, ideally with a flow diagram.	7,Figure S1
**Presentation of network structure**	**S3**	Provide a network graph of the included studies to enable visualization of the geometry of the treatment network.	Figure 2, Figure S4
**Summary of network geometry**	**S4**	Provide a brief overview of characteristics of the treatment network. This may include commentary on the abundance of trials and randomized patients for the different interventions and pairwise comparisons in the network, gaps of evidence in the treatment network, and potential biases reflected by the network structure.	8-10
Study characteristics	18	For each study, present characteristics for which data were extracted (e.g., study size, PICOS, follow-up period) and provide the citations.	7,Table S2
Risk of bias within studies	19	Present data on risk of bias of each study and, if available, any outcome level assessment.	7,Figure S2
Results of individual studies	20	For all outcomes considered (benefits or harms), present, for each study: 1) simple summary data for each intervention group, and 2) effect estimates and confidence intervals. *Modified approaches may be needed to deal with information from larger networks.*	Table S3
Synthesis of results	21	Present results of each meta-analysis done, including confidence/credible intervals. *In larger networks, authors may focus on comparisons versus a particular comparator (e.g. placebo or standard care), with full findings presented in an appendix. League tables and forest plots may be considered to summarize pairwise comparisons.* If additional summary measures were explored (such as treatment rankings), these should also be presented.	8-11<br>Table 1,<br>Table S4,<br>Table S5,<br>Table S6<br>Figure S5
**Exploration for inconsistency**	**S5**	Describe results from investigations of inconsistency. This may include such information as measures of model fit to compare consistency and inconsistency models, *P* values from statistical tests, or summary of inconsistency estimates	12,Table S8

Section/Topic	Item	Checklist Item	Reported on Page
		from different parts of the treatment network.	
Risk of bias across studies	22	Present results of any assessment of risk of bias across studies for the evidence base being studied.	12, Figure S2 Figure S3
Results of additional analyses	23	Give results of additional analyses, if done (e.g., sensitivity or subgroup analyses, meta-regression analyses*, alternative network geometries studied, alternative choice of prior distributions for Bayesian analyses,* and so forth).	10-11, Table S5 Table S6, Figure S5, Figure S6,
**DISCUSSION**			
Summary of evidence	24	Summarize the main findings, including the strength of evidence for each main outcome; consider their relevance to key groups (e.g., healthcare providers, users, and policy-makers).	12-14
Limitations	25	Discuss limitations at study and outcome level (e.g., risk of bias), and at review level (e.g., incomplete retrieval of identified research, reporting bias). *Comment on the validity of the assumptions, such as transitivity and consistency. Comment on any concerns regarding network geometry (e.g., avoidance of certain comparisons).*	15
Conclusions	26	Provide a general interpretation of the results in the context of other evidence, and implications for future research.	15
**FUNDING**			
Funding	27	Describe sources of funding for the systematic review and other support (e.g., supply of data); role of funders for the systematic review. This should also include information regarding whether funding has been received from manufacturers of treatments in the network and/or whether some of the authors are content experts with professional conflicts of interest that could affect use of treatments in the network.	17

Table S2.Study and Patient Characteristics

Study	Trial name	NCTnumber	Phase	Tumor type	Region	line of treatment	Disease Stage	Treatment	Sample Size	Age (range)	male (n,%)	Follow-up (months)	pulmonary metastasis	Previous radiation therapy	Former smoker	Current smoker
Ascierto,2020	CheckMate 238	NCT02388906	III	Melanoma	NA	NA	Stage IIIB, IIIC, or IV	Niv 3mg/kg q2w	453	56 (19-83)	258 (57.0)	51.1	NA	NA	NA	NA
								Ipi 10mg/kg q3w	453	54 (18-86)	269 (59.4)	51.1	NA	NA	NA	NA
Bellmunt,2021	IMvigor010	NCT02450331	III	UC	MN	all	M0 and either ypT2–4a or ypN+	Ate 1200mg q3w	406	67 (60-72)	322 (79)	21.9	NA	NA	NA	NA
								Placebo	403	66 (60-73)	316 (78)	21.9	NA	NA	NA	NA
Emens,2021	IMpassion130	NCT02425891	III	TNBC	MN	all	Unresectable, locally advanced or metastatic	Ate 840mg q2w+Chemo	451	55 (46-64)	3 (1)	18.8	226 (50.1)	268 (59.4)	NA	NA
								Chemo	451	56 (47-65)	1 (<1)	18.8	242 (53.7)	280 (62.1)	NA	NA

Chen,2020	ATTRACTION-2	NCT02267343	III	G/GEJ	MN	≥2	Unresectable advanced or recurrent	Niv 3mg/kg q2w	330	62 (54-69)	229 (69)	27.3	18 (5%)	NA	NA	NA
								Placebo	163	61 (53-68)	119 (73)	27.3	6 (4%)	NA	NA	NA
1Choueiri,2021	CheckMate 9ER	NCT03141177	III	RCC	MN	1	Advanced	Niv 240mg q2w+Cabozantinib	323	62 (29-90)	249 (77.1)	18.1	238 (73.7)	46 (14.2)	NA	NA
								Sunitinib	328	61 (28-86)	232 (70.7)	18.1	249 (75.9)	45 (13.7)	NA	NA
Cortes,2020	KEYNOTE-355	NCT02819518	III	TNBC	MN	1	Locally recurrent	Pem 200mg q3w+Chemo	566	53 (44-63)	NA	25.9	324 (57%)	NA	NA	NA
								Chemo	281	53 (43-63)	NA	26.3	162 (58%)	NA	NA	NA
Finn,2020	KEYNOTE-240	NCT02702401	III	HCC	MN	2	Stage C disease or stage	Pem 200mg q3w	278	67 (18-91)	226 (81.3)	13.8	NA	NA	NA	NA
								Placebo	135	65 (23-89)	112 (83)	10.6	NA	NA	NA	NA
Fuchs,2020	KEYNOTE-061	NCT02370498	III	G/GEJ	MN	≥2	Advanced	Pem 200mg q3w	296	62.5 (54-70)	202 (68)	27	NA	NA	NA	NA
								Paclitaxel	296	60.0 (53-68)	208 (70)	27	NA	NA	NA	NA

Gutzmer,2020	IMspire150	NCT02908672	III	Melanoma	MN	1	Stage IIIc–IV	Ate 840mg q2w+ Cobimetinib + Vemurafenib	256	54.0 (44.8-64.0)	150 (59)	18.9	NA	NA	NA	NA
								placebo+ Cobimetinib + Vemurafenib	258	53.5 (43.0-63.8)	149 (58)	18.9	NA	NA	NA	NA
Herbst,2020	IMpower110	NCT02409342	III	NSCLC	MN	1	Stage IV	Ate 1200mg q3w	277	64 (30-81)	196 (70.8)	13.4	NA	NA	166 (59.9)	74 (26.7)
								Chemo	277	65 (30-87)	193 (69.7)	13.4	NA	NA	161 (58.1)	81 (29.2)
Hodi,2018	CheckMate 067	NCT01844505	III	Melanoma	MN	1	Stage III (unresectable) or stage IV	Niv 1mg/kg q3w+Ipi3 mg/kg q3w	314	61 (18–88)	206 (66)	46.9	NA	NA	NA	NA
								Niv 3mg/kg q2w	316	60 (25–90)	202 (64)	36	NA	NA	NA	NA
								Ipi 3mg/kg q3w	315	62 (18–89)	202 (64)	18.6	NA	NA	NA	NA
Huang,2020	NA	NCT03099382	III	OSCC	China	2	Advanced or metastatic	Cam 200mg q2w	228	60 (54-65)	208 (91)	8.3	107 (47%)	155 (68%	NA	NA

														)		
								Chemo	220	60 (54-65)	192 (87)	6.2	94 (43%)	138 (63%)	NA	NA
Janjigian,2021	CheckMate 649	NCT02872116	III	G/GEJ	MN	1	Advanced	Niv 360mg q3w or 240mg q2w+Chemo	789	62 (54-69)	540 (68)	min12.1	NA	NA	NA	NA
								Chemo	792	61 (53-68)	560 (71)	min12.1	NA	NA	NA	NA
Jotte,2020	IMpower131	NCT02367794	III	NSCLC	MN	1	Stage IV	Ate 1200mg q3w+Carboplatin+Paclitaxel	338	64 (43-85)	278 (82.2)	NA	NA	NA	308 (91.1)	
								Ate 1200mg q3w+Carboplatin+Nab-Paclitaxel	343	65 (23-83)	280 (81.6)	26.8	NA	NA	311 (90.7)	
								Carboplatin+Nab-Paclitaxel	340	65 (38-86)	277 (81.5)	24.8	NA	NA	316 (92.9)	

Kuruvilla,2021	KEYNOTE-204	NCT02684292	III	Classical Hodgkin lymphoma	MN	all	Relapsed or refractory	Pem 200mg q3w	151	36 (28-53)	84 (56)	25.7	NA	58 (38%)	NA	NA
								Brentuximab Vedotin	153	35 (28-50)	90 (59)	25.7	NA	61 (40%)	NA	NA
1Wu,2019	CheckMate 078	NCT02613507	III	NSCLC	MN	≥2	Stage IIIB or IV	Niv 3mg/kg q2w	338	60 (27-78)	263 (78)	10.4	NA	NA	236 (70)	
								Docetaxel	166	60 (38-78)	134 (81)	8.8	NA	NA	118 (71)	
Miles,2021	IMpassion131	NCT03125902	III	TNBC	Global	1	Unresectable locally advanced/metastatic	Chemo	220	53 (25-81)	0	14.5	100 (45)	NA	NA	NA
								Ate 840mg q2w+Chemo	431	54 (22-85)	1 (<1)	14.2	230 (53)	NA	NA	NA
Mittendorf,2020	IMpassion031	NCT03197935	III	TNBC	MN	1	Early-stage	Ate 840mg q2w+Chemo	165	51 (22-76)	165 (100)	20.6	NA	NA	NA	NA
								Chemo	168	51 (26-78)	168 (100)	19.8	NA	NA	NA	NA
Moore,2021	IMagyn050	NCT03038100	III	OC	MN	1	Stage III or IV	Ate 1200mg q3w	651	60 (29-84)	/	19.9	NA	NA	NA	NA

								Chemo+Bevacizumab	650	59 (18-83)	/	19.8	NA	NA	NA	NA
1Motzer,2020	CheckMate 025	NCT01668784	III	RCC	MN	≥2	Advanced or metastatic	Niv 3mg/kg q2w	410	62 (23-88)	315 (77)	72	278	NA	NA	NA
								Everolimus	411	62 (18-86)	304 (74)	72	274	NA	NA	NA
Fradet,2019	KEYNOTE-045	NCT02256436	III	UC	MN	2	Advanced	Pem 200mg q3w	270	67 (29-88)	200 (74.1)	27.7	NA	NA	136 (50.4)	29 (10.7)
								Chemo	272	65 (26-84)	202 (74.3)	27.7	NA	NA	148 (54.4)	38 (14.0)
Antonia,2017	PACIFIC	NCT02125461	III	NSCLC	MN	≥2	Stage III	Dur 10mg/kg q2w	473	64 (31-84)	334 (70.2)	14.5	NA	NA	354 (74.4)	79 (16.6)
								Placebo	236	63 (23-90)	166 (70.0)	14.5	NA	NA	98 (41.4)	38 (16.0)
1Powles,2020	DANUBE	NCT02516241	III	UC	MN	1	Unresectable, locally advanced or metastatic	Dur 1500mg q4w	346	67 (60-73)	249 (72)	41.2	NA	NA	159 (46)	60 (17)

								Dur 1500mg q4w+Tre 75mg q4w	342	68 (60-73)	256 (75)	41.2	186 (54)	NA	176 (51)	66 (19)
								Chemo	344	68 (60-73)	274 (80)	41.2	178 (52)	NA	178 (52)	61 (18)
2Powles,2020	KEYNOTE-426	NCT02853331	III	RCC	MN	1	Advanced	Pem 200mg q3w+Axitinib	432	62 (55-68)	308 (71)	30.6	312 (72%)	41 (9%)	NA	NA
								Sunitinib	429	61 (53-68)	320 (75)	30.6	309 (72%)	40 (9%)	NA	NA
Powles,2021	KEYNOTE-361	NCT02853305	III	UC	MN	1	Advanced	Pem 200mg q3w+Chemo	351	69 (62-75)	272 (78)	31.7	NA	NA	NA	NA
								Pem 200mg q3w	307	68 (61-74)	228 (74)	31.7	NA	NA	NA	NA
								Chemo	352	69 (61-75)	262 (74)	31.7	NA	NA	NA	NA
Reardon,2020	CheckMate 143	NCT02017717	III	Glioblastoma	MN	≥2	Recurrent	Niv 3mg/kg q2w	184	55.5 (22-77)	116 (63.0)	9.5	NA	184 (100)	NA	NA
								Bevacizumab	185	55.0 (22-76)	119 (64.3)	9.5	NA	185 (100)	NA	NA

Reck,2021	CheckMate 9LA	NCT03215706	III	NSCLC	MN	1	Advanced(stage IV or recurrent)	Niv 360mg q2w+Ipi 1mg/kg q6w+Chemo	361	65 (35-81)	252 (70)	min24	NA	NA	315 (87)	
								Chemo	358	65 (26-86)	252 (70)	min24	NA	NA	306 (86)	
Rizvi,2020	MYSTIC	NCT02453282	III	NSCLC	MN	1	Stage IV	Dur 20mg/kg q4w	374	65.0 (28–84)	256 (68.4)	30.2	NA	NA	211 (56.4)	106 (28.3)
								Dur 20mg/kg q4w+Tre 1mg/kg q4w	372	66.0 (28–87)	266 (71.5)	30.2	NA	NA	233 (62.6	83 (22.3)
								Chemo	372	64.0 (30–85)	250 (67.2)	30.2	NA	NA	227 (61.0)	93 (25.0)
Rudin,2020	KEYNOTE-604	NCT03066778	III	SCLC	MN	1	Sstage IV	Pem 200mg q3w	228	64 (24-81)	152 (66.7)	30.5	NA	NA	72 (31.6)	148 (64.9)
								Etoposide	225	65 (37-83)	142 (63.1)	30.5	NA	NA	84 (37.3)	133 (59.1)
Sezer,2021	Empwer-Lung 1	NCT03088540	III	NSCLC	MN	1	Advanced	Cem 350mg q3w	356	63 (58-69)	312 (88)	13.1	NA	NA	223 (63%	133 (37%

															)	)
								Chemo	354	64 (57-69)	294 (83)	13.1	NA	NA	234 (66%)	120 (34%)
Shitara,2020	KEYNOTE-062	NCT02494583	III	Gastric Cancer	MN	1	Advanced	Pem 200mg q3w	256	61 (20-83)	180 (70.3)	29.4	NA	NA	NA	NA
								Pem 200mg q3w+Chemo	257	62 (22-83)	195 (75.9)	29.4	NA	NA	NA	NA
								Chemo	250	62.5 (23-87)	179 (71.6)	29.4	NA	NA	NA	NA
Sugawara,2021	ONO-4538-52	NCT03117049	III	NSCLC	MN	1	Stage IIIB/IV or recurrent	Niv 360mg q3w	275	66 (27-85)	205 (74.5)	13.7	NA	NA	196 (71.3)	18 (6.5)
								Placebo	275	66 (33-83)	206 (74.9)	13.7	NA	NA	200 (72.7)	21 (7.6)
Sun2021	KEYNOTE-590	NCT03189719	III	Oesophageal cancer	MN	1	Locally advanced, unresectable or metastatic	Pem 200mg q3w	373	64 (28-94)	306 (82)	22.6	NA	NA	NA	NA
								Chemo	376	62 (27-89)	319 (85)	22.6	NA	NA	NA	NA

Winer2021	KEYNOTE-119	NCT02555657	III	TNBC	MN	≥2	Metastatic	Pem 200mg q3w	312	50 (43-59)	0	31.4	NA	NA	NA	NA
								Chemo	310	53 (44-61)	2 (1)	31.5	NA	NA	NA	NA
Yang,2021	CAPTAIN-1st	NCT03707509	III	NC	China	1	Recurrent or metastatic	Cam 200mg q3w+Gemcitabine and Cisplatin	134	52 (40-58)	113 (84)	10.2	66 (49%)	86 (64%)	NA	NA
								Gemcitabine and Cisplatin	129	49 (40-56)	105 (81)	10.2	61 (47%)	83 (64%)	NA	NA
1Zhou,2021	ORIENT-12	NCT03629925	III	NSCLC	China	1	Stage IIIB/IIIC or stage IV	Sin 200mg q3w+GP	179	64 (39-75)	163 (91.1)	12.9	NA	8 (4.5)	103 (57.5)	52 (29.1)
								GP	178	62 (33-75)	164 (92.1)	12.9	NA	4 (2.2)	97 (54.5)	50 (28.1)
2Zhou,2021	CameL	NCT03134872	III	NSCLC	China	1	Advanced	Cam 200mg q3w+Carboplatin and Pemetrexed	205	59 (54-64)	146 (71)	11.9	NA	NA	NA	NA
								Chemo	207	61 (53-65)	149 (72)	11.9	NA	NA	NA	NA

Ferris,2017	CheckMate 141	NCT02105636	III	HNSCC	all	≥2	Recurrent	Niv 3mg/kg q2w	240	59 (29-83)	197 (82.1)	5.1	NA	NA	191 (79.6)	
								Chemo	121	61 (28-78)	103 (85.1)	5.1	NA	NA	85 (70.2)	
Brahmer,2015	CheckMate 017	NCT01642004	III	NSCLC	all	2	Stage IIIB or IV	Niv 3mg/kg q2w	135	62 (39-85)	111 (82)	min11	NA	NA	121 (90)	
								Chemo	137	64 (42-84)	97 (71)	min11	NA	NA	129 (94)	
Borghaei,2015	CheckMate 057	NCT01673867	III	NSCLC	NA	≥2	Stage IIIB or IV	Niv 3mg/kg q2w	292	61 (37-84)	151 (52)	13.2	NA	19 (12.6)	231 (79)	
								Chemo	290	64 (21-85)	168 (58)	13.2	NA	NA	227 (78)	
Reck,2019	KEYNOTE-024	NCT2142738	III	NSCLC	MN	1	untreated stage IV	Pem 200mg q3w	154	64.5 (33-90)	92 (59.7)	25.2	NA	NA	115 (74.7)	34 (22.1)
								Chemo	151	66.0 (38-85)	95 (62.9)	25.2	NA	NA	101 (66.9)	31 (20.5)
1Robert,2015	KEYNOTE-006	NCT01866319	III	Melanoma	MN	all	Stage III or IV	Pem 10mg/kg q2w	279	61 (18-89)	161 (58)	7.9	NA	NA	NA	NA
								Pem 10mg/kg q3w	277	63 (22-89)	174 (63)	7.9	NA	NA	NA	NA

								Ipi 3 mg/kg q3w	278	62 (18-88)	162 (58)	7.9	NA	NA	NA	NA
Carbone,2017	CheckMate 026	NCT2041533	III	NSCLC	MN	1	Stage IV or Recurrent	Niv 3mg/kg q2w	271	63 (32-89)	184 (68)	13.5	NA	102 (38)	186 (69)	52 (19)
								Chemo	270	65 (29-87)	148 (55)	13.5	NA	107 (40)	182 (67)	55 (20)
Fehrenbacher, 2018	OAK	NCT02008227	III	NSCLC	MN	≥2	Stage IIIB or IV	Ate 1200mg q3w	613	63.0 (25-84)	379 (61.8)	min26	NA	NA	418 (68.2)	83 (13.5)
								Docetaxel	612	64.0 (34-85)	379 (61.9)	min21	NA	NA	409 (66.8)	107 (17.5)
Paz-Ares,2018	KEYNOTE-407	NCT02775435	III	NSCLC	MN	1	Stage IV	Pem 200mg q3w+Chemo	278	65 (29-87)	220 (79.1)	7.8	NA	NA	256 (92.1)	
								Chemo	281	65 (36-88)	235 (83.6)	7.8	NA	NA	262 (93.2)	
Mok,2019	KEYNOTE-042	NCT02220894	III	NSCLC	Global	1	Locally advanced or metastatic	Pem 200mg q3w	637	63.0 (57.0-69.0)	450 (71)	12.8	NA	75 (12%)	370 (58%)	125 (20%)
								Chemo	637	63.0 (57.0-69.0)	452 (71)	12.8	NA	81 (13%)	351 (55%)	146 (23%)

Paz-Ares,2019	CASPIAN	NCT03043872	III	SCLC	MN	1	Stage IV	Dur 1500mg q4w+Chemo	268	62 (58-68)	190 (71)	14.2	NA	NA	126 (47%)	120 (45%)
								Chemo	269	63 (57-68)	184 (68)	14.2	NA	NA	128 (48%)	126 (47%)
Hellmann,2018	CheckMate 227	NCT02477826	III	NSCLC	all	1	Stage IV or recurrent	Niv 1mg/kg q2w+Ipi 1mg/kg q6w	583	64 (26-87)	391 (67)	11.2	NA	NA	85	
								Niv 240mg q2w	396	64 (27-85)	273 (68)	11.2	NA	NA	86	
								Chemo	583	64	385 (66)	11.2	NA	NA	86	
Gandhi,2018	KEYNOTE-189	NCT02578680	III	NSCLC	MN	1	Metastatic	Pem 200mg q3w+Chemo	410	65.0 (34.0-84.0)	254 (62.0)	10.5	NA	NA	362 (88.3)	
								Chemo	206	63.5 (34.0-84.0)	109 (52.9)	10.5	NA	NA	181 (87.9)	
Eggermont,2015	EORTC 18071	NCT00636168	III	Melanoma	MN	≥2	Stage III	Ipi 10 mg/kg q3w	475	51 (20-84)	296 (62)	32.9	NA	NA	NA	NA
								Placebo	476	52	293	32.9	NA	NA	NA	NA

										(18-78)	(62)			
Eng,2019	IMblaze370	NCT02788279	III	CRC	MN	≥2	Advanced or	Ate 840mg q2w+Cobimetinib	183	58 (51-67)	107 (58)	7.3	NA	NA	NA	NA
								Ate 1200mg q3w	90	56 (51-64)	59 (66)	7.3	NA	NA	NA	NA
								Regorafenib	90	59 (52-66)	51 (57)	7.3	NA	NA	NA	NA
Cohen,2019	KEYNOTE-040	NCT02252042	III	HNSCC	MN	≥2	Recurrent or metastatic	Pem 200mg q3w	247	60.0 (55-66)	207 (84)	7.5	NA	NA	147	32
								Placebo	248	60.0 (54-66)	205 (83)	7.1	NA	NA	146	36
Eggermont,2018	KEYNOTE-054	NCT02362594	III	Melanoma	MN	1	Stage III	Pem 200mg q3w	514	54 (19-88)	324 (63.0)	15	NA	NA	NA	NA
								Placebo	505	54 (19-83)	304 (60.2)	15	NA	NA	NA	NA
Long,2019	KEYNOTE-252	NCT02752074	III	Melanoma	MN	all	Unresectable stage III or IV	Pem 200mg q3w+Epacadostat	354	64 (52−72)	217 (61)	12.4	NA	NA	NA	NA
								Pem 200mg q3w	352	63 (53.5−72)	206 (59)	12.4	NA	NA	NA	NA
Reck,2016	CA184-156	NCT01450761	III	SCLC	MN	all	Extensive-stage	Ipi 10mg/kg q3w+Chemo	478	62 (39-85)	317 (66)	10.5	NA	NA	NA	NA
								Chemo	476	63 (36-81)	326 (68)	10.2	NA	NA	NA	NA

Socinski,2018	IMpower150	NCT2366143	III	NSCLC	MN	1	Stage IV or recurrent	Ate 1200mg q3w+TTD+Chemo	400	63 (31−89)	240 (60.0)	20	NA	NA	228 (57.0)	90 (22.5)
								TTD+chemo	400	63 (31−90)	239 (59.8)	20	NA	NA	231 (57.8)	92 (23.0)
Kojima,2020	KEYNOTE-181	NCT02564263	III	Esophageal Cancer	Global	all	Etastatic or locally advanced, unresectable	Pem 200mg q3w	314	63.0 (23-84)	273 (86.9)	7.1	NA	NA	NA	NA
								Chemo	314	62.0 (24-84)	271 (86.3)	6.9	NA	NA	NA	NA
Finns,2020	IMbrave-150	NCT03434379	III	HCC	Global	1	Unresectable	Ate 1200mg q3w	336	64 (56-71)	277 (82)	8.9	NA	NA	NA	NA
								Sorafenib	165	66 (59-71)	137 (83)	8.1	NA	NA	NA	NA
Yang,2019	CAURAL	NCT02454933	III	NSCLC	MN	all	Stage IIIB–IV	Osimertinib	17	65 (41-80)	4 (24)	23.9	NA	NA	4	0
								Dur 10mg/kg q2w+Osimertinib	12	56 (41-78)	6 (50)	17.1	NA	NA	3	1
Horn,2018	IMpower133	NCT2763579	III	SCLC	MN	1	Extensive-stage	Ate 1200mg q3w+Chemo	201	64 (28-90)	129 (64.2)	13.9	NA	25 (12.4)	118 (58.7)	74 (36.8)
								Chemo	202	64	132	13.9	NA	28	124	75

										(26-87)	(65.3)			(13.9)	(61.4)	(37.1)
Owonikoko,2021	CheckMate 451	NCT02538666	III	SCLC	MN	≥2	Extensive-Disease	Niv 1mg/kg q3w+ Ipi 3mg/kg q3w	279	64.0 (39-85)	180 (64.5)	8.4	NA	NA	257 (92.1)	
								Niv 240mg q2w	280	65.0 (32-84)	177 (63)	9.9	NA	NA	257 (91.8)	
								Placebo	275	64.0 (44-84)	175 (63.6)	9.1	NA	NA	259 (94.2)	
2Wu,2020	KEYNOTE-042 China Study	NCT03850444	III	NSCLC	china	1	PD-L1-positive locally advanced or metastatic	Pem 200mg q3w	128	62 (22-78)	105 (82)	33	NA	6 (4.7)	69 (53.9)	28 (21.9)
								Chemo	134	62 (32-82)	119 (88.8)	33	NA	4 (3.0)	77 (57.5)	29 (21.6)
Gogas,2020	IMspire170	NCT03273153	III	Melanoma	MN	1	Locally advanced and	Pem 200mg q3w	224	66 (55-73)	141 (62.9)	7.2	NA	NA	NA	NA
								Ate 840mg q2w+TTD	222	66 (54-73)	129 (58.1)	7.1	NA	NA	NA	NA

Powlers,2018	IMvigor211	NCT02302807	III	UC	MN	≥2	Platinum-treated locally advanced or metastatic	Ate 1200mg q3w	467	67 (33-88)	357 (76)	17.3	NA	NA	266 (57%)	60 (13%)
								Chemo	464	67 (31-84)	361 (78)	17.3	NA	NA	280 (61%)	60 (13%)
Barlesi,2018	JAVELIN Lung 200	NCT02395172	III	NSCLC	MN	≥2	Platinum-treated advanced	Ave 10mg/kg q2w	396	64 (58-69)	269 (68)	18.9	NA	NA	324 (82%)	
								Docetaxel	396	63 (57-69)	273 (69)	17.8	NA	NA	333 (84%)	
Burtness,2019	KEYNOTE-048	NCT02358031	III	HNSCC	MN	1	Recurrent or metastatic	Pem 200mg q3w	301	62 (56-68)	250 (83)	11.5	NA	NA	239 (79%)	
								Pem 200mg q3w+Chemo	281	61 (55-68)	224 (80)	13	NA	NA	224 (80%)	
								TTD+Chemo	300	61 (54.5-68.0)	261 (87)	10.7	NA	NA	234 (78%)	
Bajorin,2021	CheckMate 274	NCT02632409	III	UC	MN	all	A high risk of recurrence	Niv 240mg q2w	353	65.3 (30-92)	265 (75.1)	20.9	NA	NA	237	
								Placebo	356	65.9	275	19.5	NA	NA	247	

										(42-88)	(77.2)					
Fennell,2021	CONFIRM	NCT03063450	III	Malignant mesothelioma	UK	≥2	Relapsed	Niv 240mg q2w	221	70 (65-74)	167 (76)	11.6	NA	NA	105 (48%)	15 (7%)
								Placebo	111	71 (65-76)	86 (78)	11.6	NA	NA	52 (47%)	6 (5%)
Boyer,2021	KEYNOTE-598	NCT03302234	III	NSCLC	MN	1	Stage IV	Pem 200mg q3w+Ipi 1mg/kg q6w	284	64 (35-85)	202 (71.1)	20.6	NA	60 (21.1)	197 (69.4)	58 (20.4)
								Pem 200mg q3w	284	65 (35-85)	191 (67.3)	20.6	NA	57 (20.1)	183 (64.4)	76 (26.8)
Usmani,2019	KEYNOTE-185	NCT02579863	III	Multiple myeloma	MN	1	Newly diagnosed,treatment-naive, active multiple	Pem 200mg q3w+Lenalidomide and Dexamethasone	151	74 (70-79)	70 (46)	6.6	NA	NA	NA	NA
								Lenalidomide and Dexamethasone	150	74 (70-78)	71 (47)	6.6	NA	NA	NA	NA
Hamanishi,2021	NINJA		III	OC	Japan	≥2	Advanced or recurrent	Niv 240 mg q2w	157	58 (29-84)	0	1	NA	NA	NA	NA
								GEM or PLD	159	60 (34-80)	0	1	NA	NA	NA	NA

Wang,2021	BGB-A317-307	NCT03594747	III	SCLC	China	1	Advanced	Tis 200mg q3w+PC	120	60 (41-74)	107 (89.2)	8.6	NA	NA	96 (80.0)
								Tis 200mg q3w+nab-PC	119	63 (38-74)	112 (94.1)	8.6	NA	NA	107 (89.9)
								PC	121	62 (34-74)	111 (91.7)	8.6	NA	NA	98 (81.0)
2Robert,2015	CheckMate 066	NCT01721772	III	Melanoma	MN	1	Stage III or IV	Niv 3mg/kg q2w	210	64 (18-86)	121 (57.6)	8.9	NA	NA	NA	NA
								Chemo	208	66 (26-87)	125 (60.1)	6.8	NA	NA	NA	NA
Govindan,2017	CA184-104	NCT01285609	III	NSCLC	MN	1	Stage IV	Ipi 10mg/kg q3w+Chemo	388	64 (28-84)	326 (84)	12.5	NA	NA	NA	NA
								Chemo	361	64 (28-85)	309 (52)	11.8	NA	NA	NA	NA
Schmid,2020	KEYNOTE-522	NCT03036488	III	TNBC	MN	1	Stage II or Stage III	Pem 200mg q3w+Chemo	784	49 (22-80)	1 (<1)	15.5	NA	NA	NA	NA
								Chemo	390	48 (24-79)	0	15.5	NA	NA	NA	NA
Planchard,2020	ARCTIC	NCT02352948	III	NSCLC	MN	≥2	Stage IIIB/IV locally advanced or metastic	Dur 10mg/kg q2w	62	63.5 (35-79)	42 (67.7)	9.1	NA	NA	47 (75.8)	5 (8.1)
								Soc	64	62.0	48	9.1	NA	NA	45	10

										(41-81)	(75.0)				(70.3)	(15.6)
								Dur 20mg/kg q4w+Tre 1mg/kg q4w	174	62.5 (26-81)	115 (66.1)	9.1	NA	NA	109 (62.6)	30 (17.2)
								Soc	118	65.0 (42-83)	81 (68.6)	9.1	NA	NA	74 (62.7)	22 (18.6)
								Dur 10mg/kg q2w	117	63.0 (19-83)	73 (62.4)	9.1	NA	NA	67 (57.3)	22 (18.8)
								Tre 10mg/kg q4w	60	63.5 (45-81)	39 (65.0)	9.1	NA	NA	38 (63.3)	8 (13.3)
Yang,2020	CIBI308C30 2	NCT03607 539	III	NSCLC	chin a	1	Stage IIIB to IV	Sin 200mg q3w+Chemo	266	61 (30-75)	204 (76.7)	8.9	NA	NA	122 (45.9)	49 (18.4)
								Chemo	131	61 (35-75)	99 (75.6)	8.9	NA	NA	64 (48.9)	23 (17.6)
Weber,2015	CheckMate 037	NCT01721 746	III	Melanoma	MN	2	Stage IIIC or IV	Niv 3mg/kg q2w	272	59 (23-88)	176 (65)	8.4	NA	NA	NA	
								Chemo	133	62 (29-85)	85 (64)	8.4	NA	NA	NA	NA

Ferris,2020	EAGLE	NCT02369874	III	HNSCC	MN	≥2	progression or recurrence	Dur 10mg/kg q2w	240	59.0 (24-84)	202 (84.2)	7.6	NA	NA	153 (63.8)	41 (17.1)
								Dur 20mg/kg q4w+Tre 1mg/kg q4w	247	61.0 (23-81)	209 (84.6)	6.3	NA	NA	146 (59.1)	45 (18.2)
								Chemo	249	61.0 (22-82)	207 (83.1)	7.8	NA	NA	140 (56.2)	56 (22.5)
Pujade-Lauraine,2021	JAVELIN OVARIAN 200	NCT02580058		OC	MN	≥2	Platinum-resistant or platinum-refractory	Ave 10mg/kg q2w	188	61 (53-69.5)	0	18.2	NA	NA	NA	NA
								Ave 10mg/kg q2w+PLD	188	60 (53-67)	0	18.4	NA	NA	NA	NA
								PLD	190	60 (53-69)	0	17.4	NA	NA	NA	NA
Rini,2019	IMmotion151	NCT02420821	III	RCC	MN	1	Advanced	Ate 1200mg q3w+Bevacizumab	454	62 (56-69)	317 (70)	24	339 (75%)	NA	NA	NA
								Sunitinib	461	60 (54-66)	352 (76)	24	325 (71%)	NA	NA	NA
Beer,2016	CA184-095	NCT01057810	III	Prostate Cancer	, MN	1	Metastatic	Ipi 10mg/kg q3w	400	70 (44-91)	400 (100)	24	NA	NA	NA	NA

								Placebo	202	69 (42-92)	202 (100)	24	NA	NA	NA	NA
Mario,2015	CA184-024	NCT00324155	III	Melanoma	, MN	1	Stage IIIc, N3 (unresectable), or Stage IV	Ipi 10mg/kg q3w+Dacarbazine	250	57.5	152 (60.8)	54	NA	NA	NA	NA
								Dacarbazine	252	56.4	149 (59.1)	54	NA	NA	NA	NA
2Motzer,2020	CheckMate 214	NCT02231749	III	RCC	Global	1	Advanced	Niv 3mg/kg q3w+Ipi 1mg/kg q3w	550	62 (26-85)	413 (75)	42	381 (69)	63 (11)	NA	NA
								Sunitinib	546	62 (21-85)	395 (72)	42	373 (68)	70 (13)	NA	NA
West,2019	IMpower130	NCT02367781	III	NSCLC	, MN	1	Stage IV	Ate 1200mg q3w+carboplatin+nab-paclitaxel	483	64 (18-86)	277 (57)	18.5	NA	NA	323 (67%)	96 (20%)
								Chemo	240	65 (38-85)	138 (58)	18.8	NA	NA	167 (70%)	53 (22%)
Baas,2021	CheckMate 743	NCT02899299	III	Malignant pleural mesothelioma	Global	1	Unresectable	Niv 3mg/kg q2w+Ipi 1mg/kg q6w	303	69 (65-75)	234 (77)	29.7	NA	29 (10%)	173 (57%)	
								Chemo	302	69	233	29.7	NA	28	171 (57%)	

										(62-75)	(77)			(9%)		
Lee,2021	JAVELIN HEAD AND NECK 100	NCT02952586	III	HNSCC	MN	1	Locally advanced	Ave 10mg/kg q2w+Chemoradiotherapy	350	60 (54-65)	290 (83)	16.7	NA	NA	NA	NA
								Chemoradiotherapy	347	59 (54-65)	285 (82)	16.8	NA	NA	NA	NA
Moehler,2021	JAVELIN Gastric 100	NCT02625610	III	Gastric Cancer	Global	1	Advanced or metastatic	Ave 10mg/kg q2w	249	62	164 (65.9)	24.1	NA	NA	NA	NA
								Chemo	250	61	167 (66.8)	24	NA	NA	NA	NA
Kato,2019	ATTRACTION-3	NCT02569242	III	OSCC	MN	2	Unresectable advanced or recurrent	Niv 240mg q2w	210	64 (57-69)	179 (85)	min17.6	98 (47%)	153 (73%)	159 (76%)	21 (10%)
								Chemo	209	67 (57-72)	185 (89)	min17.6	92 (44%)	142 (68%)	147 (70%)	30 (14%)
Shitara,2018	KEYNOTE-061	NCT02370498	III	G/GEJ	MN	≥2	Advanced	Pem 200mg q3w	296	62.5 (54-70)	202 (68)	7.9	NA	NA	NA	NA
								Paclitaxel	296	60.0 (53-68)	208 (70)	7.9	NA	NA	NA	NA
2Choueiri,2021	KEYNOTE-564	NCT03142334	III	RCC	MN	NA	High risk for recurrence	Pem 200mg q3w	488	60.0 (27–81)	347 (70.0)	24.1	NA	NA	NA	NA
								Placebo	496	60.0	359	24.1	NA	NA	NA	NA

										(25−84)	(72.1)					
Mateos,2019	KEYNOTE-183	NCT02576977	III	Multiple Myeloma	MN	≥2	Active multiple	Pem 200mg q3w+Pomalidomide+Dexamethasone	125	65 (60-72)	77 (62)	8.1	NA	NA	NA	NA
								Pomalidomide+Dexamethasone	124	67 (60-74)	78 (63)	8.1	NA	NA	NA	NA
Gettinger,2021	Lung-MAP S1400I	NCT02785952	III	SCLC	MN	1	Stage IV	Niv 3mg/kg q2w+Ipi 1mg/kg q6w	125	67.5 (41.8-83.4)	83 (66)	29.5	NA	41 (33)	75 (60)	48 (38)
								Niv 3mg/kg q2w	127	68.1 (48.6-90.3)	86 (68)	29.5	NA	45 (35)	72 (57)	54 (43)
Motzer,2021	KEYNOTE-581	NCT02811861	III	RCC	MN	1	Advanced	Pem 200mg q3w+Lenvatinib	355	64 (34-88)	255 (71.8)	26.6	249 (70.1)	NA	NA	NA
								Lenvatinib+Everolimus	357	62 (32-86)	266 (74.5)	26.6	245 (68.6)	NA	NA	NA
								Sunitinib	357	61 (29-82)	275 (77.0)	26.6	239 (66.9)	NA	NA	NA
Lu,2021	RATIONALE 304	NCT03663205	III	NSCLC	china	1	Stage IIIB or IV	Tis 200mg q3w+Chemo	223	60 (27-75)	168 (75.3)	9.8	NA	NA	115 (51.6)	32 (14.3)

								Chemo	111	61 (25-74)	79 (71.2)	9.8	NA	NA	53 (47.7)	13 (11.7)
Monk,2021	JAVELIN OVARIAN 100	NCT02718417	III	OC	Global	1	Stage III–IV	Chemotherapy followed by Ave 10mg/kg q2w	332	59.0 (52.0-67.0)	0	10.8	NA	NA	NA	NA
								Ave 10mg/kg q3w+Chemo followed by avelumab	331	60.0 (50.0-66.0)	0	10.8	NA	NA	NA	NA
								Chemo	335	57.0 (49.0-66.0)	0	10.8	NA	NA	NA	NA
NCT02481830	CheckMate 331	NCT02481830	III	SCLC	NA	≥2	Relapsed	Niv 240mg q2w	284	61.5	173 (61.3	15.8	NA	NA	NA	NA
								Chemo	285	61.6	165 (62.1)	15.8	NA	NA	NA	NA
NCT03361865	KEYNOTE-672	NCT03361865	III	UC	NA	NA	NA	Pem 200mg q3w+TTD	43	73.3	32 (75)	NA	NA	NA	NA	NA
								Pem 200mg q3w	49	72.4	38 (77.6)	NA	NA	NA	NA	NA

NCT03358472	KEYNOTE-669	NCT03358472	III	HNSCC	NA	NA	NA	Pem 200mg q3w+TTD	34	62.1	29 (85.7)	NA	NA	NA	NA	NA
								Pem 200mg q3w	19	63	16 (84.2)	NA	NA	NA	NA	NA
								Chemo	34	62.7	28 (82.9)	NA	NA	NA	NA	NA
NCT02279732	NA	NCT02279732	III	NSCLC	NA	NA	NA	Ipi 10mg/kg q3w+Chemo	98	60.9	87 (88.8)	NA	NA	NA	NA	NA
								Chemo	106	59.8	93 (87.7)	NA	NA	NA	NA	NA
Chan,2021	KEYNOTE-122	NCT02611960	III	NC	NA	NA	NA	Pem 200mg q3w	116	NA	NA	NA	NA	NA	NA	NA
								Chemo	112	NA	NA	NA	NA	NA	NA	NA
Zhou,2020	KEYNOTE-033	NCT02864394	III	NSCLC	NA	NA	NA	Pem 200mg q3w	213	NA	NA	NA	NA	NA	NA	NA
								Chemo	198	NA	NA	NA	NA	NA	NA	NA
NCT02576509	CheckMate 459	NCT02576509	III	HCC	MN	1	Advanced	Niv 240mg q2w	371	65 (57-71)	314 (85)	15.2	NA	45 (12%)	NA	NA
								Sorafenib	372	65 (58-72)	317 (85)	15.2	NA	40 (11%)	NA	NA

NCT02576509	IMbassador250	NCT03016312	III	mCRPC	NA	NA	Metastatic	Ate 1200mg q3w+Enzalutamide	379	70.0 (51-91)	379 (100)	15.2	NA	NA	NA	NA
								Enzalutamide	380	70.0 (40-92)	380 (100)	15.2	NA	NA	NA	NA
Peters,2021	CheckMate-032	NCT02046733	II	SCLC	MN	≥2	Stage IA-IIIB	Niv 1mg/kg+Ipi 3mg/kg q3w	78	61.1 (37.7-83.2)	50 (64.1)	22.4	NA	NA	51 (65.4)	27 (34.6)
								Placebo	75	61.9 (38.6-77.3)	42 (56.0)	22.4	NA	NA	49 (65.3)	25 (33.3)
Scherpereel,2019	IFCT-1501 MAPS2	NCT02716272	II	Malignant pleural mesothelioma	MN	≥2	Relapsed	Niv 3mg/kg q2w	63	72.3 (32.5-87.2)	47 (75.0)	20.1	NA	NA	NA	34 (54%)
								Niv 3mg/kg q2w+Ipi 1mg/kg q6w	61	71.2 (48.1-88.1)	53 (85.0)	20.1	NA	NA	NA	36 (58%)
Voss,2022	NA	NCT02118337	II	RCC	MN	2	Advanced or Metastatic	MEDI0680 20mg/kge+Dur 750mg q2w	42	64.0 (39-80)	33 (78.6)	38.3	NA	15 (35.7)	NA	NA
								Niv 240mg q2w	21	58.0 (38-80)	15 (71.4)	14.1	NA	5 (23.8)	NA	NA

Loibl,2019	GeparNuevo	NCT02685059	II	TNBC	MN	NA	Early	Dur 750mg q2w	92	49.5(25.0-74.0)	NA	NA	NA	NA	NA	NA
								placebo	82	49.5(23.0-76.0)	NA	NA	NA	NA	NA	NA
McDermott,2018	IMmotion150	NCT01984242	II	RCC	MN	NA	Metastatic	Sunitinib 50mg qd	101	61 (25-85)	79 (78)	20.7	NA	NA	NA	NA
								Ate 1200mg q3w	103	62 (32-88)	74 (73)	20.7	NA	NA	NA	NA
								Ate 1200mg + Bev 15mg/kg q3w	101	61 (27-81)	77 (75)	20.7	NA	NA	NA	NA
Zhou,2023	RATIONALE-303	NCT03358875	III	NSCLC	Global	≥2	Advanced	Tislelizumab 200 mg q3w	534	61.0 (28-88)	416 (77.8)	16	NA	NA	373 (69.7)	
								Docetaxel	258	61.0 (32-81)	206 (76.3)	10.7	NA	NA	188 (69.6)	
Johnson,2023	POSEIDON	NCT03164616	III	NSCLC	Global	1	Metastatic	Tre 75mg+Dur 1500mg+Chemo	330	63.0 (27-87)	269 (79.6)	10.3	NA	NA	195 (57.7)	84 (24.9)
								Dur 1500mg+Chemo	334	64.5 (32-87)	253 (74.9)	10.3	NA	NA	190 (56.2)	64 (18.9)
								Chemo	333	64.0	248	10.3	NA	NA	191	66

										(32-84)	(73.6)				(56.7)	(19.6)
Forde,2022	CheckMate 816	NCT02998 528	III	NSCLC	MN	≥2	Stage IB-IIIA	Niv 360mg q3w+Chemo	176	64 (41-82)	128 (71.5)	29.5	NA	NA	160 (89.4)	
								Chemo	176	65 (34-84)	127 (70.9)	29.5	NA	NA	158 (88.3)	
Jr,2023	NEPTUNE	NCT02542 293	III	NSCLC	MN	1	Metastatic	Dur 20 mg/kg+Tremelimumab 1 mg/kg q4w	410	63.0 (27-83)	297 (72.4)	32.9	NA	NA	200 (48.8)	138 (33.7)
								Chemo	339	65.0 (30-90)	305 (73.8)	32.9	NA	NA	222 (53.8)	117 (28.3)
Herbst,2022	COAST	NCT03822 351	II	NSCLC	Glo bal	NA	Unresectable,Sta ge III	Dur 1500mg q4w	66	66.0 (46-81)	45(6 7.2)	11.5	NA	62 (92.5)	63 (94.0)	
								Dur 1500mg q4w+Oleclumab	59	65.0 (37-83)	42(7 0.0)	11.5	NA	54 (90.0)	54 (90.0)	
								Dur 1500mg q4w+Monalizumab	61	65.0 (44-87)	44(6 7.7)	11.5	NA	57 (91.9)	59 (95.2)	

Diaz,2022	KEYNOTE-177	NCT02563002	III	colorectal cancer	MN	1	Metastatic	Pem 200 mg q3w	149	61.9 (14.9)	71(46.4)	44.5	NA	NA	NA	NA
								Chemo	142	60.6 (14.8)	82(53.2)	44.5	NA	NA	NA	NA
Xu,2022	ORIENT-2	NCT03116152	II	esophageal squamous cell carcinoma	MN	2	Advanced or Metastatic	Sin 200mg q3w	94	60 (54-64)	88 (92.6)	7.2	37 (38.9%)	50 (52.6%)	NA	NA
								Chemo	87	60 (54-64)	84 (88.4)	6.2	46 (48.4%)	58 (61.1%)	NA	NA
Taniguchi,2022	TORG1630	jRCTs031180331	III	NSCLC	MN	≥2	Advanced or Recurrent	Niv 240mg q2w	64	69.5 (35-84)	44 (68.8)	12.5	NA	NA	49 (76.6)	
								Niv 240mg q2w+Docetaxel	64	69 (45-83)	45 (70.3)	18.9	NA	NA	52 (81.3)	
Rossevold,2022	ALICE	NCT03164993	II	TNBC	MN	1	Metastatic	Chemo	28	52.5 (28.0-74.0)	0 (0)	32.2	10 (35.7%)	NA	NA	NA
								Ate 840 mg q2w+Chemo	40	58.5 (31.0-77.0)	1 (2.5)	32.2	18 (45.0%)	NA	NA	NA

Park,2022	NA	NCT02520453	II	esophageal squamous cell carcinoma	Korea	≥2	Stage II-III	Dur 20 mg/kg q4w	45	64 (39-76)	43 (96)	38.7	NA	NA	15 (33)	23 (51)
								Placebo	41	66 (42-83)	37 (90)	38.7	NA	NA	19 (46)	18 (44)
Cheng,2022	IMbrave150	NCT03434379	III	HCC	Global	1	Unresectable	Ate 1200mg+Bevacizumab q3w	329	NA	277(82.4)	15.6	NA	NA	NA	NA
								Sorafenib	156	NA	137(83.0)	15.6	NA	NA	NA	NA
Leighl,2022	CCTG BR34	NCT03793179	II	NSCLC	MN	1	Metastatic	Dur 1500 mg + Tre 75mg + Chemo q3w	148	65.0 (27-79)	81 (53.6)	16.6	NA	76 (50.3)	102 (67.6)	37 (24.5)
								Dur 1500 mg + Tre 75mg q3w	149	63.0 (38-87)	81 (54.0)	16.6	NA	77 (51.3)	101 (67.4)	35 (23.3)
Jung,2022	NA	NA	II	NSCLC	Korea	2	Advanced	Pem 200mg q3w+Chemo	47	63 (36-82)	37 (78.7)	10.5	NA	NA	36 (76.6%)	5 (10.6%)
								Placebo+Chemo	51	64 (38-79)	43 (84.3)	10.5	NA	NA	39 (76.5%)	5 (9.8%)

2Cheng,2022	ASTRUM-005	NCT04063163	III	SCLC	MN	1	Extensive-Stage	Serplulimab 4.5mg/kg q3w	389	63 (28-76)	317 (81.5)	12.3	NA	NA	102 (26.2)	206 (53.0)
								Placebo	196	62 (31-83)	164 (83.7)	12.3	NA	NA	48 (24.5)	113 (57.7)
Kato,2023	CheckMate 648	NCT03143153	III	esophageal squamous cell carcinoma	Global	1	Advanced	Niv 3 mg/kg q2w+Ipi 1 mg/kg q6w	130	66 (34-81)	111 (84.7)	NA	NA	NA	120 (91.6)	
								Niv 240 mg q2w+Chemo	121	68 (44-86)	99 (78.6)	NA	NA	NA	109 (86.5)	
								Chemo	135	67 (36-78)	121 (88.3)	NA	NA	NA	120 (87.6)	
NCT02879318	PA7	NCT02879318	II	Pancreatic Adenocarcinoma	MN	1	Metastatic	Dur 1500mg+Tre 75 mg+Chemo	119	64(29-81)	67(56.3)	35	NA	NA	NA	NA
								Chemo	58	65(42-84)	26(42.6)	35	NA	NA	NA	NA
NCT03430063	R2810-ONC-1763	NCT03430063	II	NSCLC	MN	2	Advanced	Cem 350 mg Q3W	8	62.4 (7.91)	6 (75.0)	5.1	NA	NA	NA	NA

								Cem 350 mg q3w + Ipi 50 mg q6w	11	68.5 (8.72)	7(63.6)	NA	NA	NA	NA	NA
								Cem 1050 mg q3w	8	68.1 (12.08)	7(87.5)	8.4	NA	NA	NA	NA
NCT03101566	CA209-9FC	NCT03101566	II	Biliary Tract Cancer	MN	NA	Advanced Unresectable	Niv 360 mg q3w+Gemcitabine+Cisplatin	35	59(20-80)	20(54.1)	NA	NA	NA	NA	NA
								Niv 240mg q2w + Ipi 1 mg/kg q6w	33	61(34-79)	18(47.4)	NA	NA	NA	NA	NA
NCT03167619	DORA	NCT03167619	II	TNBC	United States	NA	Advanced Unresectable	Olaparib	23	52.4 (11.9)	0(0)	21.68	NA	NA	NA	NA
								Olaparib+Dur q4w	22	49.5 (11.7)	0(0)	18.27	NA	NA	NA	NA
NCT01903993	POPLAR	NCT01903993	II	NSCLC	MN	NA	Locally Advanced or Metastatic	Docetaxel	143	61.8 (9.4)	76(53.1)	9.7	NA	NA	NA	NA
								Ate 1200mg q3w	144	61.5 (9.2)	93(64.6)	12.6	NA	NA	NA	NA

NCT02319044	CONDOR	NCT02319044	II	HNSCC	MN	≥2	Recurrent or Metastatic	Dur 20 mg/kg+Tre 1 mg/kg q4w	133	62(26-81)	113(85.0)	12	NA	NA	89(66.9)	24(16.5)
								Dur 10 mg/kg q2w	67	62(23-82)	54(80.6)	12	NA	NA	51(76.1)	7(10.4)
								Tre 10 mg/kg q4w	67	61(42-77)	53(79.1)	12	NA	NA	46(68.7)	6(9.0)
NCT02591615	NA	NCT02591615	II	NSCLC	MN	1	Stage IV	Pem 200mg q3w	67	NA	NA	24	NA	NA	NA	NA
								Carboplatin + Pemetrexed	47	NA	NA	24	NA	NA	NA	NA
								Carboplatin + Paclitaxel	13	NA	NA	24	NA	NA	NA	NA
NCT02985957	CheckMate 650	NCT02985957	II	mCRPC	MN	NA	Metastatic	Niv 3 mg/kg + Ipi 1 mg/kg q4w	73	NA	73(100)	61	NA	NA	NA	NA
								Niv 1 mg/kg + Ipil 3 mg/kg q6w	73	NA	73(100)	61	NA	NA	NA	NA
								Ipi 3 mg/kg q3w	38	NA	38(100)	61	NA	NA	NA	NA
								Cabazitaxel+Prednisone	72	NA	72(100)	61	NA	NA	NA	NA

NCT02551159	KESTREL	NCT02551159	III	HNSCC	MN	1	Recurrent/Metastatic	Dur 1500mg + Tre 75mg q4w	408	60.5 (9.56)	340(82.3)	48	NA	NA	NA	NA
								Dur 1500mg q4w	202	60.2 (10.41)	175(85.8)	48	NA	NA	NA	NA
								Soc	196	60.9 (9.45)	174(84.5)	48	NA	NA	NA	NA
NCT02603432	JAVELIN Bladder 100	NCT02603432	III	UC	MN	1	Locally Advanced Or Metastatic	Ave 10mg/kg q2w+Best Supportive Care (BSC)	344	67.20 (9.52)	266(76.0)	41	NA	NA	NA	NA
								BSC	345	67.7 (9.20)	275(78.6)	41	NA	NA	NA	NA
NCT03553836	KEYNOTE-716	NCT03553836	III	Melanoma	MN	NA	Resected High-risk Stage II	Pem 2 mg/kg q3w	483	59.0 (12.6)	300(61.6)	32.7	NA	NA	NA	NA
								Placebo	486	59.6 (13.3)	289(59.1)	32.7	NA	NA	NA	NA
NCT03033576	S1616	NCT03033576	II	Melanoma	MN	NA	Advanced	Niv 1 mg/kg + Ipi 3mg/kg q3w	68	64(34-90)	15(65.2)	14.5	NA	NA	NA	NA
								Ipi 3mg/kg q3w	23	69(40-91)	46(66.7)	22.1	NA	NA	NA	NA

NCT03875235	TOPAZ-1	NCT03875235	III	Biliary Tract Cancers	MN	1	Advanced	Dur 1500 mg q3w+Gemcitabine+Cisplatin	341	62.2 (10.49)	169(49.6)	27	NA	NA	NA	NA
								Placebo+Gemcitabine+Cisplatin	344	62.6 (10.66)	176(51.2)	27	NA	NA	NA	NA
NCT03459846	BAYOU	NCT03459846	II	UC	MN	1	Unresectable Stage IV	Dur 1500mg q4w+ Olaparib	78	73.4 (10.8)	56(71.8)	31	NA	NA	NA	NA
								Dur 1500mg q4w+Placebo	76	70.2 (10.26)	55(72.4)	31	NA	NA	NA	NA
NCT03517449	KEYNOTE-775	NCT03517449	III	Endometrial Cancer	MN	NA	Advanced	Lenvatinib + Pem 200mg q3w	406	63.2 (9.1)	0(0)	29	NA	NA	NA	NA
								Doxorubicin or Paclitaxel	388	63.8 (9.2)	0(0)	29	NA	NA	NA	NA
NCT03563716	CITYSCAPE	NCT03563716	II	NSCLC	MN	NA	Locally Advanced Or Metastatic	Placebo+Ate 1200mg q3w	68	67.0 (9.9)	48(70.6)	11	NA	NA	NA	NA
								Tiragolumab+Ate 1200mg q3w	67	65.8 (10.4)	39(58.2)	11	NA	NA	NA	NA
NCT02870920	CO26	NCT02870920	II	Colorectal Cancer	MN	NA	Advanced	BSC	61	NA	47(77.0)	24	NA	NA	NA	NA

								Dur 1500mg+Tre 75mg+BSC q4w	118	NA	74(62.2)	24	NA	NA	NA	NA
NCT01843374	NA	NCT01843374	II	Malignant Mesothelioma	MN	≥2	Unresectable	Tre 10mg/kg q4w	380	65.2 (9.24)	283(74.1)	36	NA	NA	NA	NA
								Placebo	189	66.3 (8.8)	151(79.9)	36	NA	NA	NA	NA
NCT01905657	KEYNOTE-010	NCT01905657	II/III	NSCLC	MN	≥2	NA	Pem 2 mg/kg q3w	339	62.1 (9.6)	213(61.7)	24	NA	NA	NA	NA
								Pem 10 mg/kg q3w	343	62.3 (9.7)	213(61.6)	24	NA	NA	NA	NA
								Docetaxel	309	61.6 (9.8)	209(60.9)	24	NA	NA	NA	NA
Klein,2021	CA 209-538	NCT04969887	II	rare gynecological malignancies	MN	≥2	NA	Niv 3mg/kg q2w+Ipi 3 mg/kg and 1 mg/kg q4w	43	59 (20-76)	0(0)	16.8	NA	NA	NA	NA
Mamdani,2021	BTCRC-ESO14-012	NCT02639065	II	GEC	MN	≥2	Locally advanced	Dur 1500mg q4w	37	61 (43-73)	36(97.3)	17.7	NA	NA	NA	NA
O'Malley,2021	C-700-01	NCT03104699	II	cervical cancer	MN	NA	Recurrent and/or metastatic	Balstilimab 3 mg/kg q2w	161	53 (25-81)	0(0)	19.2	NA	NA	NA	NA

Hughes,2021	KEYNOTE-629	NCT03284424	II	CSCC	MN	NA	Recurrent/metastatic	Pem 200mg q3w	159	74.0 (62-82)	119 (74.8)	24	NA	NA	NA	NA
Edenfield,2021	ESR-15-11633	NCT02938793	II	Rare Cancers	United States	≥2	NA	Dur 1500 mg q4w+Tre 75mg q4w	50	62(26-78)	22(44.0)	NA	NA	NA	NA	NA
Bi,2021	CA209-324	NCT02648997	II	Meningioma	United States	≥2	Recurrent	Niv 240mg q2w	25	60 (25-88)	9(36)	21.47	NA	NA	NA	NA
Ardizzoni,2021	MO39171	NCT03285763	III	NSCLC	Global	≥2	Advanced	Ate 1200mg q3w	615	64.0 (24-88)	370 (60.2)	12.6	NA	NA	488 (79.3)	
Rodriguez,2020	NA	NCT02538510	II	HNSCC	United States	≥1	Recurrent Metastatic	Pem 200mg q3w	50	64 (44-78)	39(78)	12.6	NA	NA	18(36)	1(4)
Nomura,2020	NA	NA	II	Melanoma	MN	1	Unresectable or metastatic	Niv 2mg/kg q3w	20	66 (28-84)	13(65)	18	NA	NA	NA	NA
Morgensztern,2020	NA	NCT02250326	II	NSCLC	MN	1	Advanced	Dur 1125mg q3w+nab-paclitaxel	78	63.0 (29-84)	54 (68.4)	12.9	NA	NA	NA	NA
Mei,2020	NA	NCT03346642	II	Lymphoma	China	1	Relapsed/refractory	Cam 200mg q3w+Chemo	27	55.6(37.3-72.4)	5(18.5)	24.8	NA	6(22)	NA	NA

Lan,2020	NA	NCT03816553	II	Cervical Cancer	MN	≥2	Advanced	Cam 200mg q2w+apatinib 250mg qd	45	51 (33-67)	0(0)	11.3	20 (44.4)	40 (88.9)	NA	NA
Dudek,2020	BTCRC-GU 14-003	NCT02348008	II	RCC	MN		Metastatic	Pem 200mg q3w+bevacizumab 15 mg/kg	13	61(42-84)	33 (68.8)	28.3	NA	NA	NA	NA
Vinayak,2019	TOPACIO	NCT02657889	II	TNBC	MN	1	Advanced or Metastatic	Pem 200mg q3w+ niraparib	55	54(32-90)	0(0)	14.8	NA	NA	NA	NA
Okada,2019	MERIT	JapicCTI-163247	II	Malignant Pleural Mesothelioma	MN		Advanced or Metastatic	Niv 240mg q2w	34	68.0 (43-78)	29 (85)	16.8	NA	NA	NA	NA
Habra,2019	NA	NCT02721732	II	ACC	United States	≥2	Advanced	Pem 200mg q3w	16	48 (31-78)	8 (50)	5	NA	NA	NA	NA
Fujimoto,2019	NA	NA	II	NSCLC	MN	≥2	Advanced	Niv	18	71.5 (68.5-76.3)	17 (94)	14.2	NA	NA	NA	NA
Kim,2020	MCC-18684	NCT02829918	II	BTC	MN	2	Advanced Refractory	Niv 240mg q2w	54	65 (28-86)	27 (50)	12.4	NA	NA	NA	NA

Rothschild,2021	SAKK 16/14	NCT02572843	II	NSCLC	MN	≥2	Stage IIIA	Dur 750mg q2w+Chemo	67	61 (41-74)	35 (52)	28.6	NA	NA	36 (54)	28 (42)
McDermott,2021	KEYNOTE-427	NCT02853344	II	RCC	MN	1	Advanced	Pem 200mg q3w	165	62 (22-86)	109 (66.1)	31.5	72 (43.6)	19 (11.5)	NA	NA
Balar,2021	KEYNOTE-057	NCT02625961	II	Bladder cancer	MN	1	BCG-unresponsive	Pem 200mg q3w	101	73 (63-79)	85 (84)	36.4	NA	NA	NA	NA
Antonarakis,2020	KEYNOTE-199	NCT02787005	II	mCRPC	MN	≥2	Treatment-Refractory Metastatic	Pem 200mg q3w	258	68 (48-85)	258(100)	9.5	NA	NA	NA	NA
Bauml,2017	KEYNOTE-055	NCT02255097	II	HNSCC	MN	≥2	Refractory	Pem 200mg q3w	171	61 (33-90)	138 (81)	7	NA	153 (89)	99 (58)	54 (32)
NCT02927301	ML39236	NCT02927301	II	NSCLC	MN	NA	Resectable	Ate 1200mg q3w	181	65.1(9.23)	88(48.6)	42	NA	NA	NA	NA

Abbreviations:MN,multinational;SCLC, small cell lung cancer; RCC, renal cell carcinoma; HNSCC, Head and Neck Squamous Cell Carcinoma; NSCLC, non-small cell lung cancer; UC, urothelial carcinoma; HCC, hepatocellular carcinoma; G/GEC, gastric or gastroesophageal junction cancer; OC, ovarian cancer; TNBC, triple-negative breast cancer; mCRPC, metastatic castration-resistant prostrate cancer; OSCC,oesophageal squamous cell carcinoma; CRC, Colorectal Cancer; NC, Nasopharyngeal Carcinoma; CSCC, cutaneous squamous cell carcinoma; ACC, adrenocortical carcinoma; BTC,biliary tract cancer; Niv, Nivolumab; Ipi, Ipilimumab; TTD, Targeted therapy drug; Chemo, Chemotherapy; Pem, Pembrolizumab; Ate, Atezolizumab; Ave, Avelumab; Dur, Durvalumab; Tre, Tremelimumab;Cam,Camrelizumab;Cem,Cemiplimab;Sin,Sintilimab;Tis,Tislelizumab.Soc,standard of care.

Table S3. Results of individual studies

Study	All-grade PAEs	Grade 3-4 PAEs	Grade 5 PAEs	Treatment
Ascierto,2020	2	1	0	Niv 3mg/kg q2w
	1	0	0	Ipi 10mg/kg q3w
Bellmunt,2021	0	0	0	Placebo

	6	1	1	Ate 1200mg q3w
Emens,2021	0	0	0	Chemo
	1	1	0	Ate 840mg q2w+Chemo
Chen,2020	0	0	0	Placebo
	8	2	0	Niv 3mg/kg q2w
Gutzmer,2020	23	2	0	Ate 840mg q2w+ Cobimetinib + Vemurafenib
	13	0	0	placebo+ Cobimetinib + Vemurafenib
Herbst,2020	8	8	0	Ate 1200mg q3w
	1	1	0	Chemo
Hodi,2018	2	2	0	Niv 3mg/kg q2w
	8	8	0	Niv 1mg/kg q3w+Ipi3 mg/kg q3w
	2	2	0	Ipi 3mg/kg q3w
Janjigian,2021	3	0	3	Niv 360mg q3w or 240mg q2w+Chemo
	1	1	0	Chemo
Jotte,2020	10	3	0	Ate 1200mg q3w+Carboplatin+Paclitaxel
	11	8	1	Ate 1200mg q3w+Carboplatin+Nab-Paclitaxel
	2	2	0	Carboplatin+Nab-Paclitaxel
Kuruvilla,2021	15	8	0	Pem 200mg q3w
	2	2	0	Brentuximab Vedotin
1Wu,2019	22	19	3	Niv 3mg/kg q2w
	0	0	0	Docetaxel
1Motzer,2020	5	2	0	Niv 3mg/kg q2w
	14	3	0	Everolimus
Fradet,2019	8	7	1	Pem 200mg q3w
	0	0	0	Chemo

Antonia,2017	8	2	0	Dur 10mg/kg q2w
	43	6	0	Placebo
1Powles,2020	6	2	0	Dur 1500mg q4w
	12	2	1	Dur 1500mg q4w+Tre 75mg q4w
	1	0	0	Chemo
2Powles,2020	14	0	1	Pem 200mg q3w+Axitinib
	1	0	0	Sunitinib
Powles,2021	4	4	0	Pem 200mg q3w
	0	0	0	Pem 200mg q3w+Chemo
	0	0	0	Chemo
Reardon,2020	4	2	0	Niv 3mg/kg q2w
	0	0	0	Bevacizumab
Rizvi,2020	6	5	1	Dur 20mg/kg q4w
	15	3	7	Dur 20mg/kg q4w+Tre 1mg/kg q4w
	1	0	1	Chemo
Sezer,2021	12	1	1	Cem 350mg q3w
	1	0	0	Chemo
Sun,2021	20	5	2	Pem 200mg q3w
	1	0	1	Chemo
Winer,2021	6	1	2	Pem 200mg q3w
	0	0	0	Chemo
Yang,2021	8	2	0	Cam 200mg q3w+Gemcitabine and Cisplatin
	6	0	0	Gemcitabine and Cisplatin
2Zhou,2021	11	7	0	Sin 200mg q3w+GP
	2	1	0	GP

Ferris,2017	2	1	1	Niv 3mg/kg q2w
	0	0	0	Chemo
Brahmer,2015	6	1	0	Niv 3mg/kg q2w
	1	0	1	Chemo
Borghaei,2015	6	4	0	Niv 3mg/kg q2w
	0	0	0	Chemo
1Robert,2015	1	0	0	Pem 10mg/kg q2w
	5	1	0	Pem 10mg/kg q3w
	1	1	0	Ipi 3 mg/kg q3w
Carbone,2017	7	3	1	Niv 3mg/kg q2w
45	0	0	0	Chemo
Hellmann,2018	9	5	1	Niv 1mg/kg q2w
	28	15	1	Niv 1mg/kg q2w+Ipi 1mg/kg q6w
	3	1	1	Chemo
Eggermont,2015	0	0	0	Ipi 10 mg/kg q3w
	4	4	0	Placebo
Eng,2019	1	1	0	Ate 1200mg q3w
	1	1	0	Ate 840mg q2w+Cobimetinib
	0	0	0	Regorafenib
Long,2019	10	3	0	Pem 200mg q3w
	7	3	0	Pem 200mg q3w+Epacadostat
Reck,2016	1	1	0	Chemo
	2	1	0	Ipi 10mg/kg q3w+Chemo
Socinski,2018	10	5	0	Ate 1200mg q3w+TTD+Chemo
	0	0	0	TTD+chemo

Yang,2019	1	1	0	Dur 10mg/kg q2w+Osimertinib
	0	0	0	Osimertinib
Horn,2018	3	1	0	Ate 1200mg q3w+Chemo
	4	2	0	Chemo
Gogas,2020	0	0	0	Pem 200mg q3w
	3	3	0	Ate 840mg q2w+TTD
Powlers,2018	4	4	0	Ate 1200mg q3w
	2	2	0	Chemo
Barlesi,2018	8	5	3	Ave 10mg/kg q2w
	3	2	1	Docetaxel
Burtness,2019	4	2	1	Pem 200mg q3w
	5	4	1	Pem 200mg q3w+Chemo
	2	2	0	TTD+Chemo
Bajorin,2021	0	0	0	Placebo
	3	1	2	Niv 240mg q2w
Fennell,2021	0	0	0	Placebo
	1	1	0	Niv 240mg q2w
Boyer,2021	32	14	0	Pem 200mg q3w+Ipi 1mg/kg q6w
	13	6	0	Pem 200mg q3w
Govindan,2017	2	2	0	Chemo
	4	1	0	Ipi 10mg/kg q3w+Chemo
Planchard,2020	1	0	0	Dur 10mg/kg q2w
	7	5	0	Dur 20mg/kg q4w+Tre 1mg/kg q4w
	1	0	0	Soc
	0	0	0	Tre 10mg/kg q4w

Weber,2015	2	2	0	Niv 3mg/kg q2w
	0	0	0	Chemo
Ferris,2020	3	3	0	Dur 10mg/kg q2w
	1	1	0	Chemo
	3	3	0	Dur 20mg/kg q4w+Tre 1mg/kg q4w
Pujade-Lauraine,2021	1	1	0	Ave 10mg/kg q2w
	1	1	0	Ave 10mg/kg q2w+PLD
	1	1	0	PLD
Rini,2019	8	8	0	Ate 1200mg q3w+Bevacizumab
	0	0	0	Sunitinib
Beer,2016	0	0	0	Placebo
	6	5	1	Ipi 10mg/kg q3w
Mario,2015	0	0	0	Dacarbazine
	2	2	0	Ipi 10mg/kg q3w+Dacarbazine
2Motzer,2020	16	16	0	Niv 3mg/kg q3w+Ipi 1mg/kg q3w
	1	1	0	Sunitinib
West,2019	10	8	2	Ate 1200mg q3w+carboplatin+nab-paclitaxel
	3	3	0	Chemo
Baas,2021	9	8	1	Niv 3mg/kg q2w+Ipi 1mg/kg q6w
	0	0	0	Chemo
Lee,2021	6	6	0	Ave 10mg/kg q2w+Chemo
	1	1	0	Chemo
Moehler,2021	4	4	0	Ave 10mg/kg q2w
	1	1	0	Chemo
Kato,2019	5	3	2	Niv 240mg q2w

	6	5	1	Chemo
2Choueiri,2021	0	0	0	Placebo
	4	0	0	Pem 200mg q3w
Gettinger,2021	6	5	1	Niv 3mg/kg q2w
	9	7	2	Niv 3mg/kg q2w+Ipi 1mg/kg q6w
Motzer,2021	19	7	0	Pem 200mg q3w+Lenvatinib
	26	1	0	Lenvatinib+Everolimus
	1	0	1	Sunitinib
Monk,2021	0	0	0	Chemotherapy followed by Ave 10mg/kg q2w
	2	2	0	Ave 10mg/kg q3w+Chemo followed by avelumab
	0	0	0	Chemo
NCT02481830	11	11	0	Niv 240mg q2w
	0	0	0	Chemo
NCT03361865	1	1	0	Pem 200mg q3w+TTD
	4	0	0	Pem 200mg q3w
NCT03358472	0	0	0	Pem 200mg q3w
	2	2	0	Pem 200mg q3w+TTD
	0	0	0	Chemo
NCT02279732	1	1	0	Chemo
	0	0	0	Ipi 10mg/kg q3w+Chemo
NCT02611960	3	3	0	Pem 200mg q3w
	0	0	0	Chemo
NCT02864394	14	14	0	Pem 200mg q3w
	2	2	0	Chemo
NCT02576509	6	6	0	Niv 240mg q2w

	0	0	0	Sorafenib
NCT03016312	1	1	0	Ate 1200mg q3w+Enzalutamide
	0	0	0	Enzalutamide
Voss,2022	2	0	0	MEDI0680+durvalumab
	1	1	0	Nivolumab
Loibl,2019	1	0	0	durvalumab
	1	0	0	placebo
McDermott,2018	0	0	0	Sunitinib
	1	0	0	atezolizumab
	0	0	0	atezolizumab + Bev
NCT03563716	1	0	0	Placebo+Ate 1200mg q3w
	0	0	0	Tiragolumab+Ate 1200mg q3w
Xu,2022	10	5	0	sintilimab
	1	0	0	chemo
Taniguchi,2022	9	1	1	nivolumab
	8	2	0	nivolumab + docetaxel
Jung,2022	5	2	0	Pembrolizumab+chemo
	10	4	0	Placebo+chemo
NCT02879318	13	7	0	Gemcitabine + Nab-Paclitaxel + Durvalumab + Tremelimumab
	4	1	0	Gemcitabine Plus Nab-Paclitaxel
NCT03430063	0	0	0	Cemiplimab 350 mg Q3W
	1	1	0	Cemiplimab 350 mg Q3W + Ipilimumab 50 mg Q6W
	0	0	0	Cemiplimab 1050 mg Q3W
NCT03101566	0	0	0	Gemcitabine + Cisplatin + Nivolumab
	1	1	0	Nivolumab + Ipilimumab

NCT03167619	0	0	0	Olaparib
	2	0	0	Olaparib+Durvalumab
NCT01903993	0	0	0	Docetaxel
	1	1	0	Atezolizumab
NCT02319044	1	1	0	Durvalumab+Tremelimumab
	1	1	0	Durvalumab
	0	0	0	Tremelimumab
NCT02591615	2	0	0	Pembrolizumab
	1	0	0	Carboplatin + Pemetrexed
	0	0	0	Carboplatin + Paclitaxel
NCT02985957	1	1	0	Nivolumab 3 mg/kg + Ipilimumab 1 mg/kg Q4W
	4	0	0	Nivolumab 1 mg/kg + Ipilimumab 3 mg/kg Q6W
	2	1	0	Ipilimumab 3 mg/kg
	4	2	0	Cabazitaxel 20 mg/m2 or 25 mg/m2 + Prednisone 10 mg
NCT02551159	9	9	0	Durvalumab + Tremelimumab
	2	2	0	Durvalumab
	2	2	0	Standard of Care (SOC)
NCT02603432	1	1	0	Avelumab + Best Supportive Care (BSC)
	0	0	0	Best Supportive Care
NCT03553836	1	1	0	Pembrolizumab
	0	0	0	Placebo
NCT03033576	4	0	0	Nivolumab + Ipilimumab
	0	0	0	Ipilimumab
NCT03875235	2	2	0	Durvalumab + Gemcitabine + Cisplatin
	2	2	0	Placebo + Gemcitabine + Cisplatin

NCT03517449	3	3	0	Lenvatinib + Pembrolizumab
	0	0	0	Doxorubicin or Paclitaxel
NCT02870920	0	0	0	Best Supportive Care
	2	2	0	Durvalumab + Tremelimumab + Best Supportive Care
NCT01843374	2	0	0	TREMELIMUMAB
	0	0	0	PLACEBO
NCT01905657	24	15	0	Pembrolizumab 2 mg/kg
	18	10	0	Pembrolizumab 10 mg/kg
	3	2	0	Docetaxel
1Choueiri,2021	13	3	0	Niv 240mg q2w+Cabozantinib
	0	0	0	Sunitinib
Cortes,2020	0	0	0	Chemo
	20	6	0	Pem 200mg q3w+Chemo
Finn,2020	1	0	0	Placebo
	14	4	0	Pem 200mg q3w
Fuchs,2020	10	2	0	Pem 200mg q3w
	0	0	0	Paclitaxel
Huang,2020	17	0	1	Cam 200mg q2w
	0	0	0	Chemo
Miles,2021	19	2	1	Ate 840mg q2w+Chemo
	3	0	0	Chemo
Mittendorf,2020	3	1	0	Ate 840mg q2w+Chemo
	2	0	0	Chemo
Moore,2021	12	0	0	Ate 1200mg q3w
	5	1	0	Chemo+Bevacizumab

Reck,2021	0	0	0	Chemo
	21	10	1	Niv 360mg q2w+Ipi 1mg/kg q6w+Chemo
Rudin,2020	9	0	0	Pem 200mg q3w
	5	0	0	Etoposide
Shitara,2020	9	8	1	Pem 200mg q3w
	8	8	0	Pem 200mg q3w+Chemo
	1	0	1	Chemo
Sugawara,2021	5	2	0	Placebo
	27	6	1	Niv 360mg q3w
1Zhou,2021	6	0	0	Sin 200mg q3w+GP
	0	0	0	GP
Reck,2019	16	3	1	Pem 200mg q3w
	2	1	0	Chemo
Fehrenbacher,2018	13	4	0	Ate 1200mg q3w
	0	0	0	Docetaxel
Paz-Ares,2018	9	2	1	Pem 200mg q3w+Chemo
	25	6	1	Chemo
Mok,2019	53	21	1	Pem 200mg q3w
	3	1	0	Chemo
Paz-Ares,2019	0	0	0	Dur 1500mg q4w+Chemo
	2	0	1	Chemo
Gandhi,2018	29	8	3	Pem 200mg q3w+Chemo
	9	3	1	Chemo
Cohen,2019	6	3	0	Placebo

	13	3	0	Pem 200mg q3w
Eggermont,2018	3	0	0	Placebo
	21	4	0	Pem 200mg q3w
Kojima,2020	15	5	2	Pem 200mg q3w
	2	1	0	Chemo
Finns,2020	4	0	2	Ate 1200mg q3w
	0	0	0	Sorafenib
Owonikoko,2021	5	0	1	Placebo
	14	4	0	Niv 240mg q2w
	27	8	0	Niv 1mg/kg q3w+ Ipi 3mg/kg q3w
2Wu,2020	13	2	1	Pem 200mg q3w
	0	0	0	Chemo
Usmani,2019	0	0	0	Lenalidomide and Dexamethasone
	1	0	0	Pem 200mg q3w+Lenalidomide and Dexamethasone
Hamanishi,2021	3	0	0	Niv 240 mg q2w
	4	0	0	GEM or PLD
Wang,2021	7	2	0	Tis 200mg q3w+PC
	1	0	0	PC
	7	2	0	Tis 200mg q3w+nab-PC
2Robert,2015	3	0	0	Niv 3mg/kg q2w
	0	0	0	Chemo
Schmid,2020	13	3	0	Pem 200mg q3w+Chemo
	6	1	0	Chemo
Yang,2020	11	2	0	Sin 200mg q3w+Chemo

	3	1	0	Chemo
Shitara,2018	10	1	1	Pem 200mg q3w
	0	0	0	Paclitaxel
Mateos,2019	0	0	0	Chemo
	5	1	0	Tis 200mg q3w+PC
Lu,2021	20	10	3	Tislelizumab+Chemo
	1	0	1	Chemo
Scherpereel,2019	1	0	0	Niv 3mg/kg q2w
	2	1	0	Niv 3mg/kg q2w+Ipi 1mg/kg q6w
Zhou,2023	31	18	0	Tislelizumab 200 mg q3w
	0	0	0	placebo+ Cobimetinib + Vemurafenib
Johnson,2023	12	3	0	Niv 3mg/kg q2w
	10	4	0	Dur 1500mg+Chemo
	2	2	0	Chemo
Forde,2022	2	0	0	Niv 360mg q3w+Chemo
	1	1	0	Niv 360mg q3w+Chemo
Jr,2023	24	4	3	Niv 3mg/kg q2w
	1	0	0	Chemo
Herbst,2022	11	0	2	Dur 1500mg q4w
	11	0	1	Dur 1500mg q4w+Oleclumab
	10	1	0	Dur 1500mg q4w+Monalizumab
Diaz,2022	6	0	0	Pem 200 mg q3w
	2	0	0	Chemo
Rossevold,2022	1	0	0	Chemo
	4	2	0	Ate 840 mg q2w+Chemo

Park,2022	3	0	1	Dur 20 mg/kg q4w
	1	0	0	Placebo
Cheng,2022	5	0	0	Ate 1200mg+Bevacizumab q3w
	0	0	0	Sorafenib
Leighl,2022	9	3	0	Dur 1500 mg + Tre 75mg + Chemo q3w
	9	4	0	Pem 200 mg q3w
2Cheng,2022	1	1	0	Serplulimab 4.5mg/kg q3w
	2	0	0	Placebo
Kato,2023	16	4	0	Niv 3 mg/kg q2w+Ipi 1 mg/kg q6w
	7	0	0	Chemo
	0	0	0	Placebo
NCT03459846	1	1	0	Niv 1mg/kg+Ipi 3mg/kg q3w
	0	0	0	Dur 1500mg q4w+Placebo
Peters,2021	22	7	0	Niv 1mg/kg+Ipi 3mg/kg q3w
	4	1	0	Placebo
Klein,2021	2	2	0	Niv 3mg/kg q2w+Ipi 3 mg/kg and 1 mg/kg q4w
Mamdani,2021	1	0	0	Dur 1500mg q4w
O'Malley,2021	5	2	0	Balstilimab 3 mg/kg q2w
Hughes,2021	6	5	0	Pem 200mg q3w
Edenfield,2021	1	0	1	Dur 1500 mg q4w+Tre 75mg q4w
Bi,2021	2	1	0	Niv 240mg q2w
Ardizzoni,2021	48	16	9	Ate 1200mg q3w
Rodriguez,2020	1	0	1	Pem 200mg q3w
Nomura,2020	1	1	0	Niv 2mg/kg q3w
Morgensztern,2020	2	0	1	Dur 1125mg q3w+nab-paclitaxel

Mei,2020	2	1	0	Cam 200mg q3w+Chemo
Lan,2020	1	0	0	Cam 200mg q2w+apatinib 250mg qd
Dudek,2020	2	0	0	Pem 200mg q3w+bevacizumab 15 mg/kg
Vinayak,2019	1	1	0	Pem 200mg q3w+ niraparib
Okada,2019	2	0	0	Niv 240mg q2w
Habra,2019	1	0	0	Pem 200mg q3w
Fujimoto,2019	2	2	0	Niv
Kim,2020	1	1	0	Niv 240mg q2w
Rothschild,2021	2	1	0	Dur 750mg q2w+Chemo
McDermott,2021	3	3	0	Pem 200mg q3w
Balar,2021	3	3	0	Pem 200mg q3w
Antonarakis,2020	6	5	1	Pem 200mg q3w
Bauml,2017	6	4	1	Pem 200mg q3w
NCT02927301	8	0	0	Ate 1200mg q3w

Abbreviations: PAEs, pulmonary adverse events; Niv, Nivolumab; Ipi, Ipilimumab; TTD, Targeted therapy drug; Chemo, Chemotherapy; Pem, Pembrolizumab; Ate, Atezolizumab; Ave, Avelumab; Dur, Durvalumab; Tre, Tremelimumab; Cam,Camrelizumab; Cem,Cemiplimab;Sin,Sintilimab;Tis,Tislelizumab.Soc,standard of care.

Table S4. Fatal Pulmonary Adverse Events Outcome: League Table

Chemo																	
-9.48<br>(-38.26, 0.93)	CTLA4																
31.06<br>(-14.05, 103.87)	46.31<br>(-5.8, 108.96)	CTLA4+Chemo															
**-1.55<br>(-2.75, -0.59)**	7.99<br>(-2.39, 36.51)	-32.7<br>(-105.48, 12.67)	PD1														
-0.64<br>(-1.88, 0.47)	8.91<br>(-1.53, 37.65)	-31.72<br>(-104.45, 13.34)	0.9<br>(-0.48, 2.41)	PD1+Chemo													
-1.3<br>(-3.23, 0.49)	8.25<br>(-2.43, 36.8)	-32.31<br>(-105.2, 12.7)	0.26<br>(-1.34, 2.04)	-0.64<br>(-2.85, 1.45)	PD1+CTLA4												
**-19.91<br>(-57.08, -0.13)**	-7.7<br>(-45.19, 27.33)	**-51.23<br>(-130.44, -3.81)**	-18.3<br>(-55.65, 1.5)	-19.21<br>(-56.57, 0.63)	-18.54<br>(-55.7, 1.31)	PD1+CTLA4+Chemo											
10.81<br>(-44.14, 75.61)	21.15<br>(-25.68, 91.47)	-23.83<br>(-107.8, 70.61)	12.48<br>(-42.29, 77.35)	11.59<br>(-43.65, 76.23)	12.13<br>(-42.29, 77.03)	32.68<br>(-26.36, 103.79)	PD1+PDL1										
**23.62<br>(3.47, 68.5)**	**37.15<br>(7.9, 79.27)**	1.16<br>(-88.68, 47.95)	**25.21<br>(5, 70.14)**	**24.33<br>(4.23, 69.21)**	**24.96<br>(4.8, 69.95)**	**43.23<br>(10.91, 102.43)**	18.57<br>(-63.13, 77.38)	PD1+TTD									
-1.11<br>(-3.34, 0.72)	8.47<br>(-2.47, 36.65)	-32.02<br>(-105.08, 13.93)	0.44<br>(-1.85, 2.53)	-0.49<br>(-2.93, 1.65)	0.17<br>(-2.62, 2.64)	18.78<br>(-1.35, 56)	-12<br>(-76.9, 42.81)	**-24.87<br>(-69.74, -4.66)**	PDL1								
-0.96<br>(-3.73, 1.13)	8.43<br>(-2.26, 36.76)	-31.93<br>(-104.78, 12.12)	0.62<br>(-2.24, 2.96)	-0.3<br>(-3.3, 2.03)	0.37<br>(-2.95, 3.13)	18.67<br>(-1.37, 55.92)	-11.86<br>(-76.62, 43.08)	**-24.63<br>(-69.66, -4.34)**	0.21<br>(-3.2, 3.13)	PDL1+Chemo							
**-3.11<br>(-5.91, -1.21)**	6.4<br>(-4.66, 34.44)	-34.13<br>(-107.27, 11.73)	-1.54<br>(-4.37, 0.6)	**-2.5<br>(-5.42, -0.21)**	-1.84<br>(-5.16, 0.74)	16.67<br>(-3.59, 53.95)	-14.24<br>(-78.92, 40.81)	**-26.95<br>(-71.02, -6.49)**	**-1.97<br>(-4.35, -0.2)**	-2.23<br>(-5.66, 1.27)	PDL1+CTLA4						
31.25<br>(-13.17, 118.25)	46.07<br>(-3.14, 132.87)	0.95<br>(-73.76, 82.03)	32.96<br>(-11.65, 119.94)	31.92<br>(-12.6, 119)	32.85<br>(-11.88, 119.4)	55.67<br>(-1.12, 133.4)	21.75<br>(-47.71, 119.54)	5.2<br>(-57.32, 96.02)	32.62<br>(-12.06, 119.72)	32.33<br>(-12.06, 118.72)	34.66<br>(-9.94, 121.49)	PDL1+CTLA4+Chemo					
1.28<br>(-1.8, 5.68)	**11.39<br>(0.01, 39.34)**	-28.44<br>(-102.83, 16.21)	2.85<br>(-0.32, 7.4)	1.97<br>(-1.36, 6.42)	2.66<br>(-0.91, 7.34)	**21.27<br>(1.44, 58.69)**	-9.42<br>(-75.05, 44.51)	**-22.46<br>(-66.77, -2.75)**	**2.42<br>(0.09, 6.31)**	2.22<br>(-1.33, 7.61)	**4.47<br>(1.47, 9.06)**	-30.59<br>(-116.8, 14.71)	PDL1+TTD				
7<br>(-95.09, 91.96)	17.44<br>(-83.5, 114.05)	-17.35<br>(-179.2, 69.13)	8.62<br>(-93.66, 93.16)	7.52<br>(-94.59, 91.86)	8.44<br>(-94.02, 92.75)	29.99<br>(-70.43, 121.32)	-8.1<br>(-114.51, 105.18)	-21.61<br>(-106.88, 57.99)	8.2<br>(-94.12, 93.6)	7.95<br>(-95.07, 93.18)	10.29<br>(-92.49, 95.57)	-26.55<br>(-159.73, 89.26)	5.65<br>(-96.88, 89.95)	PDL1TTD+Chemo			
0.54<br>(-1.87, 4.23)	10.25<br>(-0.25, 38.53)	-30.04<br>(-103.19, 15.42)	2.1<br>(-0.17, 5.88)	1.21<br>(-1.4, 5.04)	1.87<br>(-0.9, 5.83)	**20.43<br>(0.58, 58.07)**	-10.15<br>(-74.6, 44.09)	**-23.29<br>(-68.44, -2.64)**	1.74<br>(-0.8, 5.43)	1.67<br>(-1.78, 5.81)	**3.78<br>(0.86, 7.93)**	-31.07<br>(-116.98, 13.68)	-0.73<br>(-5.21, 3.54)	-6.65<br>(-91.45, 95.62)	Placebo		
**23.9<br>(3.7, 68.65)**	**36.91<br>(8.4, 79.4)**	1.45<br>(-88.84, 47.53)	**25.59<br>(5.51, 70.32)**	**24.55<br>(4.37, 69.29)**	**25.38<br>(5.14, 69.95)**	**43.37<br>(11.37, 102.35)**	18.82<br>(-62.59, 77.63)	-0.03<br>(-4.11, 3.95)	**25.12<br>(5.24, 69.79)**	**24.98<br>(4.67, 69.77)**	**27.26<br>(7.03, 71.44)**	-6.14<br>(-95.86, 57.38)	**22.83<br>(3.22, 67.04)**	21.66<br>(-57.8, 106.75)	**23.68<br>(2.87, 68.3)**	TTD	
**35.59<br>(1.5, 105.82)**	**48.25<br>(6.86, 119.87)**	9.88<br>(-76.98, 87.66)	**37.19<br>(3.03, 107.44)**	**36.22<br>(2.1, 106.27)**	**37.04<br>(2.65, 107.13)**	**58.01<br>(11.04, 142.25)**	23.9<br>(-46.08, 117.67)	14.07<br>(-40.5, 70.5)	**36.86<br>(2.82, 106.93)**	**36.73<br>(2.51, 107.02)**	**38.98<br>(4.71, 109.28)**	1.59<br>(-91.35, 93.99)	34.39<br>(-0.04, 104.25)	28.79<br>(-14.51, 118.68)	**34.74<br>(0.76, 104.85)**	13.74<br>(-40.69, 70.39)	TTD+Chemo

Network estimates of treatment comparisons for fatal PAEs. The summary estimates are log odds ratios (LORs) and 95% confidence intervals.The column-defining treatment is compared to the row-defining treatment, and LORs < 0 favor the column-defining treatment. significant results are in bold.

Abbreviations: PAEs, pulmonary adverse events;TTD, Targeted therapy drug; Chemo, Chemotherapy; PD-1, programmed cell death 1; PD-L1, programmed cell death ligand 1; CTLA4, cytotoxic T-lymphocyte antigen 4.

Table S5. All-grade Treatment-related Pulmonary Adverse Events in Tumors of Different Systems Outcome: League Table

(A) respiratory system cancer

Chemo													
**37.82<br>(0.17, 123.26)**	CTLA4												
-0.32<br>(-2.07, 1.39)	**-38.2<br>(-123.42, -0.45)**	CTLA4+Chemo											
**-2.28<br>(-2.93, -1.73)**	**-40.11<br>(-125.62, -2.46)**	**-1.96<br>(-3.81, -0.14)**	PD1										
**-1.04<br>(-1.68, -0.48)**	**-38.88<br>(-124.37, -1.22)**	-0.72<br>(-2.55, 1.11)	**1.24<br>(0.5, 2)**	PD1+Chemo									
**-2.71<br>(-3.73, -1.85)**	**-40.56<br>(-126.08, -2.89)**	**-2.4<br>(-4.4, -0.45)**	-0.44<br>(-1.3, 0.38)	**-1.68<br>(-2.68, -0.73)**	PD1+CTLA4								
**-21.51<br>(-78.44, -4.53)**	**-64.04<br>(-165.77, -15.74)**	**-21.19<br>(-78.26, -4.09)**	**-19.22<br>(-76.18, -2.21)**	**-20.46<br>(-77.44, -3.48)**	**-18.79<br>(-75.78, -1.74)**	PD1+CTLA4+Chemo							
**-1.87<br>(-2.9, -0.95)**	**-39.71<br>(-125.19, -2.06)**	-1.55<br>(-3.56, 0.42)	0.41<br>(-0.67, 1.48)	-0.83<br>(-1.96, 0.27)	0.85<br>(-0.42, 2.14)	**19.65<br>(2.65, 76.58)**	PDL1						
**-0.97<br>(-1.87, -0.06)**	**-38.78<br>(-124.26, -1.14)**	-0.65<br>(-2.58, 1.33)	**1.31<br>(0.26, 2.46)**	0.07<br>(-0.99, 1.21)	**1.75<br>(0.51, 3.14)**	**20.56<br>(3.53, 77.48)**	0.9<br>(-0.36, 2.27)	PDL1+Chemo					
**-2.63<br>(-3.84, -1.53)**	**-40.44<br>(-125.96, -2.83)**	**-2.31<br>(-4.42, -0.25)**	-0.35<br>(-1.63, 0.91)	**-1.59<br>(-2.89, -0.32)**	0.09<br>(-1.35, 1.54)	**18.88<br>(1.88, 75.86)**	-0.76<br>(-1.95, 0.41)	**-1.66<br>(-3.11, -0.32)**	PDL1+CTLA4				
**-1.96<br>(-3.35, -0.61)**	**-39.79<br>(-125.19, -2.15)**	-1.63<br>(-3.85, 0.56)	0.32<br>(-1.14, 1.82)	-0.92<br>(-2.4, 0.58)	0.76<br>(-0.84, 2.45)	**19.56<br>(2.54, 76.56)**	-0.08<br>(-1.63, 1.49)	-0.99<br>(-2.45, 0.4)	0.68<br>(-0.71, 2.1)	PDL1+CTLA4+Chemo			
**-1.91<br>(-3.55, -0.37)**	**-39.76<br>(-125.2, -2.07)**	-1.59<br>(-3.98, 0.72)	0.36<br>(-1.28, 2.02)	-0.88<br>(-2.56, 0.8)	0.8<br>(-0.96, 2.61)	**19.59<br>(2.53, 76.53)**	-0.04<br>(-1.3, 1.22)	-0.94<br>(-2.81, 0.81)	0.72<br>(-1, 2.45)	0.04<br>(-1.98, 2.02)	PDL1+TTD		
**-1.13<br>(-2.32, -0.07)**	**-38.96<br>(-124.43, -1.31)**	-0.81<br>(-2.92, 1.22)	**1.15<br>(0.1, 2.16)**	-0.09<br>(-1.32, 1.08)	**1.58<br>(0.38, 2.78)**	**20.36<br>(3.33, 77.34)**	0.74<br>(-0.51, 1.93)	-0.16<br>(-1.69, 1.21)	1.5<br>(-0.02, 2.97)	0.83<br>(-0.95, 2.5)	0.79<br>(-1.01, 2.5)	Placebo	
**42.1<br>(0.27, 142.3)**	4.84<br>(-95.07, 108.76)	**42.4<br>(0.54, 142.48)**	**44.4<br>(2.55, 144.67)**	**43.15<br>(1.3, 143.45)**	**44.85<br>(2.97, 145.1)**	**69.46<br>(14.11, 171.58)**	**44.01<br>(2.17, 144.29)**	**43.09<br>(1.25, 143.24)**	**44.76<br>(2.92, 145)**	**44.08<br>(2.19, 144.36)**	**44.05<br>(2.24, 144.35)**	**43.26<br>(1.4, 143.46)**	TTD

(B) urogenital system cancer

Chemo												
-0.32<br>(-3.69, 3.11)	CTLA4											
-1.3<br>(-4.32, 1.17)	-1<br>(-5.47, 2.96)	PD1										
**34.98<br>(0.56, 109.39)**	**35.28<br>(0.61, 109.71)**	**36.33<br>(1.99, 110.84)**	PD1+Chemo									
-0.34<br>(-3.38, 2.78)	-0.03<br>(-3.48, 3.47)	0.97<br>(-2.52, 5)	**-35.32<br>(-109.72, -0.72)**	PD1+CTLA4								
-1.6<br>(-8.02, 4.15)	-1.29<br>(-8.44, 5.25)	-0.27<br>(-5.88, 4.99)	**-36.71<br>(-111.29, -1.51)**	-1.26<br>(-8.19, 4.97)	PD1+PDL1							
-2.25<br>(-6.2, 1.43)	-1.94<br>(-6.89, 2.71)	-0.94<br>(-4.24, 2.52)	**-37.29<br>(-111.73, -2.66)**	-1.91<br>(-6.27, 2.12)	-0.66<br>(-6.85, 5.95)	PD1+TTD						
-1.76<br>(-4.7, 0.88)	-1.45<br>(-5.95, 2.74)	-0.45<br>(-4.09, 3.31)	**-36.77<br>(-111.1, -2.18)**	-1.42<br>(-5.63, 2.43)	-0.16<br>(-6.55, 6.59)	0.5<br>(-3.94, 4.85)	PDL1					
-1.26<br>(-4.64, 1.79)	-0.95<br>(-5.77, 3.56)	0.05<br>(-4.03, 4.29)	**-36.3<br>(-110.68, -1.57)**	-0.92<br>(-5.53, 3.32)	0.33<br>(-6.31, 7.34)	0.99<br>(-3.95, 5.88)	0.5<br>(-3.29, 4.29)	PDL1+Chemo				
-2.61<br>(-7.23, 1.73)	-2.31<br>(-8.02, 3.16)	-1.31<br>(-6.36, 3.94)	**-37.63<br>(-111.95, -2.71)**	-2.28<br>(-7.82, 2.93)	-1.02<br>(-8.29, 6.65)	-0.36<br>(-6.1, 5.36)	-0.86<br>(-5.18, 3.51)	-1.35<br>(-6.63, 3.97)	PDL1+CTLA4			
-3.77<br>(-9.18, 0.58)	-3.47<br>(-9.76, 1.82)	-2.44<br>(-7.94, 2.27)	**-38.96<br>(-113.58, -3.97)**	-3.43<br>(-9.42, 1.43)	-2.19<br>(-9.75, 5.13)	-1.47<br>(-7.3, 3.2)	-2<br>(-7.02, 2.21)	**-2.51<br>(-8.48, 2.74)**	-1.17<br>(-7.65, 4.63)	PDL1+TTD		
**40.75<br>(5.37, 109.44)**	**41.12<br>(5.68, 109.58)**	**42.14<br>(6.63, 110.88)**	5.52<br>(-73.99, 84.95)	**41.11<br>(5.64, 109.69)**	**42.47<br>(6.33, 111.19)**	**43.01<br>(7.38, 111.8)**	**42.53<br>(7.08, 111.19)**	**42.04<br>(6.47, 110.81)**	**43.41<br>(7.61, 111.99)**	**44.59<br>(8.74, 113.24)**	Placebo	
0.46<br>(-2.81, 3.95)	0.78<br>(-3.57, 5.28)	1.78<br>(-1.05, 5.22)	-34.49<br>(-108.95, 0.09)	0.8<br>(-2.79, 4.54)	2.07<br>(-3.8, 8.73)	**2.72<br>(0.51, 5.4)**	2.22<br>(-1.52, 6.52)	1.73<br>(-2.63, 6.57)	3.08<br>(-2.13, 8.75)	**4.17<br>(0.19, 9.91)**	**-40.23<br>(-108.99, -4.72)**	TTD

(C) skin cancer

Chemo								
**-11.38 (-37.48, -0.96)**	CTLA4							
**-14.04 (-51.2, -0.74)**	-2.57 (-40.7, 26.32)	CTLA4+Chemo						
**-11.88 (-38, -1.62)**	-0.52 (-1.96, 0.95)	2.01 (-26.68, 40.25)	PD1					
**-13.45 (-39.45, -2.84)**	**-1.97 (-4.34, -0.11)**	0.54 (-28.33, 38.77)	-1.46 (-3.91, 0.45)	PD1+CTLA4				
**-11.56 (-37.65, -0.93)**	-0.15 (-2.84, 2.62)	2.39 (-26.46, 40.76)	0.38 (-1.95, 2.71)	1.83 (-1.06, 5.29)	PD1+TTD			
**-29.17 (-62.78, -7.32)**	**-16.04 (-41.72, -1.67)**	**-14.14 (-52.55, 28.76)**	**-15.49 (-41.12, -1.28)**	-14.02 (-39.73, 0.5)	**-15.87 (-41.6, -1.41)**	PDL1+TTD		
-9.14 (-35.53, 1.43)	**2.11 (0.15, 5.01)**	4.82 (-24.03, 43.17)	**2.63 (0.93, 5.25)**	**4.1 (1.71, 7.84)**	2.24 (-0.45, 5.9)	**18.27 (3.76, 43.92)**	Placebo	
**-28.42 (-62.06, -6.42)**	**-15.25 (-41.01, -0.69)**	-13.38 (-51.79, 29.59)	**-14.72 (-40.39, -0.29)**	-13.23 (-39.03, 1.48)	**-15.09 (-40.84, -0.43)**	0.79 (-1.46, 3.03)	**-17.48 (-43.18, -2.78)**	TTD

(D) head and neck cancer

Chemo									
10.4 (-2.12, 40.94)	CTLA4								
-1.36 (-4, 0.27)	-11.88 (-42.38, 0.6)	PD1							
-0.89 (-3.14, 0.72)	-11.36 (-41.85, 1.19)	0.46 (-1.28, 2.67)	PD1+Chemo						
**-13.53 (-44.51, -1.97)**	**-26.76 (-68.6, -4.51)**	**-12.04 (-43.04, -0.61)**	**-12.61 (-43.58, -0.98)**	PD1+TTD					
-1.06 (-3.95, 1.08)	-11.52 (-42.02, 0.6)	0.31 (-2.2, 3.06)	-0.16 (-3.1, 2.61)	**12.45 (0.62, 43.32)**	PDL1				
-2.12 (-6, 0.63)	-12.7 (-43.27, 0.43)	-0.76 (-4.86, 3.19)	-1.23 (-5.35, 2.4)	11.36 (-0.96, 42.26)	-1.08 (-5.45, 2.98)	PDL1+Chemo			
-1.36 (-4.16, 0.75)	-11.79 (-42.29, 0.31)	0.02 (-2.4, 2.78)	-0.46 (-3.3, 2.32)	**12.14 (0.37, 43.09)**	-0.29 (-1.85, 1.32)	0.79 (-3.21, 5.11)	PDL1+CTLA4		
-0.6 (-3.48, 1.45)	-11.08 (-41.57, 1.29)	0.76 (-1.11, 2.85)	0.31 (-2.4, 2.73)	**12.84 (1.19, 43.85)**	0.45 (-1.84, 2.71)	1.53 (-2.55, 5.84)	0.74 (-1.51, 2.91)	Placebo	
-0.24 (-3.44, 2.57)	-10.66 (-41.13, 2.12)	1.15 (-1.18, 3.96)	0.67 (-1.86, 3.33)	**13.32 (1.48, 44.33)**	0.86 (-2.57, 4.43)	1.97 (-2.4, 6.56)	1.16 (-2.29, 4.65)	0.38 (-2.63, 3.67)	TTD+Chemo

(E) digestive system cancer

Chemo									
**-2.39 (-4.02, -1.33)**	PD1								
**-2.6 (-4.69, -0.98)**	-0.21 (-2.19, 1.88)	PD1+Chemo							
**-4.09 (-7.94, -1.57)**	-1.69 (-5.32, 1.15)	-1.47 (-4.7, 0.8)	PD1+CTLA4						
**-24.33 (-75.52, -1.89)**	-21.91 (-73.01, 0.63)	-21.72 (-72.78, 0.93)	-20.24 (-71.18, 2.78)	PD1+TTD					
-1.25 (-4.44, 1.71)	1.16 (-1.92, 4.44)	1.37 (-2.09, 4.99)	2.86 (-1.07, 7.77)	**23.12 (0.3, 74.17)**	PDL1				
**-38.69 (-122.03, -1.44)**	-36.22 (-119.45, 1.02)	-36.03 (-119.28, 1.27)	-34.42 (-117.66, 2.95)	-13.4 (-95.26, 47.63)	**-37.44 (-120.73, -0.05)**	PDL1+CTLA4			
-0.54 (-3.74, 2.62)	1.84 (-1.32, 5.58)	2.06 (-1.41, 5.94)	3.53 (-0.32, 8.71)	**23.83 (0.97, 75.01)**	0.71 (-3.64, 5.17)	**38.14 (0.69, 121.51)**	PDL1+CTLA4+Chemo		
0.01 (-3.73, 3.57)	2.42 (-1.15, 6.24)	2.63 (-1.32, 6.75)	4.12 (-0.2, 9.44)	**24.4 (1.4, 75.48)**	1.27 (-1.31, 3.79)	**38.69 (1.29, 122.01)**	0.56 (-4.34, 5.34)	PDL1+TTD	
0.33 (-2.4, 3.45)	**2.74 (0.36, 5.99)**	2.96 (-0.09, 6.64)	**4.48 (0.86, 9.4)**	**24.76 (1.95, 75.83)**	1.6 (-1.29, 4.92)	**39.06 (1.97, 122.22)**	0.91 (-3.3, 5.31)	0.33 (-2.83, 3.96)	Placebo
**29.12 (3.51, 95.23)**	**31.57 (5.94, 97.68)**	**31.77 (6.07, 97.97)**	**33.38 (7.46, 99.62)**	**57.64 (14.57, 140.61)**	**30.37 (4.93, 96.54)**	**74.81 (14.88, 175.6)**	**29.65 (3.77, 95.82)**	**29.08 (3.73, 95.23)**	**28.76 (3.14, 94.89)**

Table S6. Grade 3-4 Treatment-related Pulmonary Adverse Events in Tumors of Different Systems Outcome: League Table

(A) respiratory system cancer

Chemo													
24.28 (-0.98, 92.76)	CTLA4												
0.88 (-1.09, 3.24)	-23.4 (-91.77, 2.06)	CTLA4+Chemo											
**-2.34 (-3.24, -1.65)**	**-26.65 (-95.08, -1.38)**	**-3.22 (-5.79, -1.14)**	PD1										
**-1 (-1.9, -0.25)**	**-25.28 (-93.78, -0.03)**	-1.88 (-4.42, 0.21)	**1.33 (0.38, 2.37)**	PD1+Chemo									
**-2.85 (-4.17, -1.88)**	**-27.2 (-95.62, -1.88)**	**-3.75 (-6.49, -1.56)**	-0.52 (-1.52, 0.34)	**-1.85 (-3.13, -0.79)**	PD1+CTLA4								
**-19.19 (-73.23, -3.09)**	**-49.95 (-120.74, -9.77)**	**-20.14 (-74.14, -3.77)**	**-16.82 (-70.92, -0.7)**	**-18.17 (-72.2, -2.03)**	**-16.3 (-70.31, -0.13)**	PD1+CTLA4+Chemo							
**-1.79 (-3.12, -0.72)**	**-26.09 (-94.55, -0.87)**	**-2.68 (-5.39, -0.45)**	0.55 (-0.81, 1.86)	-0.79 (-2.26, 0.56)	1.07 (-0.43, 2.64)	**17.35 (1.15, 71.66)**	PDL1						
-0.69 (-1.73, 0.33)	-24.96 (-93.48, 0.33)	-1.57 (-4.13, 0.65)	**1.64 (0.45, 3.05)**	0.31 (-0.94, 1.7)	**2.17 (0.79, 3.86)**	**18.52 (2.34, 72.53)**	1.1 (-0.36, 2.77)	PDL1+Chemo					
**-2.29 (-4.14, -0.92)**	**-26.63 (-95.11, -1.45)**	**-3.19 (-6.2, -0.79)**	0.04 (-1.76, 1.59)	-1.29 (-3.2, 0.28)	0.57 (-1.33, 2.3)	**16.8 (0.57, 71.07)**	-0.5 (-2.1, 0.84)	-1.61 (-3.67, 0.03)	PDL1+CTLA4				
-1.09 (-2.84, 0.52)	**-25.36 (-93.91, -0.1)**	-1.98 (-4.92, 0.57)	1.25 (-0.53, 3.09)	-0.08 (-1.94, 1.76)	1.77 (-0.11, 3.82)	**18.07 (1.82, 72.13)**	0.7 (-1.13, 2.6)	-0.39 (-2.21, 1.26)	1.21 (-0.4, 3.12)	PDL1+CTLA4+Chemo			
**-15.22 (-50.08, -1.37)**	**-43.27 (-113.43, -7.55)**	**-16.17 (-51.07, -2)**	-12.86 (-47.68, 1.04)	**-14.2 (-49.06, -0.31)**	-12.33 (-47.16, 1.61)	3.63 (-35.52, 61.44)	-13.39 (-48.21, 0.36)	**-14.54 (-49.39, -0.61)**	-12.85 (-47.64, 1.02)	**-14.1 (-48.92, -0.11)**	PDL1+TTD		
-0.68 (-2.21, 1)	-24.96 (-93.49, 0.36)	-1.54 (-4.39, 1.04)	**1.66 (0.28, 3.36)**	0.33 (-1.29, 2.17)	**2.19 (0.67, 4.12)**	**18.54 (2.28, 72.64)**	1.12 (-0.44, 2.95)	0.02 (-1.84, 1.94)	1.64 (-0.24, 3.97)	0.44 (-1.74, 2.75)	**14.58 (0.62, 49.38)**	Placebo	
10.04 (-28.92, 75.23)	**-17 (-84.81, 58.22)**	9.07 (-30.03, 74.26)	12.39 (-26.54, 77.6)	11.05 (-27.87, 76.26)	12.93 (-25.99, 78.15)	32.99 (-11.06, 110.78)	11.88 (-27.04, 77.06)	10.69 (-28.25, 75.92)	12.4 (-26.49, 77.62)	11.15 (-27.83, 76.25)	**26.16 (1.09, 91.39)**	10.65 (-28.22, 75.91)	TTD

(B) urogenital system cancer

Chemo												
-0.42 (-3.92, 3.26)	CTLA4											
-2.85 (-7.93, 0.61)	-2.51 (-8.36, 2.12)	PD1										
27.5 (-1.16, 90.7)	27.92 (-1.11, 91.26)	**30.57 (2.1, 94.02)**	PD1+Chemo									
0.22 (-3.12, 3.95)	0.63 (-3.18, 4.72)	3.18 (-0.85, 8.47)	-27.27 (-90.48, 1.63)	PD1+CTLA4								
25.51 (-2.56, 87.9)	25.89 (-2.43, 88.46)	**28.53 (1.11, 90.59)**	-2.11 (-71.44, 66.5)	25.22 (-2.93, 87.59)	PD1+PDL1							
-2.56 (-8.18, 2.47)	-2.18 (-8.38, 3.48)	0.36 (-3.83, 5.07)	**-30.24 (-93.53, -1.2)**	-2.82 (-8.29, 1.94)	**-28.14 (-90.45, -0.1)**	PD1+TTD						
-1.45 (-4.66, 1.42)	-1.05 (-5.93, 3.45)	1.43 (-3.24, 7.13)	**-29.02 (-92.23, -0.09)**	-1.68 (-6.65, 2.66)	-27.01 (-89.55, 1.3)	1.12 (-4.86, 7.33)	PDL1					
-1.16 (-4.37, 1.78)	-0.76 (-5.64, 3.81)	1.72 (-2.97, 7.5)	-28.74 (-91.88, 0.19)	-1.4 (-6.37, 3)	-26.7 (-89.15, 1.62)	1.41 (-4.6, 7.67)	0.28 (-3.49, 4.11)	PDL1+Chemo				
-2.18 (-7.65, 2.59)	-1.8 (-8.37, 4.13)	0.72 (-5.65, 7.59)	**-29.78 (-93.09, -0.4)**	-2.43 (-9.1, 3.37)	-27.82 (-90.43, 1.03)	0.38 (-6.99, 7.66)	-0.76 (-5.5, 3.81)	-1.03 (-6.9, 4.5)	PDL1+CTLA4			
**-15.36 (-49.38, -2.18)**	**-15.03 (-49.18, -1.34)**	-12.29 (-46.24, 1.12)	**-46.44 (-116.35, -8.58)**	**-15.66 (-49.73, -2.26)**	**-44.54 (-111.72, -7.28)**	-12.73 (-46.94, 0.81)	**-13.91 (-47.8, -0.7)**	**-14.2 (-48.27, -0.65)**	-13.25 (-47.25, 1.02)	PDL1+TTD		
**31.63 (2.87, 101.75)**	**32.02 (3.35, 101.85)**	**34.78 (5.45, 104.9)**	3.75 (-68.67, 81.23)	**31.32 (2.42, 101.47)**	6.04 (-63.54, 81.72)	**34.3 (4.73, 104.47)**	**33.1 (4.3, 103.49)**	**32.79 (3.8, 103.13)**	**33.9 (4.54, 104.16)**	**51.2 (12.22, 124.99)**	Placebo	
0.76 (-3.81, 5.11)	1.14 (-4.07, 6.2)	**3.69 (0.33, 7.85)**	-26.81 (-90.07, 1.97)	0.5 (-3.68, 4.4)	-24.75 (-86.97, 3.14)	**3.28 (0.24, 7.01)**	2.19 (-3.08, 7.64)	1.91 (-3.45, 7.38)	2.93 (-3.59, 9.95)	**16.08 (3.14, 50.15)**	**-30.91 (-100.94, -1.62)**	TTD

(C) skin cancer

Chemo								
-9.87<br>(-36.14, 0.93)	CTLA4							
**-16.31<br>(-53.26, -0.65)**	-5.84<br>(-44.26, 24.77)	CTLA4+Chemo						
-9.71<br>(-36.01, 0.71)	0.05<br>(-1.99, 2.12)	5.91<br>(-24.55, 44.39)	PD1					
**-11.32<br>(-37.8, -0.42)**	-1.45<br>(-4.14, 1.18)	4.38<br>(-26.25, 42.93)	-1.51<br>(-4.23, 1.09)	PD1+CTLA4				
-9.78<br>(-36.06, 1.25)	0.05<br>(-3.51, 3.65)	5.88<br>(-24.71, 44.44)	-0.01<br>(-2.97, 2.97)	1.52<br>(-2.41, 5.51)	PD1+TTD			
**-30.76<br>(-68.2, -5.49)**	**-17.56<br>(-51.77, -1.16)**	-12.56<br>(-56.12, 30.27)	**-17.62<br>(-51.84, -1.51)**	-16.11<br>(-50.43, 0.38)	**-17.65<br>(-51.93, -1.09)**	PDL1+TTD		
13.8<br>(-20.86, 66.89)	**25.37<br>(3.62, 75)**	34.1<br>(-7.78, 90.38)	**25.37<br>(3.6, 75.13)**	**26.88<br>(4.93, 76.75)**	**25.35<br>(3.28, 75.27)**	**46.26<br>(10.75, 102.88)**	Placebo	
-6.53<br>(-53.99, 48.28)	4.42<br>(-37.72, 60.06)	12.11<br>(-41.12, 72.44)	4.36<br>(-37.03, 60.03)	5.89<br>(-35.63, 61.63)	4.38<br>(-37.27, 60.02)	**22.5<br>(1.51, 73.96)**	-22.02<br>(-86.73, 37.87)	TTD

(D) head and neck cancer

Chemo									
9.47<br>(-3.4, 45.71)	CTLA4								
**-2.64<br>(-6.67, -0.15)**	-12.38<br>(-48.92, 0.42)	PD1							
**-3.7<br>(-8.45, -0.58)**	**-13.49<br>(-50.11, -0.32)**	-1.01<br>(-3.81, 1.36)	PD1+Chemo						
**-18.05<br>(-52.46, -3.11)**	**-30.9<br>(-76.26, -5.39)**	**-15.16<br>(-49.92, -0.55)**	-14.1<br>(-49.18, 0.83)	PD1+TTD					
-2.12<br>(-5.64, 0.51)	-11.72<br>(-48.02, 0.64)	0.51<br>(-2.27, 3.92)	1.57<br>(-1.94, 5.93)	**15.77<br>(0.71, 50.45)**	PDL1				
-2.13<br>(-5.79, 0.65)	-11.8<br>(-48.18, 1.67)	0.51<br>(-3.83, 5.48)	1.55<br>(-3.12, 7.11)	**15.96<br>(0.44, 50.4)**	-0.03<br>(-4.44, 4.49)	PDL1+Chemo			
-2.42<br>(-5.9, 0.15)	-12<br>(-48.31, 0.33)	0.21<br>(-2.5, 3.59)	1.25<br>(-2.17, 5.62)	**15.49<br>(0.42, 50.18)**	-0.31<br>(-1.89, 1.3)	-0.29<br>(-4.74, 4.14)	PDL1+CTLA4		
-2.15<br>(-5.9, 0.59)	-11.79<br>(-48.13, 0.83)	0.51<br>(-1.69, 3.1)	1.54<br>(-1.57, 5.34)	**15.75<br>(0.84, 50.65)**	-0.01<br>(-2.49, 2.26)	0.01<br>(-4.72, 4.46)	0.29<br>(-2.12, 2.46)	Placebo	
-2.79<br>(-7.71, 0.83)	-12.54<br>(-49.15, 0.71)	-0.12<br>(-3.09, 2.79)	0.9<br>(-1.62, 3.72)	**15.05<br>(0, 50.19)**	-0.65<br>(-5.16, 3.23)	-0.62<br>(-6.37, 4.32)	-0.34<br>(-4.84, 3.47)	-0.64<br>(-4.58, 2.92)	TTD+Chemo

(E) digestive system cancer

Chemo									
**-2.27 (-4.88, -0.35)**	PD1								
-2.21 (-5.5, 0.58)	0.03 (-3.05, 3.38)	PD1+Chemo							
**-21.19 (-63.94, -4.62)**	**-18.84 (-61.76, -2.23)**	**-18.85 (-61.75, -2.6)**	PD1+CTLA4						
**-23.07 (-72.13, -1.38)**	-20.75 (-69.84, 1.18)	-20.82 (-70, 1.25)	-0.68 (-54.33, 42.72)	PD1+TTD					
-1.72 (-6.78, 2.88)	0.54 (-4.71, 6)	0.49 (-5.2, 6.24)	**19.44 (1.81, 62.28)**	21.36 (-1.24, 70.52)	PDL1				
-6.95 (-76.91, 44.9)	-4.63 (-74.52, 47.21)	-4.78 (-74.61, 47.22)	15.51 (-55.95, 82.14)	18.06 (-58.45, 87.29)	-5.22 (-75.25, 46.76)	PDL1+CTLA4			
-1.67 (-6.8, 2.94)	0.59 (-4.71, 6.06)	0.55 (-5.22, 6.27)	**19.52 (1.86, 62.16)**	21.38 (-1.22, 70.44)	0.05 (-6.85, 6.87)	5.23 (-46.99, 75.31)	PDL1+CTLA4+Chemo		
-0.92 (-8.15, 6.13)	1.36 (-5.94, 9.04)	1.31 (-6.38, 9.18)	**20.39 (1.53, 63.34)**	22.24 (-1.31, 71.56)	0.83 (-4.44, 6.18)	6.07 (-45.98, 76.29)	0.78 (-7.82, 9.45)	PDL1+TTD	
20.45 (-0.26, 60.44)	**22.79 (2.29, 62.81)**	**22.79 (1.89, 62.79)**	**43.49 (12.19, 104.1)**	**47.65 (8.94, 103.77)**	**22.34 (0.76, 62.32)**	**28.17 (0.81, 91.93)**	**22.25 (0.69, 62.62)**	21.61 (-0.93, 61.59)	Placebo
**29.31 (1.67, 100.56)**	**31.65 (4.06, 102.82)**	**31.61 (3.75, 103.06)**	**55 (14.98, 131.05)**	**57.61 (11.82, 141.88)**	**31.11 (3.21, 102.48)**	40.72 (-25.29, 137.13)	**31.11 (2.76, 102.39)**	**30.31 (1.84, 101.7)**	8.69 (-41.83, 84.37)

Table S7. Model Selection of All Outcomes

Comparison of the fit of consistency and inconsistency model using deviance information criteria (DIC)				
	MODEL			
**outcomes**	**consistency,fixed**	**consistency,random**	**inconsistency**	**PSRF**
**All-grade Tr-PAEs**	703.7917	547.6197	556.0919	1.19
**Grade3-4 Tr-PAEs**	488.1327	456.8177	464.4705	1.17
**Fatal PAEs**	158.85443	159.70449	162.75541	1.18

Table S8. Inconsistency Test Between Direct and Indirect Treatment Comparisons in Mixed Treatment Comparisons for (A)all-grade treatment-related PAEs,(B)grade 3-4 treatment-related PAEs,(C)fatal PAEs

A. All-grade treatment-related PAEs

Side	Direct		Indirect		Difference		
	Coef.	Std. Err.	Coef.	Std. Err.	Coef.	Std. Err.	P>z
A C	-2.350123	1.601566	0.2094313	0.4189967	-2.559555	1.655313	0.122
A E	1.362521	0.311908	1.072779	0.4529323	0.2897427	0.5464719	0.596
A F	0.9205155	0.4522462	1.165485	0.4296995	-0.2449696	0.6206125	0.693
A G	1.680885	0.9357411	0.4213663	0.4835466	1.259519	1.053297	0.232
A L	-0.849981	1.052562	1.30529	0.4443372	-2.155271	1.145763	0.06
A M	1.723672	0.7786326	1.604044	0.3813169	0.1196286	0.867692	0.89
A N	0.9240764	0.8405671	1.689935	0.4364163	-0.7658583	0.9486361	0.419
B E	1.772901	0.2066284	0.8123633	0.3168104	0.9605381	0.3780175	0.111
B F	1.149959	0.3742743	1.439567	0.4213403	-0.2896078	0.5656175	0.609
B G	-0.2965388	0.6652396	1.49869	0.4746896	-1.795229	0.7960809	0.024
B H	0.7081886	0.2375496	1.312547	0.4547172	-0.6043581	0.5206062	0.246
B I *	0.8227199	0.3038083	1.28486	1.536862	-0.4621401	1.583351	0.77
B J *	0.5529454	0.688027	0.5696789	385.7154	-0.0167335	385.7161	1
B K	1.893203	1.135674	1.520656	0.4899315	0.372547	1.247747	0.765
B M	1.16858	0.4792795	2.216794	0.3441832	-1.048213	0.6056366	0.083
B N	2.167829	0.511103	1.340159	0.5112433	0.8276706	0.7579703	0.275
B P *	3.735811	1.556121	-1.965384	353.397	5.701195	353.406	0.987
B Q	0.9400841	0.6251616	1.564062	0.7491607	-0.6239777	1.01645	0.539
C E	0.697633	0.5459294	1.583294	0.4526364	-0.8856612	0.7019443	0.207
C F	2.050168	0.8999941	0.7830977	0.4186971	1.26707	0.9872451	0.199
C K	1.155372	0.5233319	1.59284	0.6834486	-0.437468	0.8455463	0.605
C L	0.9245312	0.4507418	0.9581692	0.6973516	-0.0336381	0.8262087	0.968
C M	2.750395	1.197147	1.42664	0.4396093	1.323755	1.27531	0.299
D E *	0.6488428	1.058973	2.710994	1.704233	-2.062151	1.877811	0.272
D H *	0.9553927	1.034956	-1.106602	1.747933	2.061995	1.877811	0.272
D O *	1.333728	0.7235379	-0.5728854	379.2563	1.906614	379.2576	0.996
E G	-0.5626757	0.6576773	-0.5932163	0.486686	0.0305406	0.8181667	0.97
E H *	-0.2007636	0.4431941	-0.8322682	0.2779084	0.6315046	0.5234081	0.228
E K	0.2869751	0.6177928	-0.1005938	0.5911696	0.3875689	0.8596354	0.652
E L	1.927633	1.630248	-0.4267207	0.3959858	2.354354	1.677651	0.161
E M	0.7488857	0.3550934	-0.0974794	0.3840663	0.8463651	0.526735	0.108
E R *	1.98E-09	1.34507	-2.539136	282.7516	2.539136	282.7565	0.993
F G	-0.5896345	1.166057	-0.329005	0.476252	-0.2606294	1.231933	0.832
F I	0.0270565	1.540491	-0.465274	0.4096866	0.4923304	1.594038	0.757
F L	-0.3352391	0.3998887	0.6438248	0.6678686	-0.9790639	0.7796534	0.209
F N	0.5789148	0.3855913	0.1759491	0.6778325	0.4029658	0.7926909	0.611

G M	0.5001679	0.5846488	1.375887	0.5817341	-0.8757189	0.8179123	0.284
G N	0.8583061	1.038019	0.8336908	0.5473442	0.0246152	1.15357	0.983
H M	0.8532242	0.4907367	1.099849	0.3703592	-0.2466247	0.6152092	0.689
I Q	0.2008759	0.7536675	0.4763967	0.6627766	-0.2755208	1.004768	0.784
N Q	0.0067339	0.7679838	-0.9634587	0.6560435	0.9701927	1.010046	0.337

A=Placebo;B=Chemo;C=TTD;D=TTD+Chemo;E=PD1;F=PDL1;G=CTLA4;H=PD1+Chemo;I=PDL1+Chemo;J=CTLA4+Chemo;K=PD1+TTD;L=PDL1+TTD;M=PD1+CTLA4;N=PDL1+CTLA4;O=PDL1+TTD+Chemo;P=PD1+CTLA4+Chemo;Q=PDL1+CTLA4+Chemo;R=PD1+PDL1

B. Grade 3-4 treatment-related PAEs

Side	Direct		Indirect		Difference		
	Coef.	Std. Err.	Coef.	Std. Err.	Coef.	Std. Err.	P>z
A C	-1.324845	1.992432	-0.3241157	0.4572346	-1.000729	2.04422	0.624
A E	0.8862845	0.3525741	0.7395543	0.4592968	0.1467302	0.5572676	0.792
A F	0.4162884	0.4905191	1.102279	0.4543319	-0.6859907	0.6686054	0.305
A G	1.380599	0.9279708	0.3995141	0.5057231	0.9810844	1.056828	0.353
A L	-2.058336	1.993266	1.108224	0.5653927	-3.16656	2.071786	0.126
A M	1.994822	0.9914708	1.403974	0.3680524	0.5908482	1.041658	0.571
A N	0.9838031	0.6691633	0.9190598	0.4635563	0.0647434	0.8127324	0.937
B E	1.392618	0.2160784	0.5888849	0.3356992	0.8037326	0.4014469	0.045
B F	1.224074	0.3521635	0.9071251	0.4619257	0.316949	0.5815754	0.586
B G	-0.0887231	0.7188002	1.39852	0.4792613	-1.487243	0.8560343	0.082
B H	0.454459	0.2816811	1.338572	0.4341107	-0.8841128	0.5332406	0.097
B I *	0.634496	0.2939356	2.244054	1.55231	-1.609558	1.591487	0.312
B J *	-0.1120233	0.7132097	0.8969898	426.975	-1.009013	426.9754	0.998
B K	1.860845	1.045408	1.421213	0.5582456	0.4396319	1.202306	0.715
B M	1.384345	0.5114119	1.939901	0.3020511	-0.5555559	0.6068405	0.36
B N	1.496662	0.5769313	1.100807	0.4680908	0.3958541	0.7824928	0.613
B P *	3.019133	1.445543	-1.107214	503.8854	4.126348	503.8913	0.993
B Q	0.7353738	0.6871241	0.8438068	0.7308009	-0.108433	1.026038	0.916
C E	0.9702267	0.5180412	1.441142	0.5139123	-0.4709152	0.7297065	0.519
C F	0.9773775	1.038652	1.197949	0.4715778	-0.2205714	1.14507	0.847
C K	1.877244	0.7004205	1.276645	0.6946491	0.6005992	0.9864722	0.543
C L	0.8775048	0.5686131	2.193536	0.9195288	-1.316032	1.090851	0.228
C M	2.750397	1.02898	1.67305	0.445033	1.077347	1.121095	0.337
D E *	-0.0443002	0.9965853	2.560137	1.489477	-2.604438	1.792212	0.146
D H *	0.7322343	0.8619118	-1.872165	1.723187	2.604399	1.792205	0.146
D O *	0.8268646	1.094457	0.0767284	659.2438	0.7501362	659.2458	0.999
E G	0.0338664	0.6651881	-0.3417612	0.4911897	0.3756276	0.8268886	0.65
E H *	0.0447778	0.3793749	-0.7232436	0.2996871	0.7680214	0.4826202	0.112
E K	0.0277335	0.6492544	0.7558426	0.6967872	-0.7281091	0.9672495	0.452
E L	1.927636	1.508832	-0.1929496	0.5289984	2.120585	1.598879	0.185

E M	0.7494222	0.2788795	0.4189212	0.3868684	0.330501	0.4822832	0.493
E R *	-1.76877	1.611818	-1.844221	354.7629	0.0754514	354.7627	1
F G	-0.5690482	1.211877	-0.102886	0.4804909	-0.4661622	1.285359	0.717
F I	0.0270554	1.410375	-0.4422017	0.4054303	0.4692571	1.467492	0.749
F L	-0.0555487	0.6633936	0.2732227	0.7579703	-0.3287713	1.007189	0.744
F N	0.0926433	0.3518215	0.3826795	0.7004705	-0.2900362	0.7998512	0.717
G M	0.6480145	0.5789854	1.037267	0.5737054	-0.3892527	0.8058276	0.629
G N	1.038132	0.9883208	0.113695	0.5402264	0.9244367	1.103329	0.402
H M	0.9924085	0.4369164	1.109575	0.3503134	-0.1171668	0.5607046	0.834
I Q	-0.2580076	0.7583323	0.3768441	0.6997614	-0.6348517	1.023071	0.535
N Q	-0.2809474	0.7548941	-0.6442561	0.7028094	0.3633087	1.03141	0.725

A=Placebo;B=Chemo;C=TTD;D=TTD+Chemo;E=PD1;F=PDL1;G=CTLA4;H=PD1+Chemo;I=PDL1+Chemo;J=CTLA4+Chemo;K=PD1+TTD;L=PDL1+TTD;M=PD1+CTLA4;N=PDL1+CTLA4;O=PDL1+TTD+Chemo;P=PD1+CTLA4+Chemo;Q=PDL1+CTLA4+Chemo;R=PD1+PDL1

C. Fatal PAEs

Side	Direct		Indirect		Difference		
	Coef.	Std. Err.	Coef.	Std. Err.	Coef.	Std. Err.	P>z
A C	-1.313766	1.992429	-0.5899548	0.67775	-0.723811	2.104471	0.731
A E	-0.0722055	0.5032663	-0.3655092	0.6747226	0.2933037	0.8467655	0.729
A F	0.4217479	0.6990044	-0.4501525	0.6406436	0.8719004	0.9411745	0.354
A G	-0.0230648	1.067937	0.2611955	0.7327438	-0.2842603	1.295146	0.826
A L	-2.052327	1.993262	-0.6039948	0.708136	-1.448332	2.115236	0.494
A M	-1.250626	1.580843	-0.1613754	0.6087592	-1.08925	1.690128	0.519
A N	-0.6910186	1.411374	0.7971868	0.6588737	-1.488205	1.557587	0.339
B E	0.387194	0.2950448	0.3481841	0.5174496	0.0390099	0.5956504	0.948
B F	0.1846515	0.5338067	0.9579576	0.6390116	-0.7733061	0.8326357	0.353
B G	0.6183867	1.149679	0.7609046	0.6623552	-0.1425179	1.327028	0.914
B H	0.1752549	0.3905891	0.1747934	0.7791225	0.0004616	0.8631088	1
B I *	0.1105618	0.4966773	0.8490498	2.804673	-0.738488	2.84831	0.795
B J *	0.004465	0.998843	1.108429	659.5241	-1.103964	659.5249	0.999
B K	-0.0224684	1.408677	0.0243288	0.8617747	-0.0467972	1.651371	0.977
B M	0.23897	0.7829717	0.2641855	0.5935227	-0.0252155	0.9825041	0.98
B N	1.463503	0.6584111	0.4056248	0.90343	1.057878	1.155873	0.36
B P *	1.073223	1.631265	0.8192242	1196.263	0.2539988	1196.266	1
B Q	-0.3513357	1.41144	0.9454112	1.792577	-1.296747	2.281557	0.57
C E	-0.0260757	0.9988999	0.7281353	0.6838503	-0.754211	1.210559	0.533
C F	1.180922	1.041336	0.3673615	0.6878714	0.8135602	1.239536	0.512
C K	-0.0116269	0.893636	0.3368637	1.137205	-0.3484907	1.446313	0.81
C L	0.0459178	0.6406637	-0.5695761	1.142798	0.6154939	1.310134	0.639
C M	-0.0221411	1.999077	0.4167311	0.7322418	-0.4388723	2.128964	0.837
D E *	1.054457	1.63091	1.703893	2.45763	-0.6494356	2.456315	0.791
D H *	1.137553	1.630822	0.4880843	2.457808	0.6494685	2.456317	0.791

D O *	0.0012677	1.41349	2.747114	1418.217	-2.745846	1418.218	0.998
E G	0.0412268	0.9991945	0.4894226	0.6792818	-0.4481958	1.208228	0.711
E H *	-0.3686472	0.7430144	-0.1365525	0.4708325	-0.2320947	0.8744393	0.791
E K	-0.1467589	1.148542	-0.51047	0.9322215	0.3637111	1.478927	0.806
E L	-0.0182654	1.997715	-0.6481177	0.6446296	0.6298524	2.099146	0.764
E M	0.0510645	0.638432	-0.3132137	0.6687155	0.3642782	0.924531	0.694
E R *	-0.6701577	1.982748	0.4651178	505.7908	-1.135275	505.7946	0.998
F G	0.3301429	1.409407	0.1956032	0.7108016	0.1345398	1.578576	0.932
F I	0.0269472	1.997302	-0.4126535	0.6591823	0.4396007	2.103269	0.834
F L	-1.10889	0.6629583	0.2330469	1.018707	-1.341937	1.206706	0.266
F N	0.8390319	0.6490708	-0.0149319	1.022723	0.8539638	1.246994	0.493
G M	-0.5873831	0.9955775	-0.3877033	0.836998	-0.1996799	1.30074	0.878
G N	-0.8621521	1.41029	0.7688028	0.8146771	-1.630955	1.628767	0.317
H M	-0.00563	1.150823	0.1046546	0.620435	-0.1102845	1.307413	0.933
I Q	0.0120113	1.998498	0.0118692	1.443879	0.0001422	2.46552	1
N Q	0.006689	1.996653	-1.417096	1.414902	1.423785	2.447155	0.561

A=Placebo;B=Chemo;C=TTD;D=TTD+Chemo;E=PD1;F=PDL1;G=CTLA4;H=PD1+Chemo;I=PDL1+Chemo;J=CTLA4+Chemo;K=PD1+TTD;L=PDL1+TTD;M=PD1+CTLA4;N=PDL1+CTLA4;O=PDL1+TTD+Chemo;P=PD1+CTLA4+Chemo;Q=PDL1+CTLA4+Chemo;R=PD1+PDL1

Figure S1.Flowchart of Study Selection

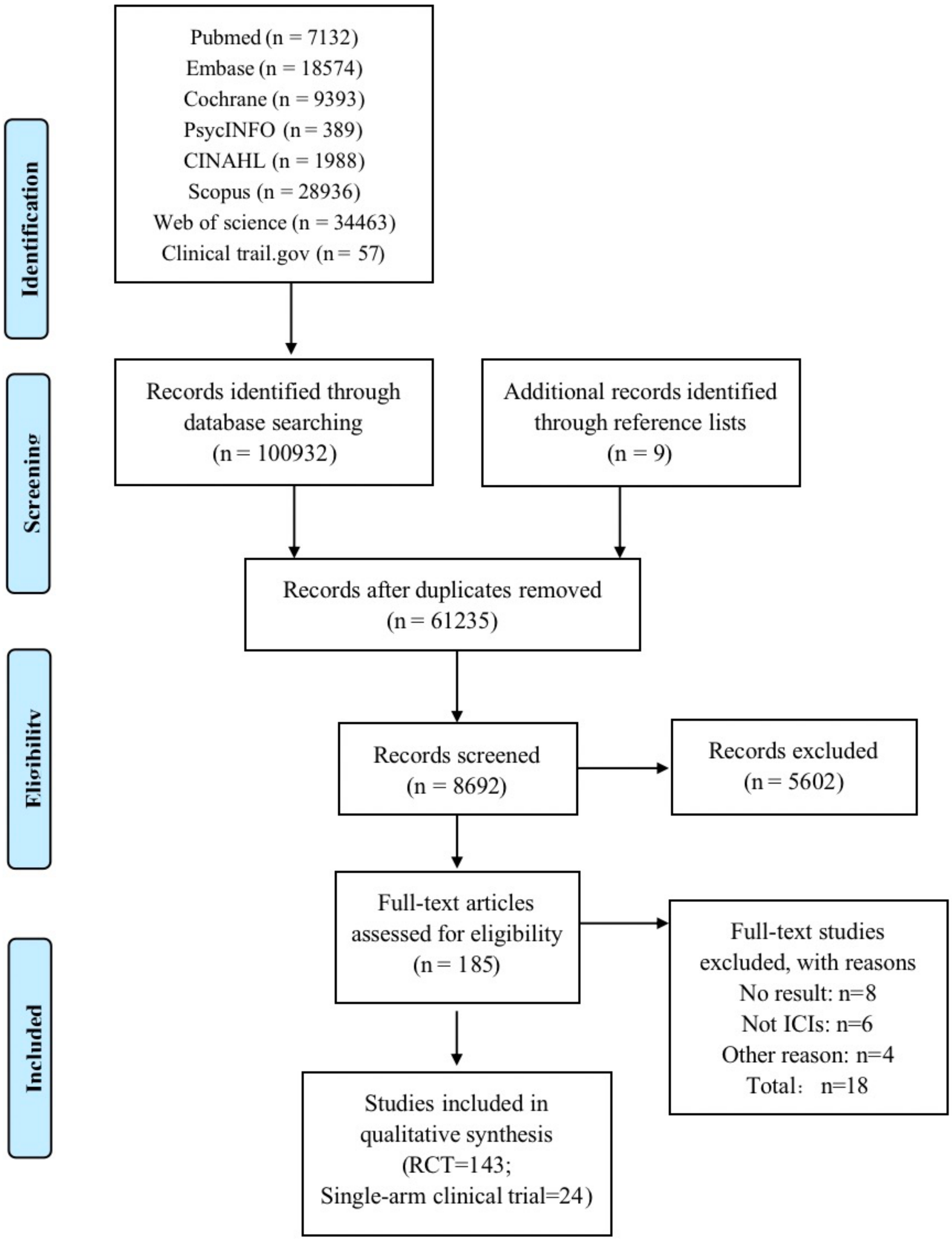

Figure S2. Risk of Bias Assessment for Studies

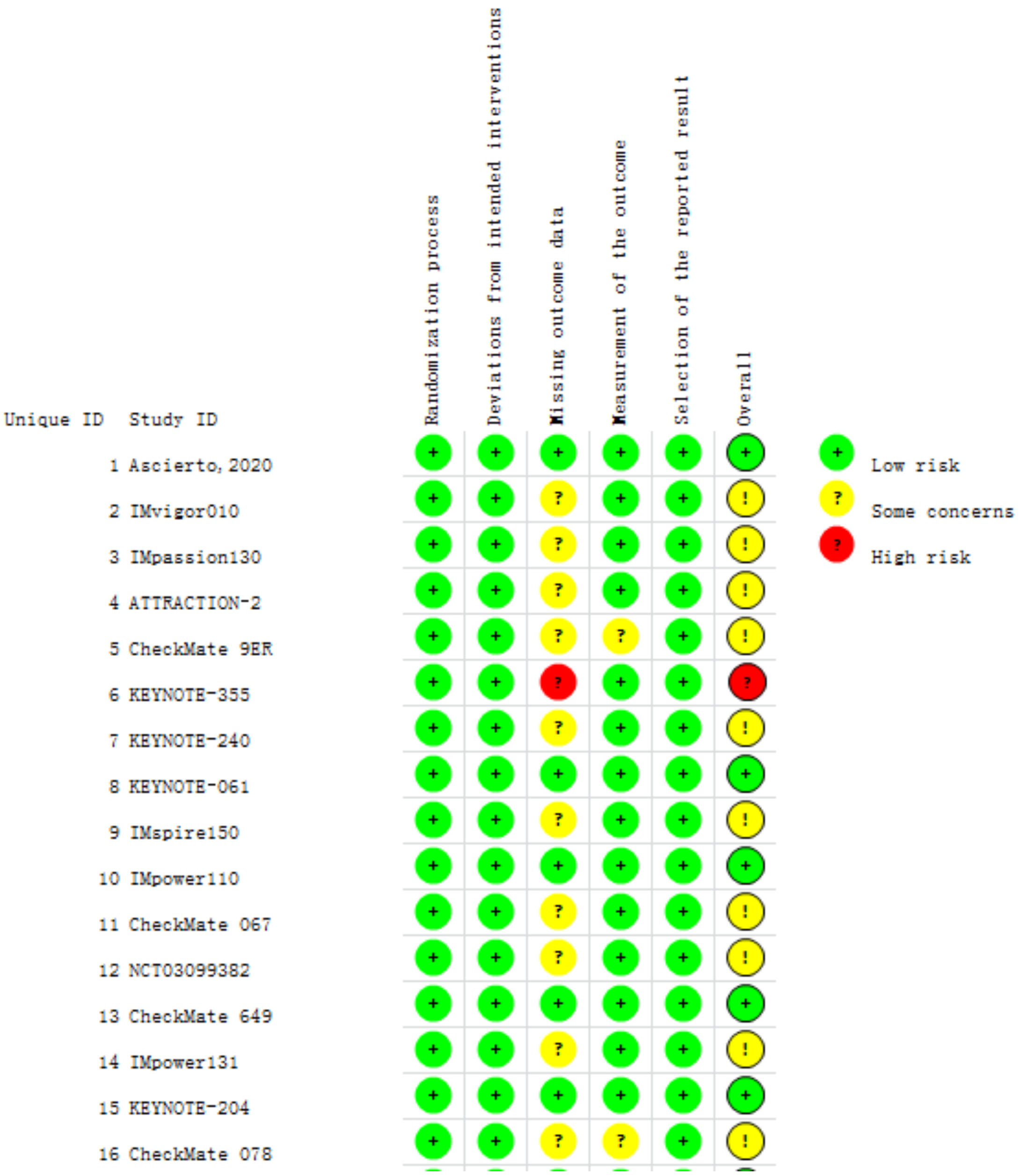

17 IMpassion131	+	+	+	+	+	+
18 IMpassion031	+	?	+	?	+	!
19 IMagyn050	+	+	+	+	+	+
20 CheckMate 025	+	+	?	?	+	!
21 KEYNOTE-045	+	+	?	+	+	!
22 PACIFIC	+	+	?	+	+	+
23 DANUBE	+	?	+	+	?	!
24 KEYNOTE-426	+	+	?	?	+	!
25 KEYNOTE-361	+	?	+	+	+	!
26 CheckMate 143	+	+	?	?	+	!
27 CheckMate 9LA	?	+	+	+	+	!
28 MYSTIC	+	?	?	?	+	?
29 KEYNOTE-604	+	+	+	+	+	+
30 Empwer-lung 1	+	?	+	?	+	!
31 KEYNOTE-062	+	+	+	+	+	+
32 NCT03117049	+	+	+	+	+	+
33 KEYNOTE-590	+	+	+	+	+	+
34 KEYNOTE-119	+	?	+	?	+	?
35 CAPTAIN-1st	+	+	?	+	+	!
36 ORIENT-12	+	+	+	+	+	+
37 CameL	+	?	+	?	+	!
38 CheckMate 141	+	+	?	?	+	!
39 CheckMate 017	+	+	?	?	+	!
40 CheckMate 057	+	+	?	?	+	!
41 KEYNOTE-024	+	+	?	?	+	+
42 KEYNOTE-006	+	?	?	?	+	?
43 CheckMate 026	+	+	?	?	+	!

44 OAK	+	+	?	?	+	!
45 KEYNOTE-407	+	+	+	+	+	!
46 KEYNOTE-042	+	"	?	?	+	"
47 CASPIAN	+	?	?	?	+	!
48 CheckMate 227	+	+	?	?	+	!
49 KEYNOTE-189	+	+	?	+	+	!
50 EORTC 18071	+	+	?	+	+	!
51 IMblaze370	+	+	?	?	+	!
52 KEYNOTE-040	+	+	+	?	+	+
53 KEYNOTE-054	+	+	?	+	+	!
54 KEYNOTE-252	+	+	+	+	+	+
55 CA184-156	+	+	?	+	+	!
56 IMpower150	+	+	?	?	+	!
57 KEYNOTE-181	+	+	?	?	+	!
58 IMbrave-150	+	+	?	?	+	!
59 CAURAL	+	+	?	?	+	!
60 IMpower133	+	+	+	+	+	+
61 CheckMate 451	+	+	?	+	+	!
62 KEYNOTE-042 China Study	+	"	?	?	+	"
63 IMspire170	+	?	+	?	+	!
64 IMvigor211	+	"	?	?	+	"
65 JAVELIN Lung 200	+	+	?	?	+	!
66 KEYNOTE-048	+	?	?	?	+	!

67 CheckMate 274	+	+	?	+	+	!
68 CONFIRM	+	+	+	+	+	+
69 KEYNOTE-598	+	+	+	+	+	+
70 KEYNOTE-185	+	?	?	?	+	!
71 NINJA	+	?	?	?	+	!
72 NCT03594747	+	?	?	?	+	!
73 CheckMate 066	+	+	+	+	+	!
74 CA184-104	+	+	?	+	+	!
75 KEYNOTE-522	+	+	+	+	+	+
76 ARCTIC	+	?	?	?	+	!
77 NCT03607539	+	+	+	+	+	+
78 CheckMate 037	+	+	?	+	+	!
79 EAGLE	+	+	?	?	+	!
80 JAVELIN OVARIAN 200	+	"	+	?	+	"
81 IMmotion151	+	+	?	?	+	!
82 CA184-095	+	+	?	+	+	!
83 CA184-024	+	+	?	+	+	!
84 CheckMate 214	+	+	+	?	"	"
85 IMpower130	+	+	?	?	+	+
86 CheckMate 743	+	"	?	?	+	"
87 JAVELIN HEAD AND NECK 100	+	?	?	+	+	!
88 JAVELIN Gastric 100	+	"	+	?	+	"
89 ATTRACTION-3	+	+	?	+	+	!

90 KEYNOTE-061	+	+	?	?	+	!
91 KEYNOTE-564	+	?	+	+	+	!
92 KEYNOTE-183	+	?	+	?	+	!
93 NCT02785952	+	?	?	?	+	!
94 KEYNOTE-581	+	+	+	?	+	!
95 RATIONALE 304	+	+	+	?	+	!
96 JAVELIN OVARIAN 100	+	?	+	?	+	!
97 CheckMate 331	+	+	?	?	+	!
98 KEYNOTE-672	+	+	+	+	?	!
99 KEYNOTE-669	+	+	+	?	?	!
100 NCT02279732	+	+	+	+	+	+
101 KEYNOTE-122	+	?	+	?	+	!
102 KEYNOTE-033	+	?	+	?	+	!
103 CheckMate 459	+	?	+	?	?	!
104 IMbassador250	+	?	+	?	+	!
105 Peters, 2021	+	+	+	+	+	+
106 Scherpereel, 2019	+	+	+	+	+	+
107 Voss, 2022	+	?	+	+	+	!
108 Loibl, 2019	+	+	+	+	+	+
109 McDermott, 2018	+	?	+	+	+	!
110 Zhou, 2023	+	?	+	+	+	!
111 Johnson, 2023	+	?	+	+	+	+
112 Forde, 2022	+	+	+	+	+	+

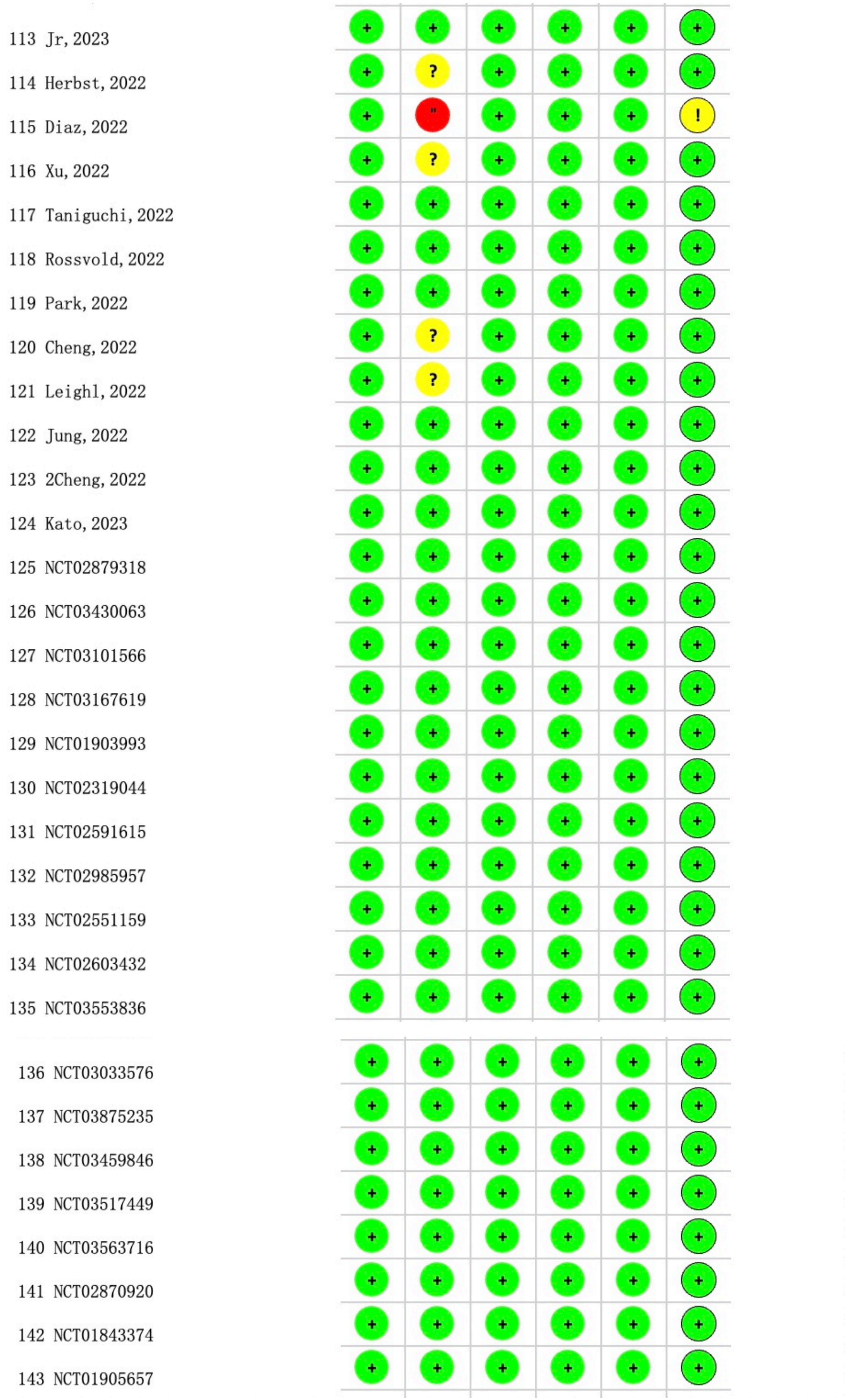

Figure S3. Comparison-adjusted funnel plots for (A)all-grade treatment-related PAEs,(B)grade 3-4 treatment-related PAEs,(C)fatal PAEs,(D)all-grade treatment-related PAEs in tumors of different systems;(E) grade 3-4 treatment-related PAEs in tumors of different systems.

A.

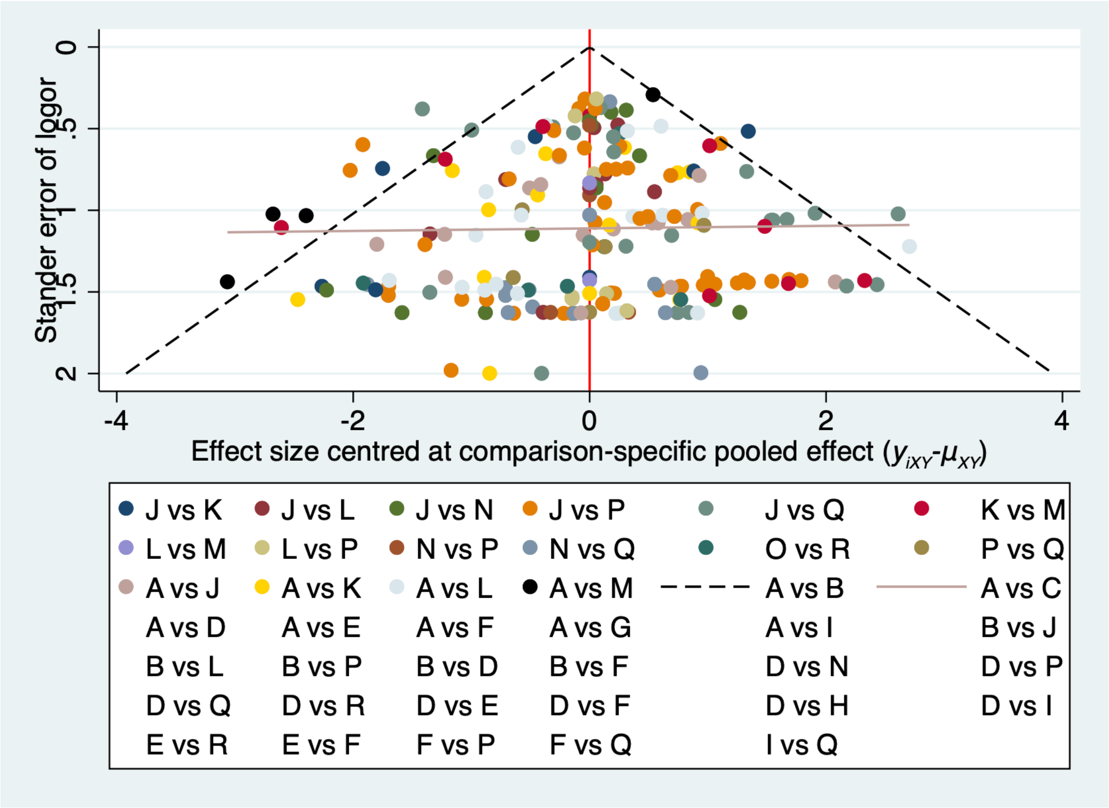

Egger test: P=0.113

A:Placebo; B:Chemo; C:TTD; D:TTD+Chemo; E:CTLA4; F:PDL1; G:PD1; H:CTLA4+Chemo; I:PDL1+Chemo; J:PD1+Chemo; K:PDL1+TTD; L:PD1+TTD; M:PDL1+CTLA4; N:PD1+CTLA4; O:PD1+PDL1; P:PDL1+TTD+Chemo; Q:PDL1+CTLA4+Chemo; R:PD1+CTLA4+Chemo

B.

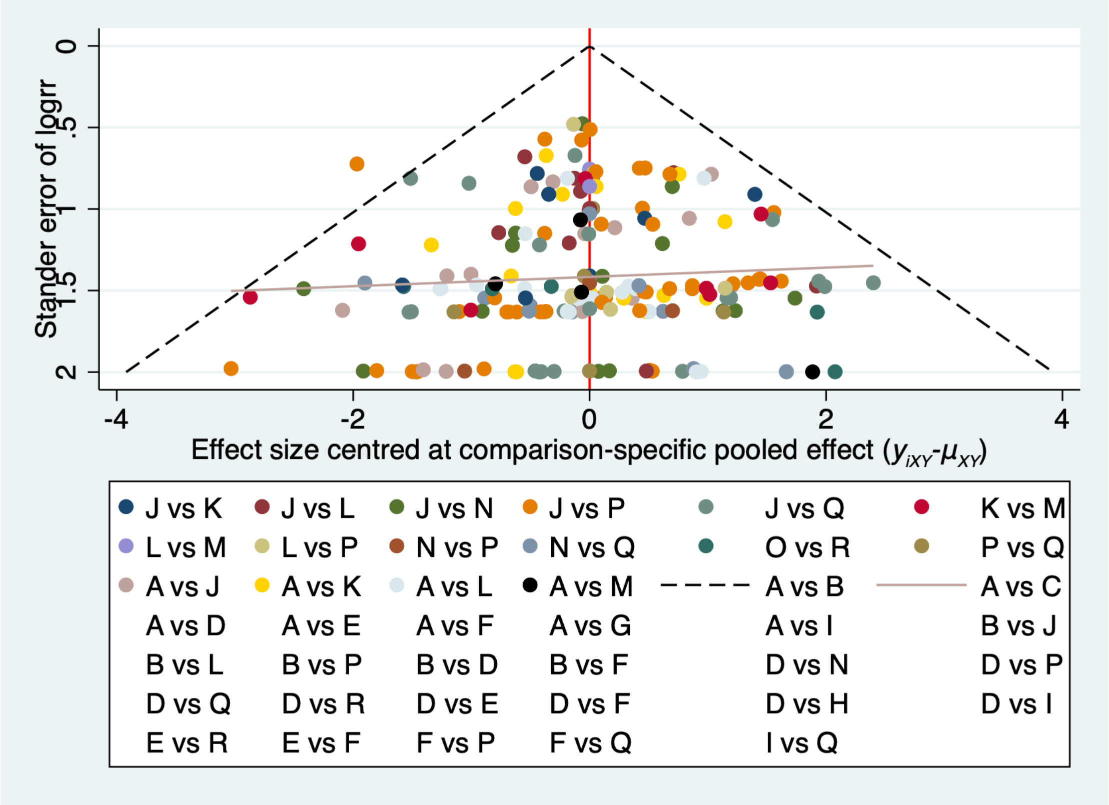

Egger test: P=0.544

A:Placebo; B:Chemo; C:TTD; D:TTD+Chemo; E:CTLA4; F:PDL1; G:PD1; H:CTLA4+Chemo; I:PDL1+Chemo; J:PD1+Chemo; K:PDL1+TTD; L:PD1+TTD; M:PDL1+CTLA4; N:PD1+CTLA4; O:PD1+PDL1; P:PDL1+TTD+Chemo; Q:PDL1+CTLA4+Chemo; R:PD1+CTLA4+Chemo

C.

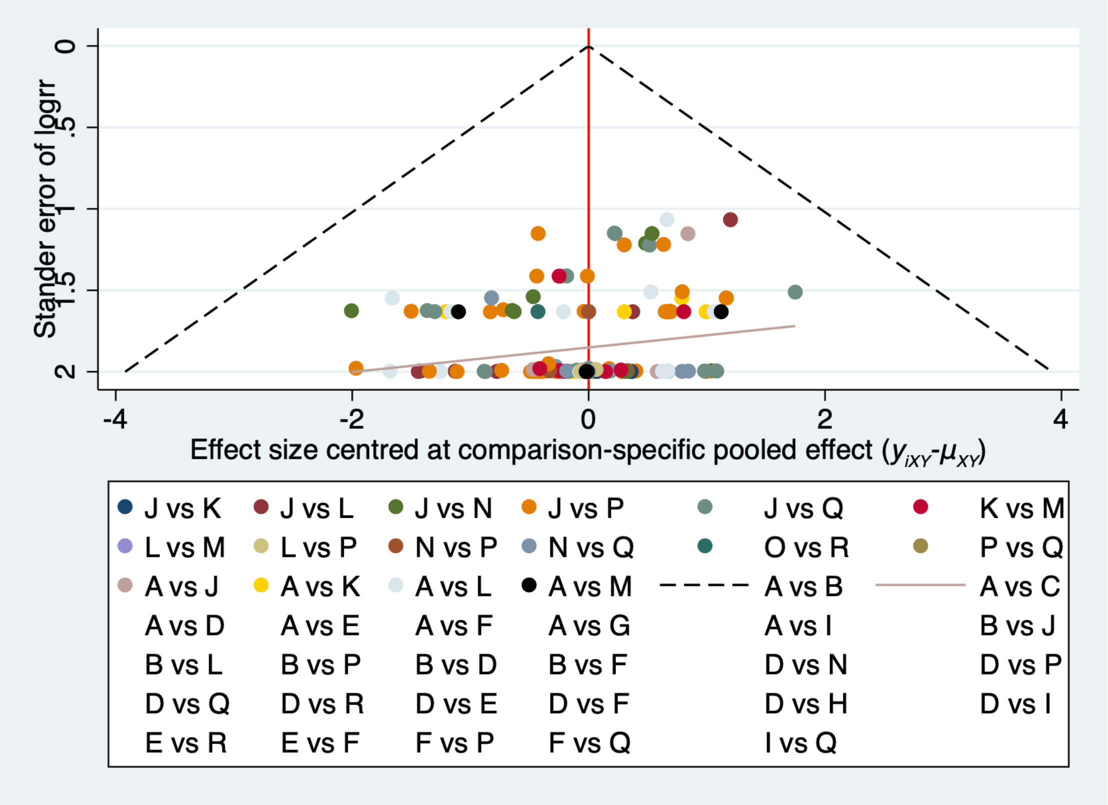

Egger test: P=0.000

A:Placebo; B:Chemo; C:TTD; D:TTD+Chemo; E:CTLA4; F:PDL1; G:PD1; H:CTLA4+Chemo; I:PDL1+Chemo; J:PD1+Chemo; K:PDL1+TTD; L:PD1+TTD; M:PDL1+CTLA4; N:PD1+CTLA4; O:PD1+PDL1; P:PDL1+TTD+Chemo; Q:PDL1+CTLA4+Chemo; R:PD1+CTLA4+Chemo

D.

(a) digestive system cancer

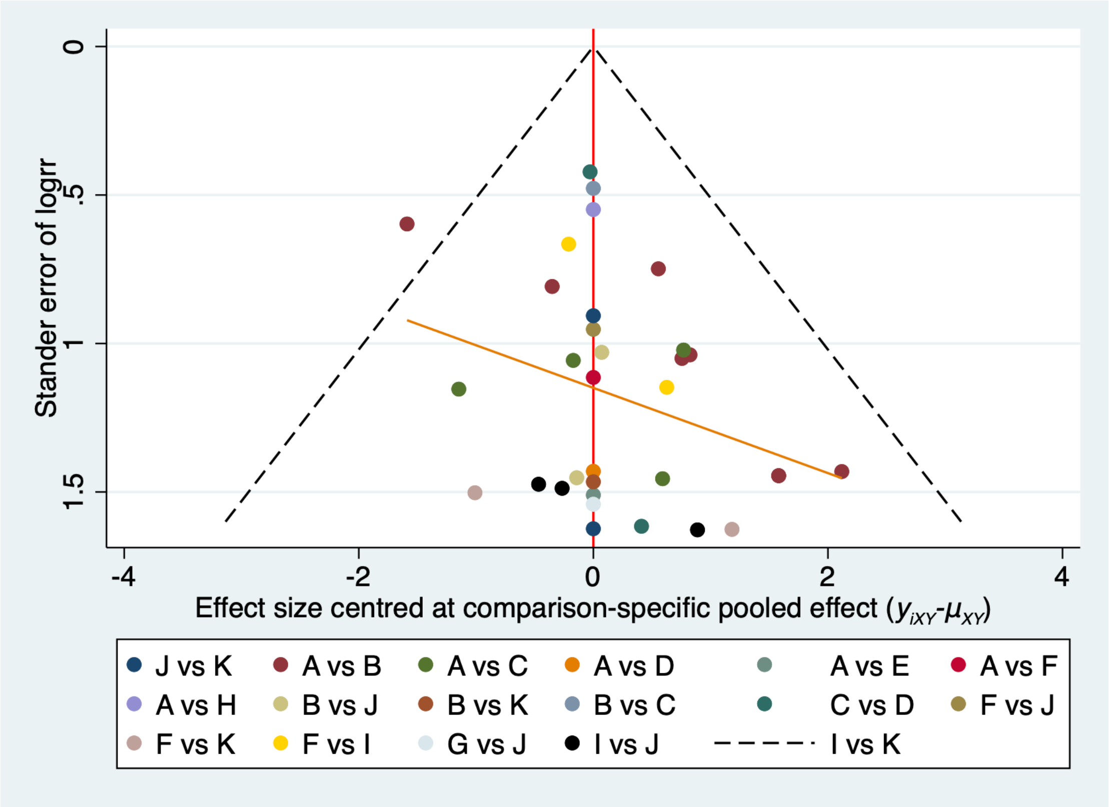

Egger test: P=0.081

A:Placebo; B:Chemo; C:TTD; D:PDL1; E:PD1; F:PD1+Chemo; G:PDL1+TTD; H:PD1+TTD; I:PDL1+CTLA4; J:PD1+CTLA4; K:PDL1+CTLA4+Chemo

(b) head and neck cancer

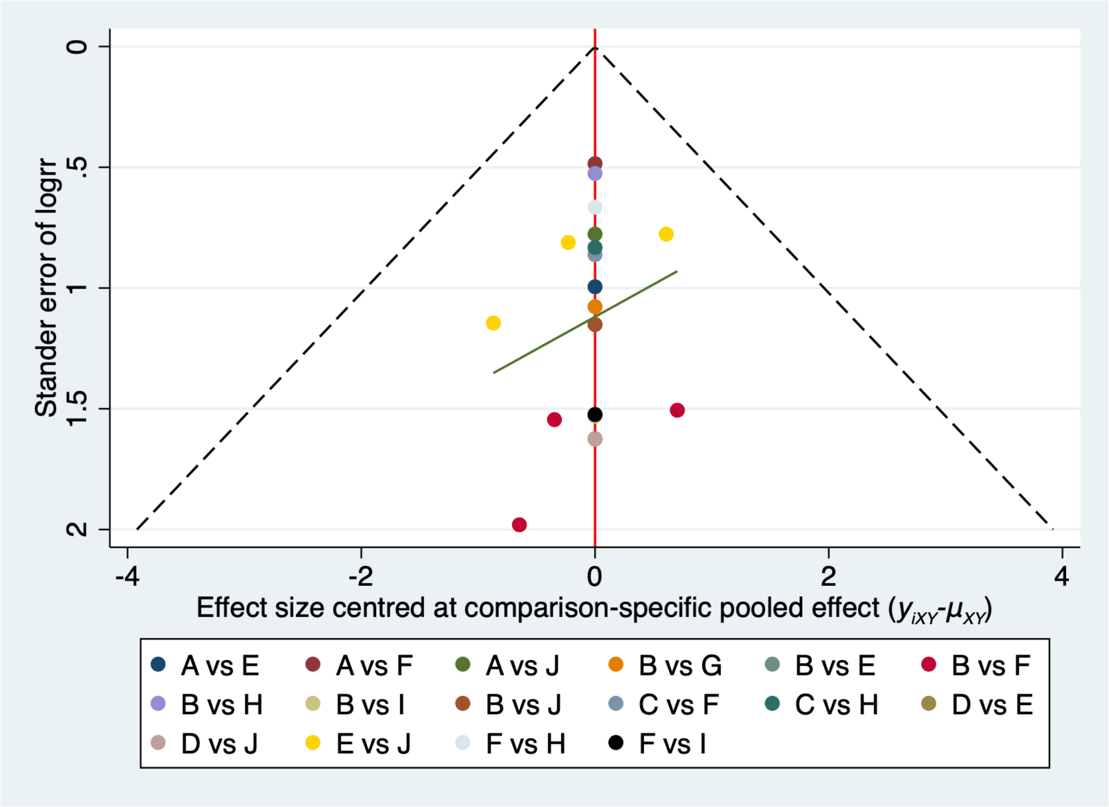

Egger test: P=0.165

A:Placebo; B:Chemo; C:TTD+Chemo; D:CTLA4; E:PDL1; F:PD1; G:PDL1+Chemo; H:PD1+Chemo; I:PD1+TTD; J:PDL1+CTLA4

(c) respiratory system cancer

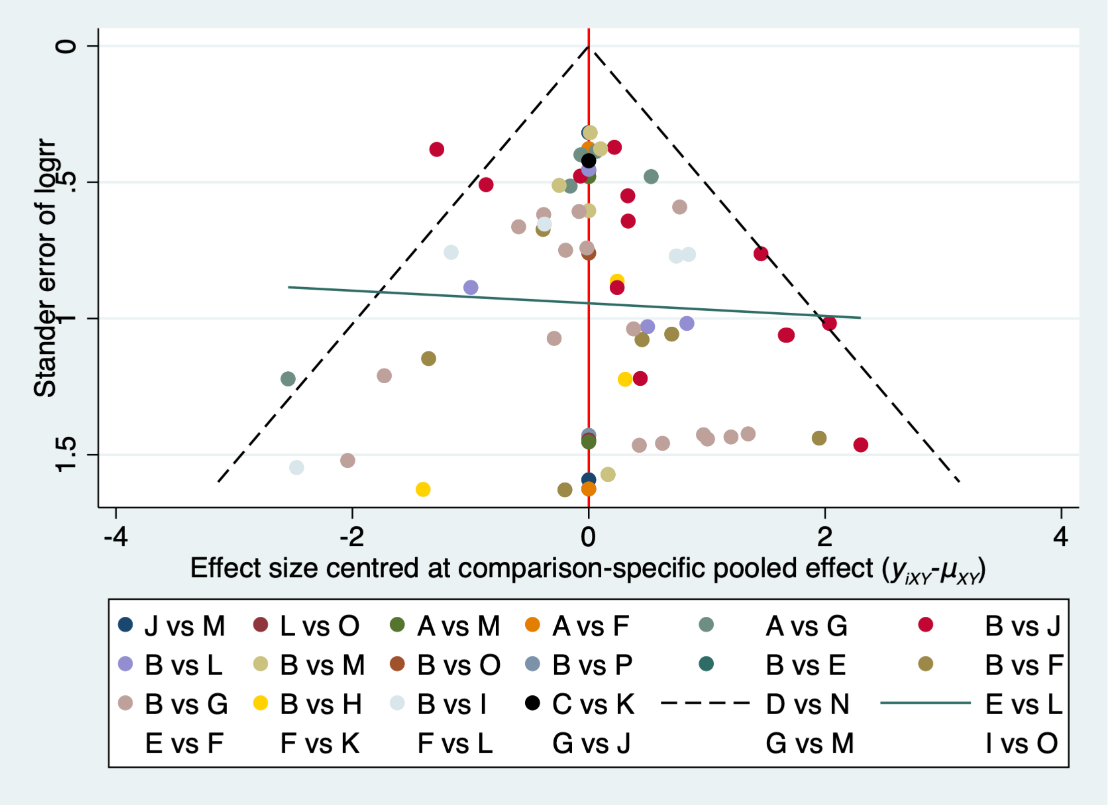

Egger test: P=0.000

A:Placebo; B:Chemo; C:TTD; D:TTD+Chemo; E:CTLA4; F:PDL1; G:PD1; H:CTLA4+Chemo; I:PDL1+Chemo; J:PD1+Chemo; K:PDL1+TTD; L:PDL1+CTLA4; M:PD1+CTLA4; N:PDL1+TTD+Chemo; O:PDL1+CTLA4+Chemo; P:PD1+CTLA4+Chemo

(d) skin cancer

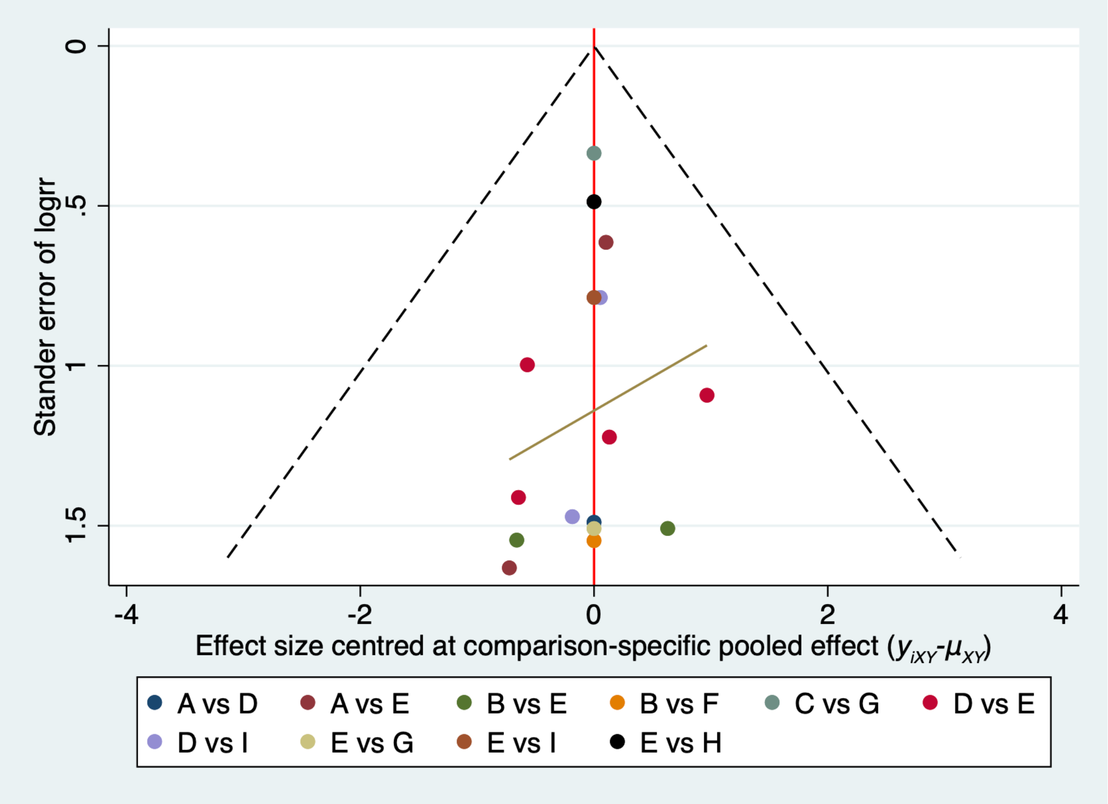

Egger test: P=0.219

A:Placebo; B:Chemo; C:TTD; D:CTLA4; E:PD1; F:CTLA4+Chemo; G:PDL1+TTD; H:PD1+TTD; I:PD1+CTLA4

(e) urogenital system cancer

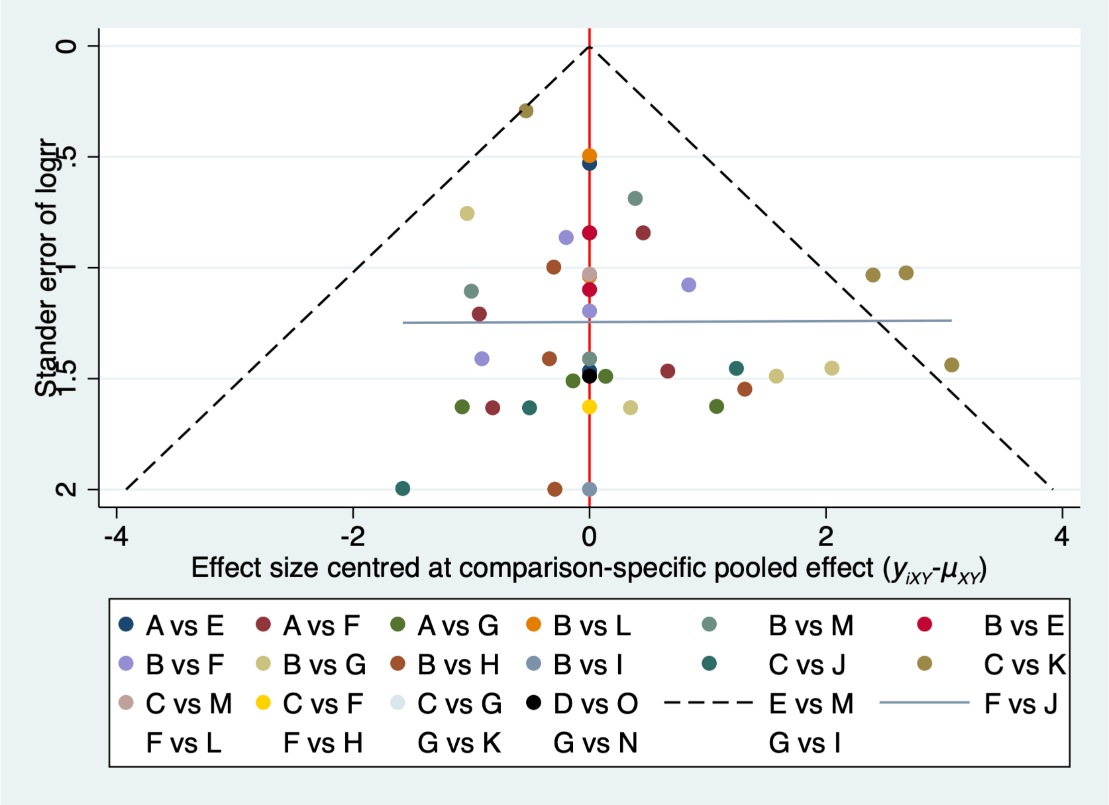

Egger test: P=0.050

A:Placebo; B:Chemo; C:TTD; D:TTD+Chemo; E:CTLA4; F:PDL1; G:PD1; H:CTLA4+Chemo; I:PDL1+Chemo; J:PD1+Chemo; K:PDL1+TTD; L:PD1+TTD; M:PDL1+CTLA4; N:PD1+CTLA4; O:PD1+PDL1; P:PDL1+TTD+Chemo; Q:PDL1+CTLA4+Chemo; R:PD1+CTLA4+Chemo

E.

(a) digestive system cancer

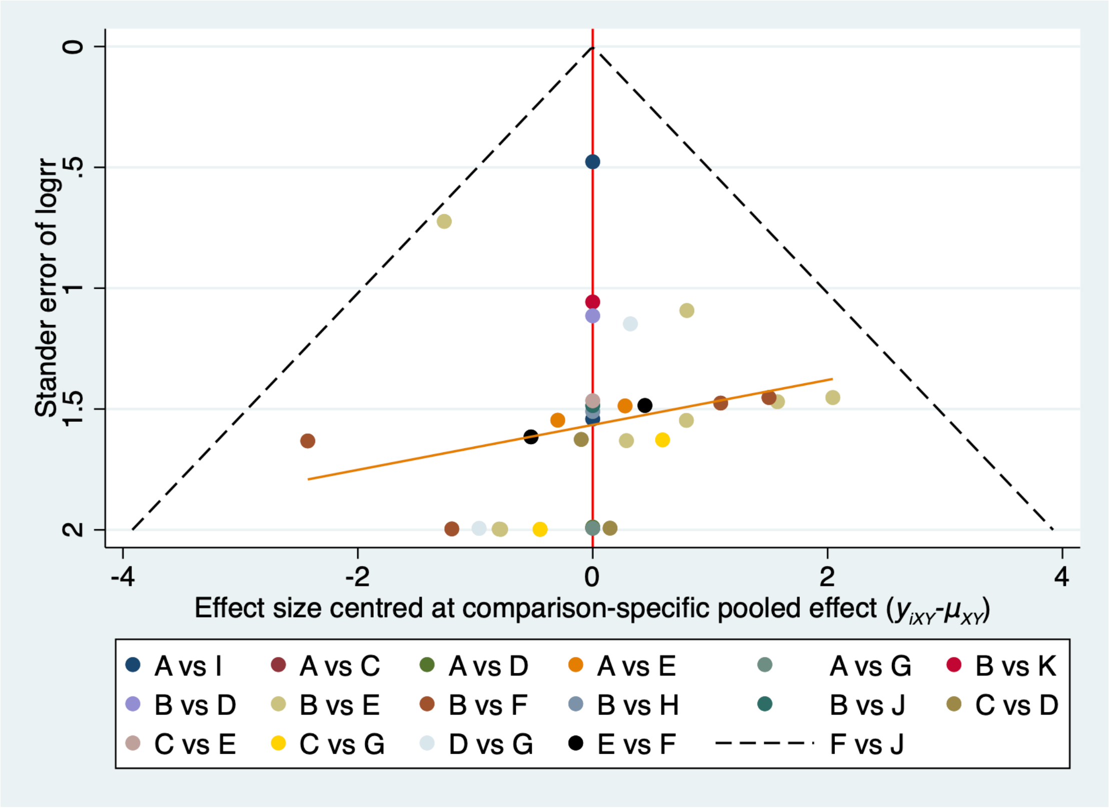

Egger test: P=0.254

A:Placebo; B:Chemo; C:TTD; D:PDL1; E:PD1; F:PD1+Chemo; G:PDL1+TTD; H:PD1+TTD; I:PDL1+CTLA4; J:PD1+CTLA4; K:PDL1+CTLA4+Chemo

(b) head and neck cancer

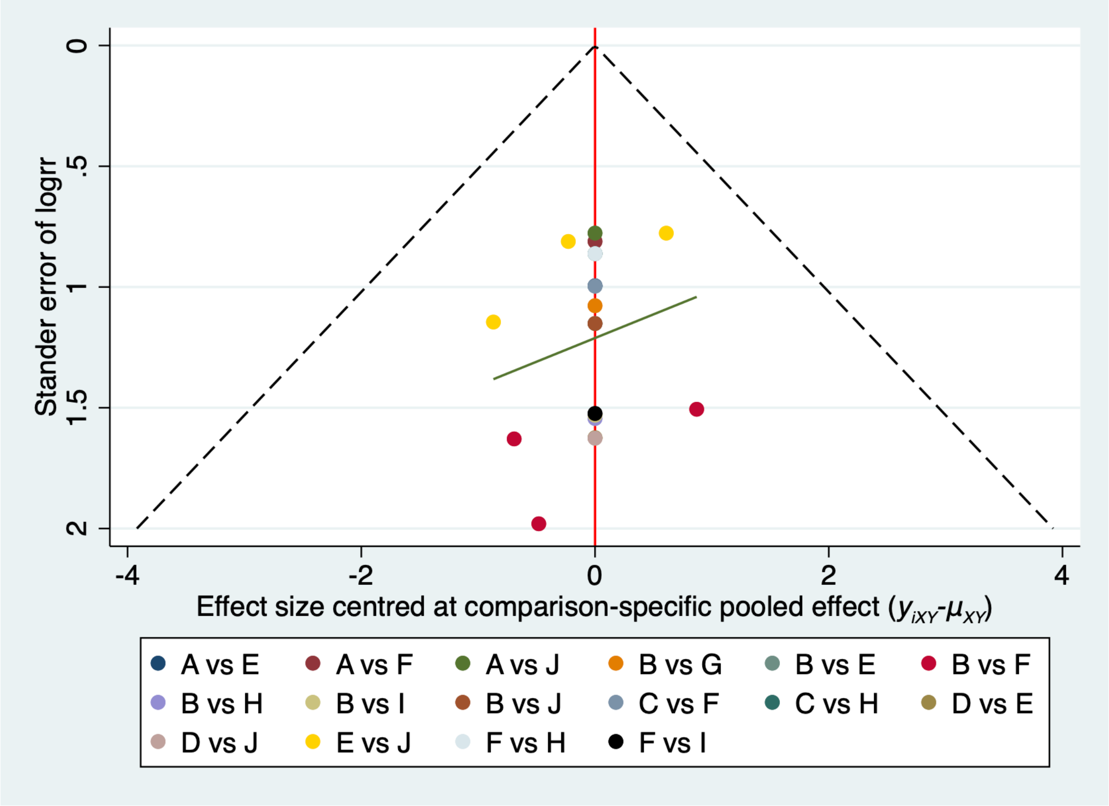

Egger test: P=0.154

A:Placebo; B:Chemo; C:TTD+Chemo; D:CTLA4; E:PDL1; F:PD1; G:PDL1+Chemo; H:PD1+Chemo; I:PD1+TTD; J:PDL1+CTLA4

(c) respiratory system cancer

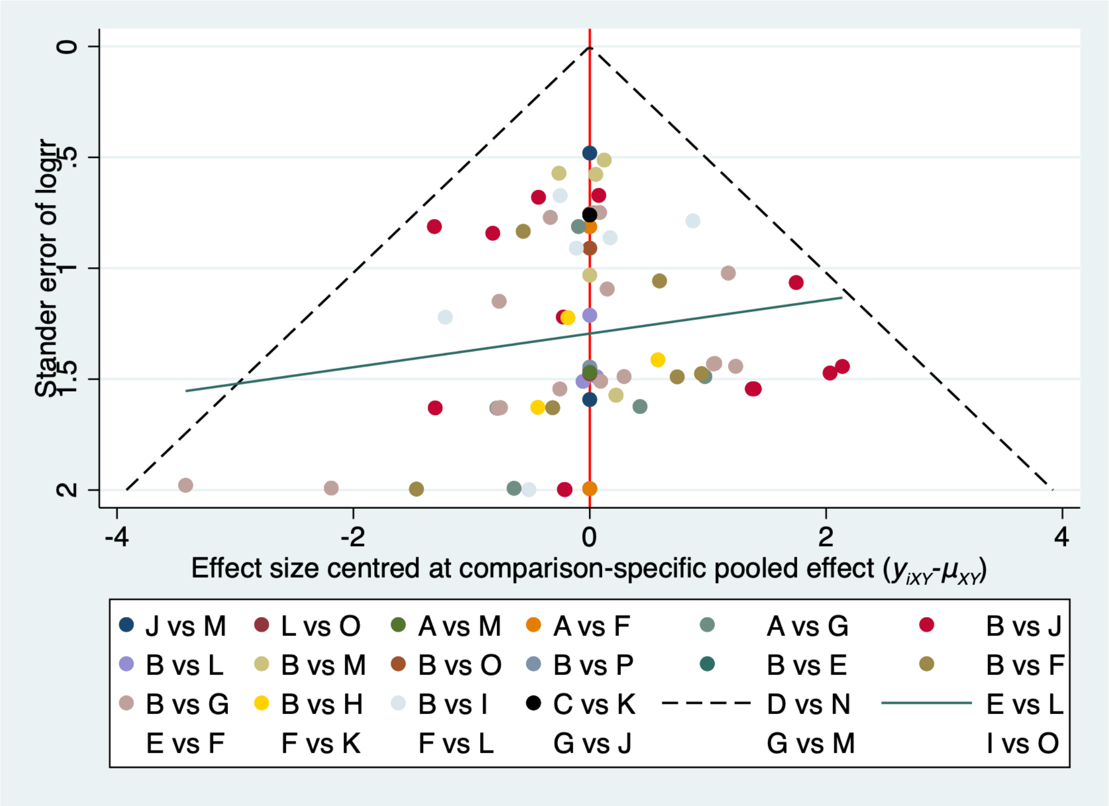

Egger test: P=0.060

A:Placebo; B:Chemo; C:TTD; D:TTD+Chemo; E:CTLA4; F:PDL1; G:PD1; H:CTLA4+Chemo; I:PDL1+Chemo; J:PD1+Chemo; K:PDL1+TTD; L:PDL1+CTLA4; M:PD1+CTLA4; N:PDL1+TTD+Chemo; O:PDL1+CTLA4+Chemo; P:PD1+CTLA4+Chemo

(d) skin cancer

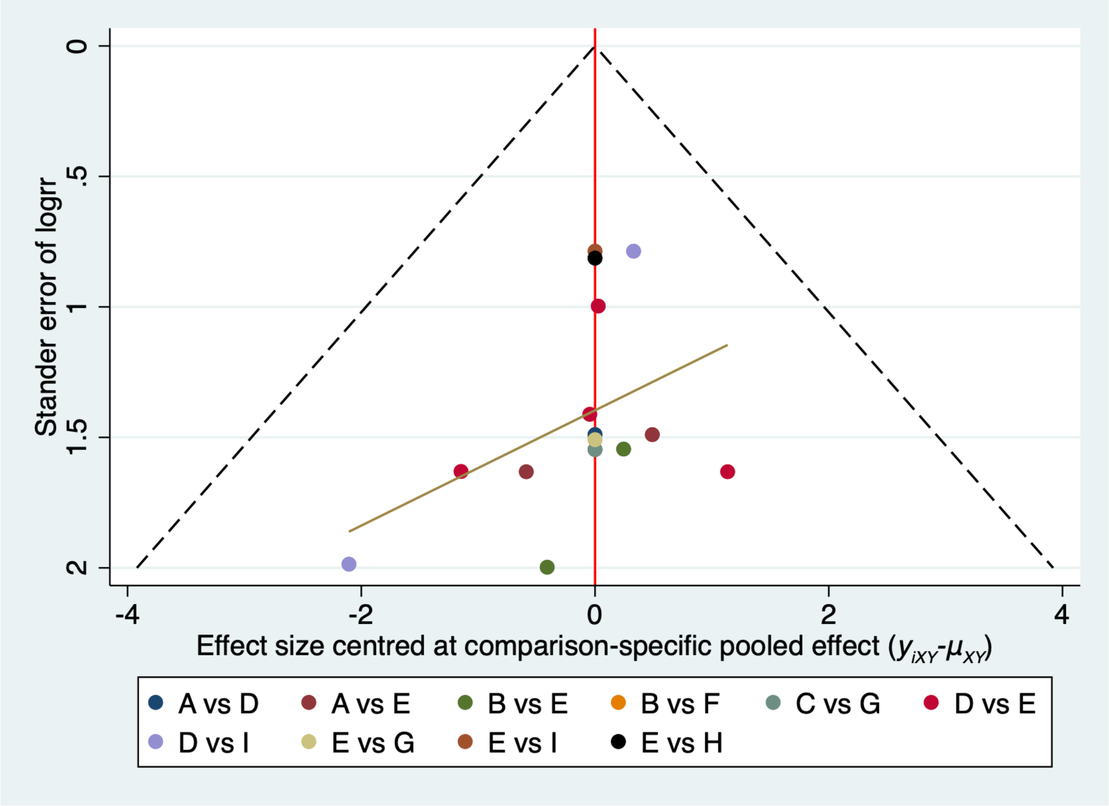

Egger test: P=0.837

A:Placebo; B:Chemo; C:TTD; D:CTLA4; E:PD1; F:CTLA4+Chemo; G:PDL1+TTD; H:PD1+TTD; I:PD1+CTLA4

(e) urogenital system cancer

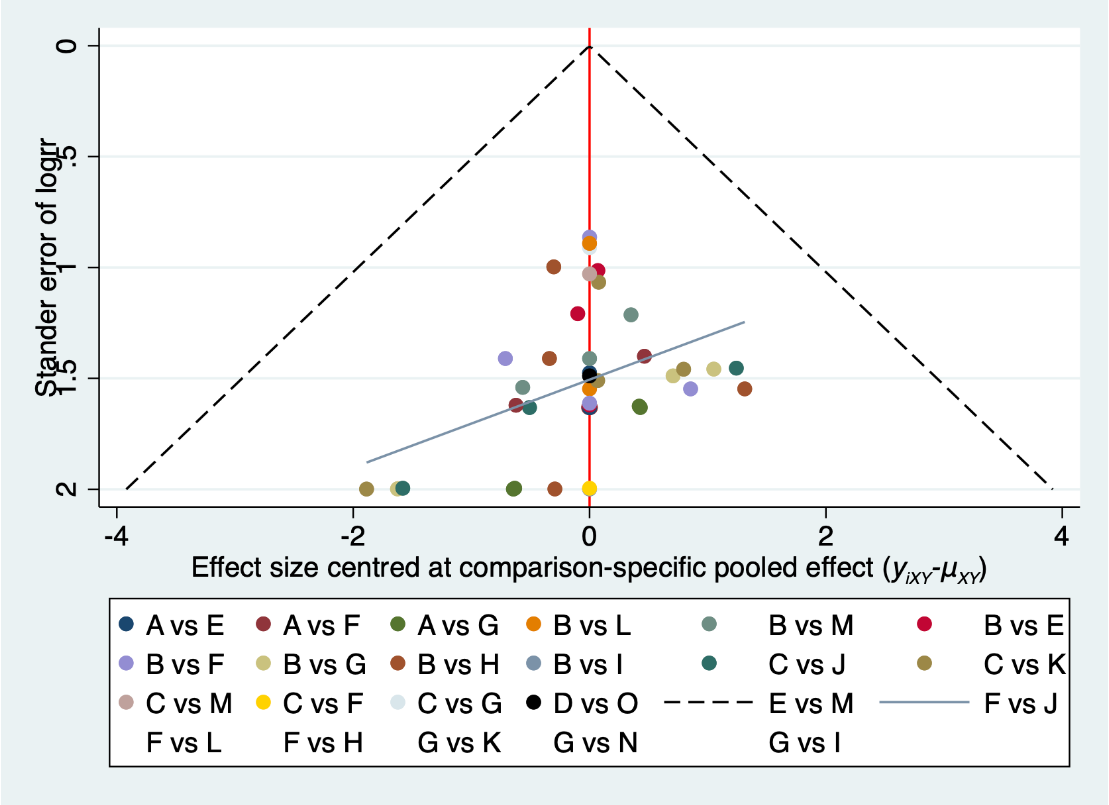

Egger test: P=0.663

A:Placebo; B:Chemo; C:TTD; D:TTD+Chemo; E:CTLA4; F:PDL1; G:PD1; H:CTLA4+Chemo; I:PDL1+Chemo; J:PD1+Chemo; K:PDL1+TTD; L:PD1+TTD; M:PDL1+CTLA4; N:PD1+CTLA4; O:PD1+PDL1; P:PDL1+TTD+Chemo; Q:PDL1+CTLA4+Chemo; R:PD1+CTLA4+Chemo

Abbreviations:PAEs, pulmonary adverse events; TTD, Targeted therapy drug; Chemo, Chemotherapy; PD-1, programmed cell death 1; PD-L1, programmed cell death ligand 1; CTLA4, cytotoxic T-lymphocyte antigen 4.

Figure S4. Network plots for PAEs in patients with (A) respiratory system cancer, (B) urogenital system cancer,(C) skin cancer,(D)head and neck cancer ,(E) digestive system cancer.

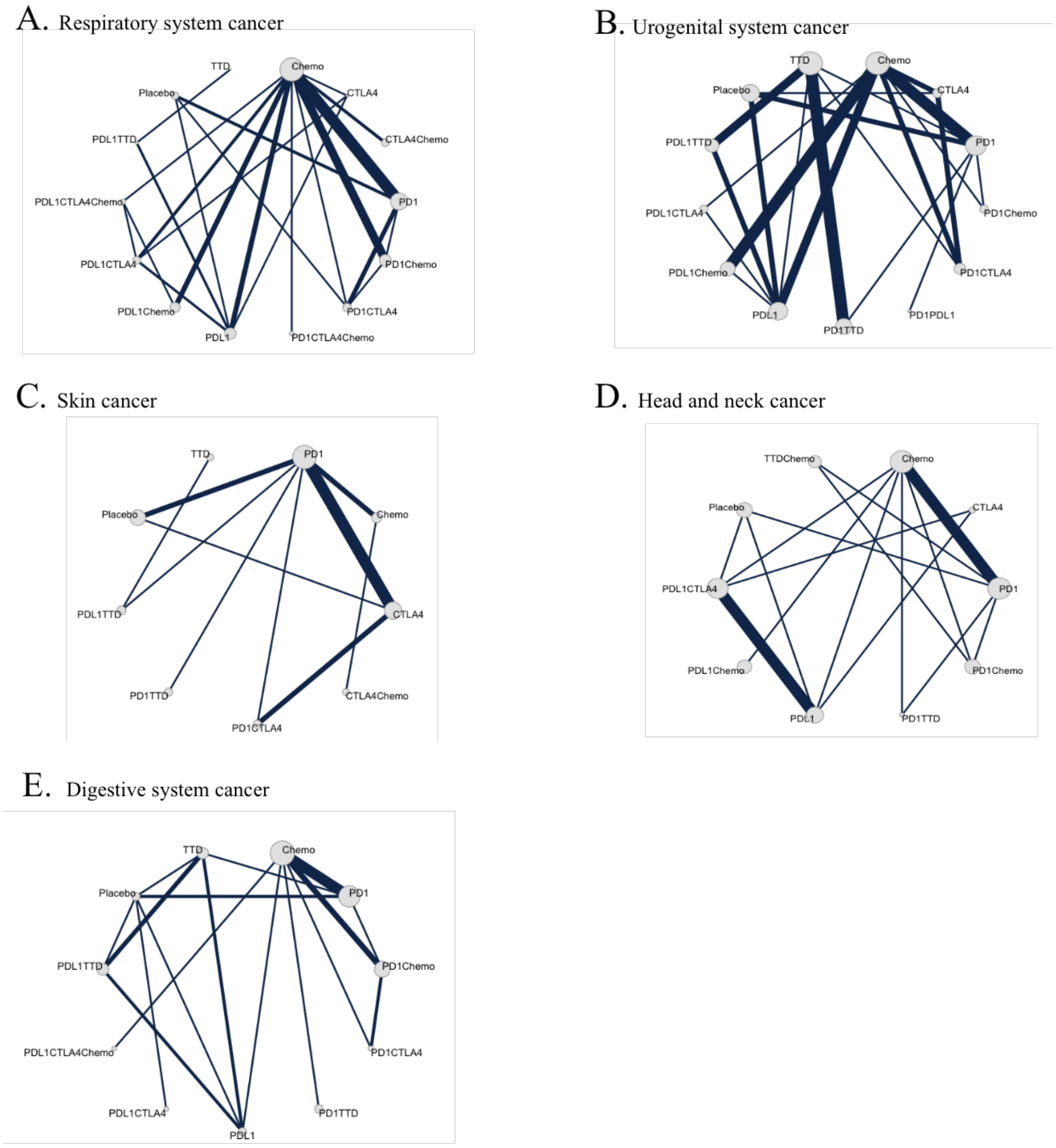

Nodes indicate the classes which are evaluated in clinical trials. Lines represent head-to-head comparisons of the two treatment regimens indicated by the connected nodes. The thickness of lines is weighted according to the number of trials comparing the two connected treatment regimens. The size of the node is proportional to the number of trials evaluating the treatment.

Abbreviations:PAEs, pulmonary adverse events; TTD, Targeted therapy drug; Chemo, Chemotherapy; PD-1, programmed cell death 1; PD-L1, programmed cell death ligand 1; CTLA4, cytotoxic T-lymphocyte antigen 4.

Figure S5 .The distribution of SUCRA values stratified by cancer types for (A)all-grade PAEs,(B)grade 3-4 PAEs.

A. all-grade PAEs

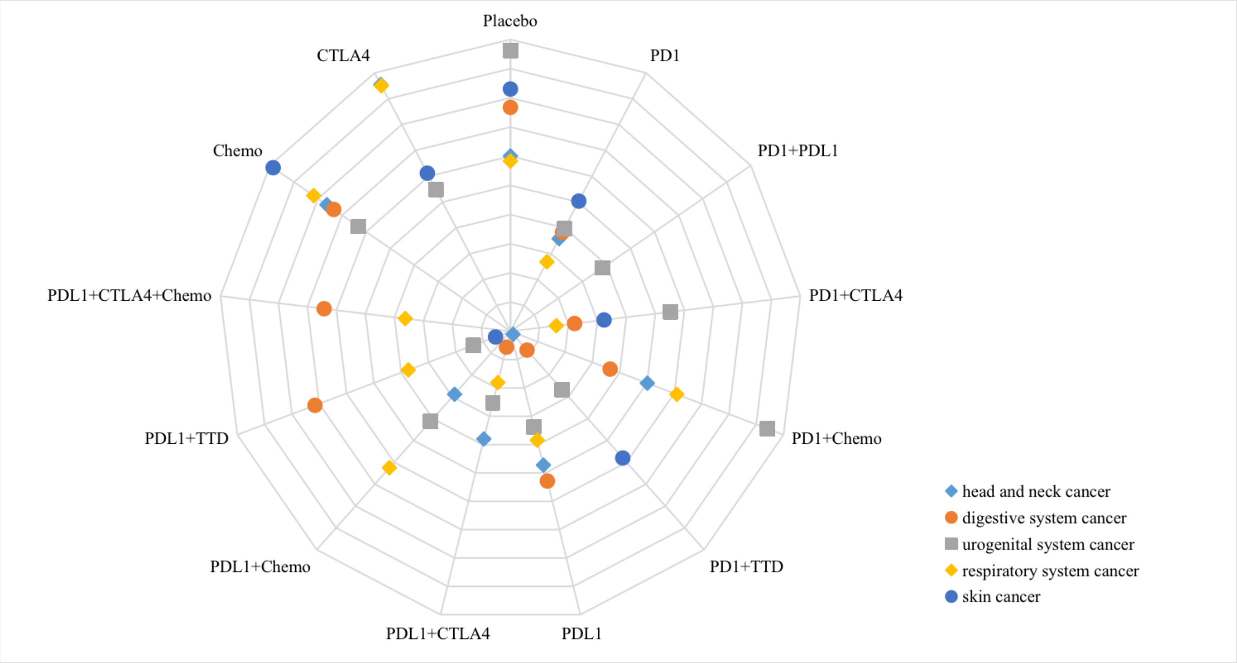

B. Grade 3-4 PAEs

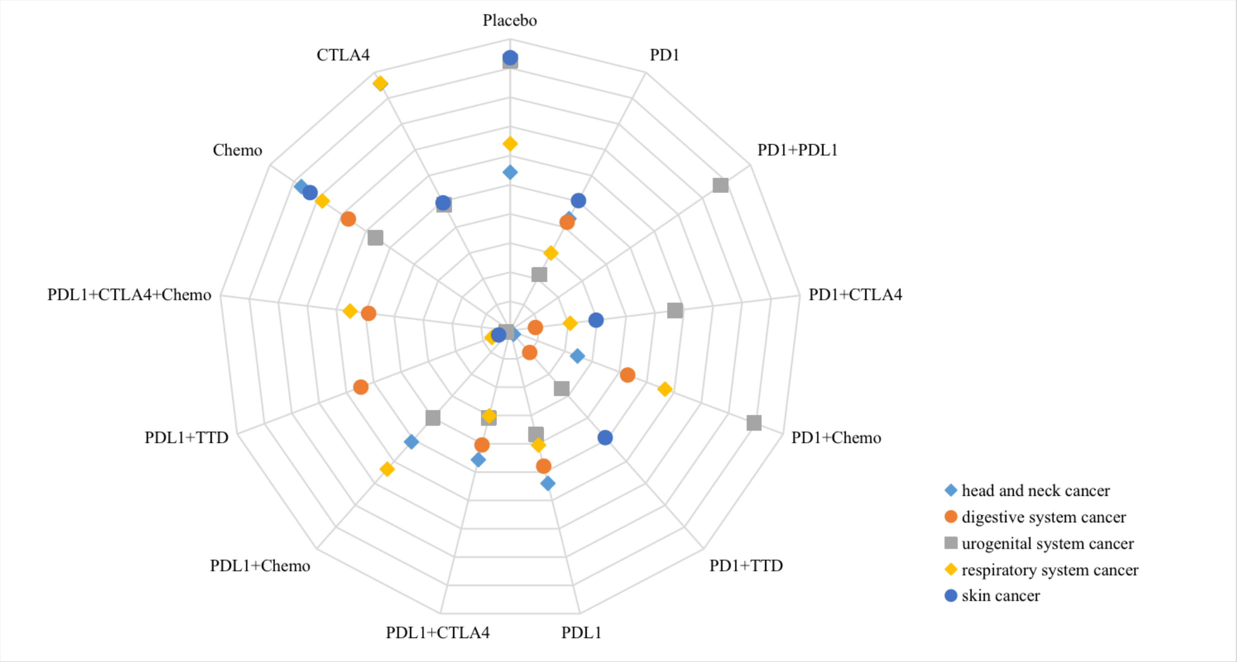

Points closer to the center of the circle represent higher levels of pulmonary toxicity risk.

Abbreviations:PAEs, pulmonary adverse events;TTD, Targeted therapy drug; Chemo, Chemotherapy; PD-1, programmed cell death 1; PD-L1, programmed cell death ligand 1; CTLA4, cytotoxic T-lymphocyte antigen 4;SUCRA, surface under the cumulative ranking.

Figure S6. Subgroup analysis by dose of ICIs for (A)all-grade PAEs;(B) grade 3-4 PAEs.

A. all-grade PAEs

Study or Subgroup	Nivolumab Events	Nivolumab Total	Soc Events	Soc Total	Weight	Odds Ratio M-H, Random, 95% CI
1.1.1 Niv 240mg q2w						
Bajorin,2021	3	351	0	348	7.7%	7.00 [0.36, 136.02]
Fennell,2021	1	221	0	111	6.7%	1.52 [0.06, 37.54]
Hamanishi,2021	3	156	4	155	22.3%	0.74 [0.16, 3.36]
Owonikoko,2021	14	279	5	273	34.5%	2.83 [1.01, 7.97]
Subtotal (95% CI)		1007		887	71.1%	2.01 [0.91, 4.45]
Total events	21		9			
Heterogeneity: Tau² = 0.00; Chi² = 2.82, df = 3 (P = 0.42); I² = 0%						
Test for overall effect: Z = 1.72 (P = 0.09)						
1.1.2 Niv 360mg q3w						
Sugawara,2021	20	273	3	275	28.9%	7.17 [2.10, 24.41]
Subtotal (95% CI)		273		275	28.9%	7.17 [2.10, 24.41]
Total events	20		3			
Heterogeneity: Not applicable						
Test for overall effect: Z = 3.15 (P = 0.002)						
Total (95% CI)		1280		1162	100.0%	2.82 [1.18, 6.77]
Total events	41		12			
Heterogeneity: Tau² = 0.30; Chi² = 5.80, df = 4 (P = 0.21); I² = 31%						
Test for overall effect: Z = 2.33 (P = 0.02)						
Test for subgroup differences: Chi² = 2.91, df = 1 (P = 0.09), I² = 65.7%						

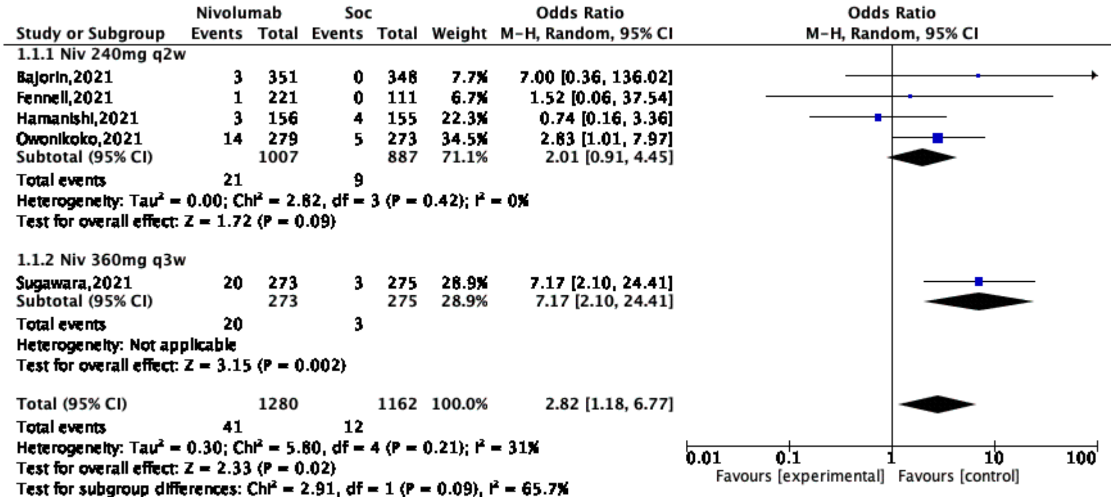

Study or Subgroup	Ate+Chemo Events	Ate+Chemo Total	Chemo Events	Chemo Total	Weight	Odds Ratio M-H, Fixed, 95% CI
1.2.1 Ate 840mg q2w+Chemo						
Emens,2021	1	460	0	430	6.1%	2.81 [0.11, 69.18]
Subtotal (95% CI)		460		430	6.1%	2.81 [0.11, 69.18]
Total events	1		0			
Heterogeneity: Not applicable						
Test for overall effect: Z = 0.63 (P = 0.53)						
1.2.2 Ate 1200mg q3w+Chemo						
Horn,2018	3	198	4	196	47.1%	0.74 [0.16, 3.34]
West,2019	10	473	3	232	46.8%	1.65 [0.45, 6.05]
Subtotal (95% CI)		671		428	93.9%	1.19 [0.46, 3.09]
Total events	13		7			
Heterogeneity: Chi² = 0.63, df = 1 (P = 0.43); I² = 0%						
Test for overall effect: Z = 0.36 (P = 0.72)						
Total (95% CI)		1131		858	100.0%	1.29 [0.52, 3.21]
Total events	14		7			
Heterogeneity: Chi² = 0.89, df = 2 (P = 0.64); I² = 0%						
Test for overall effect: Z = 0.55 (P = 0.58)						
Test for subgroup differences: Chi² = 0.25, df = 1 (P = 0.62), I² = 0%						

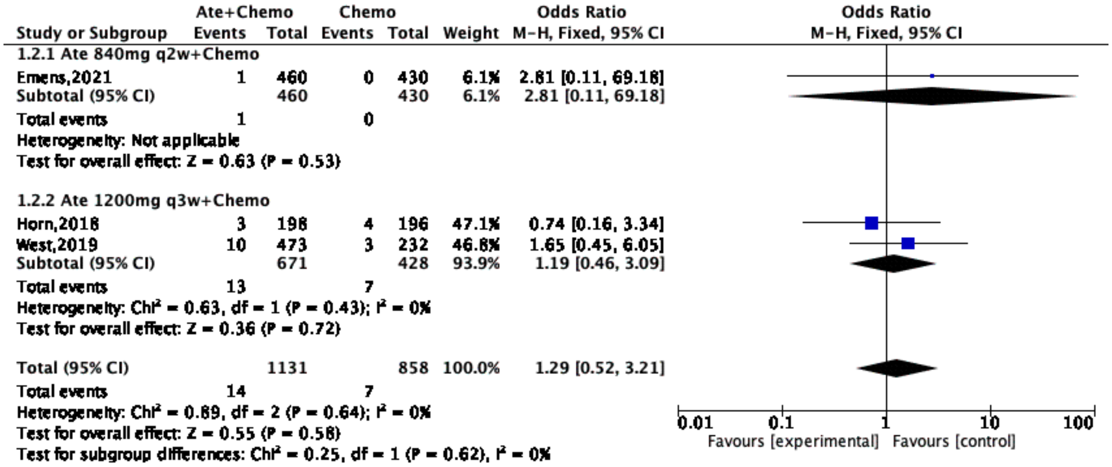

Study or Subgroup	Ate+TTD Events	Ate+TTD Total	TTD Events	TTD Total	Weight	Odds Ratio M-H, Fixed, 95% CI
1.3.1 Ate 840mg q2w+TTD						
Eng,2019	1	179	0	80	5.8%	1.35 [0.05, 33.57]
Gutzmer,2020	22	230	13	281	90.0%	2.18 [1.07, 4.43]
Subtotal (95% CI)		409		361	95.8%	2.13 [1.06, 4.26]
Total events	23		13			
Heterogeneity: Chi² = 0.08, df = 1 (P = 0.78); I² = 0%						
Test for overall effect: Z = 2.14 (P = 0.03)						
1.3.2 Ate 1200mg q3w+TTD						
McDermott,2018	0	101	0	101		Not estimable
Rini,2019	8	451	0	446	4.2%	17.11 [0.98, 297.42]
Subtotal (95% CI)		552		547	4.2%	17.11 [0.98, 297.42]
Total events	8		0			
Heterogeneity: Not applicable						
Test for overall effect: Z = 1.95 (P = 0.05)						
Total (95% CI)		961		908	100.0%	2.76 [1.44, 5.30]
Total events	31		13			
Heterogeneity: Chi² = 2.18, df = 2 (P = 0.34); I² = 8%						
Test for overall effect: Z = 3.05 (P = 0.002)						
Test for subgroup differences: Chi² = 1.93, df = 1 (P = 0.16), I² = 48.2%						

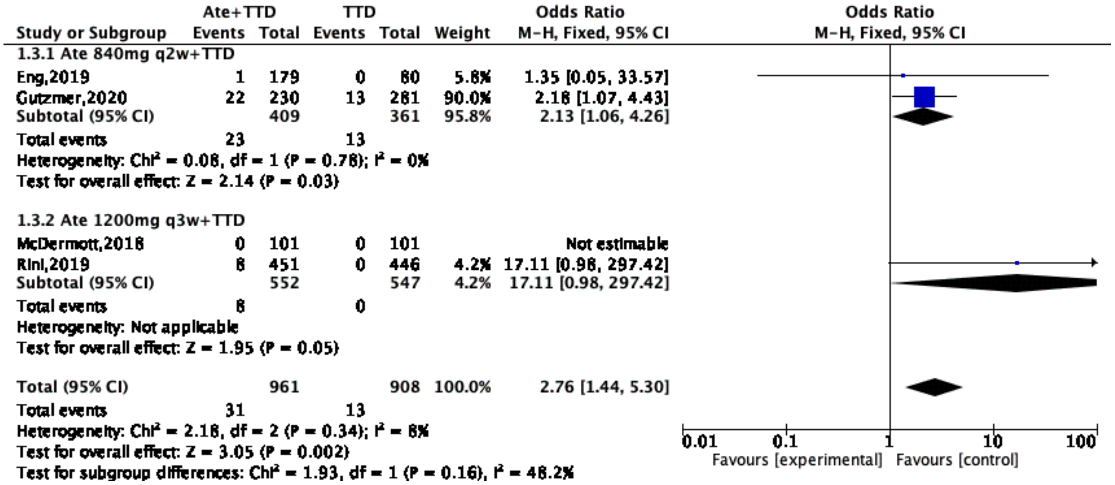

Study or Subgroup	Dur Events	Dur Total	Dur+Tre Events	Dur+Tre Total	Weight	Odds Ratio M-H, Fixed, 95% CI
**1.4.1 Dur 20ma/kg q4w**						
Rizvi,2020	6	369	15	371	61.6%	0.39 [0.15, 1.02]
Subtotal (95% CI)		369		371	61.6%	0.39 [0.15, 1.02]
Total events	6		15			
Heterogeneity: Not applicable						
Test for overall effect: Z = 1.91 (P = 0.06)						
**1.4.2 Dur 10mg/kg q2w**						
Ferris,2020	3	237	3	246	12.2%	1.04 [0.21, 5.20]
NCT02319044	1	67	1	133	2.8%	2.00 [0.12, 32.48]
Planchard,2020	1	117	7	173	23.4%	0.20 [0.02, 1.68]
Subtotal (95% CI)		421		552	38.4%	0.60 [0.20, 1.75]
Total events	5		11			
Heterogeneity: Chi^2 = 2.17, df = 2 (P = 0.34); I^2 = 8%						
Test for overall effect: Z = 0.94 (P = 0.35)						
**Total (95% CI)**		790		923	100.0%	0.47 [0.23, 0.96]
Total events	11		26			
Heterogeneity: Chi^2 = 2.70, df = 3 (P = 0.44); I^2 = 0%						
Test for overall effect: Z = 2.07 (P = 0.04)						
Test for subgroup differences: Chi^2 = 0.33, df = 1 (P = 0.57), I^2 = 0%						

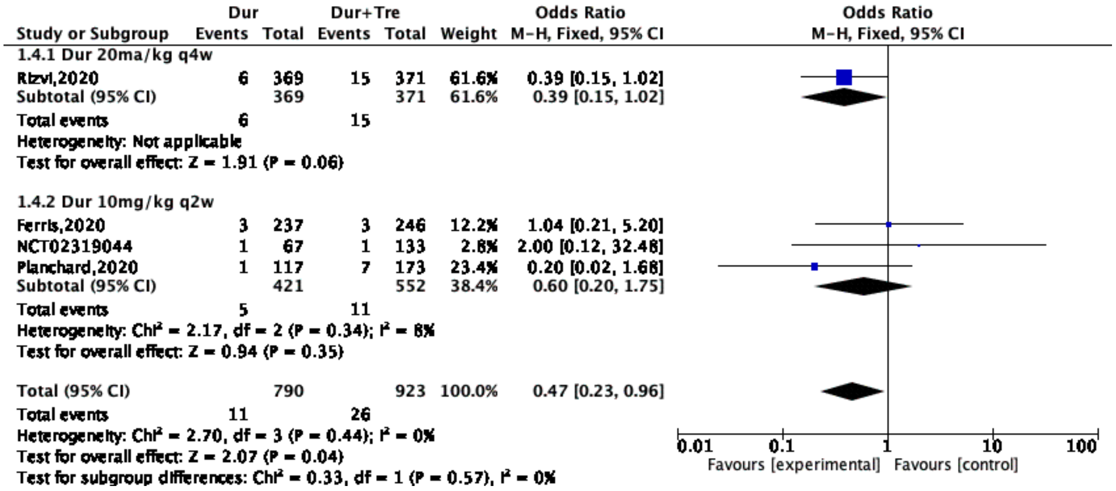

Study or Subgroup	Dur Events	Dur Total	Placebo Events	Placebo Total	Weight	Odds Ratio M-H, Random, 95% CI
**1.5.1 Dur 750mg q2w**						
Loibl,2019	1	92	1	82	33.3%	0.89 [0.05, 14.46]
Subtotal (95% CI)		92		82	33.3%	0.89 [0.05, 14.46]
Total events	1		1			
Heterogeneity: Not applicable						
Test for overall effect: Z = 0.08 (P = 0.93)						
**1.5.2 Dur 1500mg q4w**						
NCT02551159	2	202	2	196	66.7%	0.97 [0.14, 6.95]
Subtotal (95% CI)		202		196	66.7%	0.97 [0.14, 6.95]
Total events	2		2			
Heterogeneity: Not applicable						
Test for overall effect: Z = 0.03 (P = 0.98)						
**Total (95% CI)**		294		278	100.0%	0.94 [0.19, 4.71]
Total events	3		3			
Heterogeneity: Tau^2 = 0.00; Chi^2 = 0.00, df = 1 (P = 0.96); I^2 = 0%						
Test for overall effect: Z = 0.07 (P = 0.94)						
Test for subgroup differences: Chi^2 = 0.00, df = 1 (P = 0.96), I^2 = 0%						

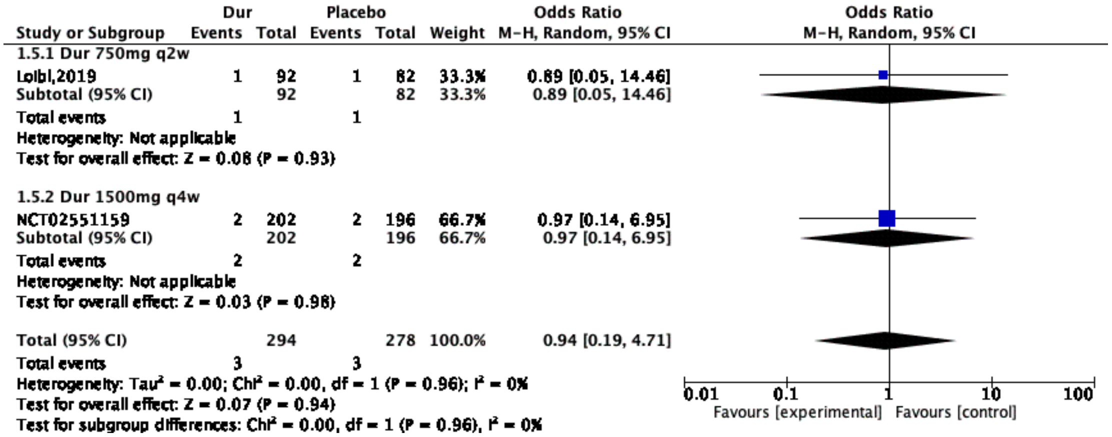

Study or Subgroup	Pem Events	Pem Total	Ipi Events	Ipi Total	Weight	Odds Ratio M-H, Fixed, 95% CI
**1.6.1 Pem 10mg/kg q2w**						
Robert2,2015	1	278	1	256	50.4%	0.92 [0.06, 14.79]
Subtotal (95% CI)		278		256	50.4%	0.92 [0.06, 14.79]
Total events	1		1			
Heterogeneity: Not applicable						
Test for overall effect: Z = 0.06 (P = 0.95)						
**1.6.2 Pem 10mg/kg q3w**						
Robert1,2015	5	277	1	256	49.6%	4.69 [0.54, 40.40]
Subtotal (95% CI)		277		256	49.6%	4.69 [0.54, 40.40]
Total events	5		1			
Heterogeneity: Not applicable						
Test for overall effect: Z = 1.41 (P = 0.16)						
**Total (95% CI)**		555		512	100.0%	2.79 [0.56, 13.86]
Total events	6		2			
Heterogeneity: Chi^2 = 0.84, df = 1 (P = 0.36); I^2 = 0%						
Test for overall effect: Z = 1.25 (P = 0.21)						
Test for subgroup differences: Chi^2 = 0.82, df = 1 (P = 0.36), I^2 = 0%						

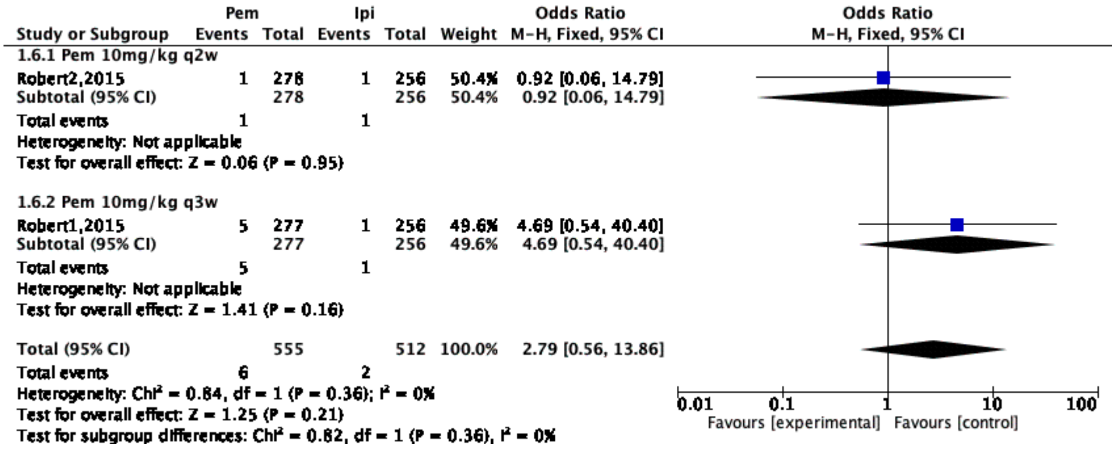

Study or Subgroup	Pem Events	Pem Total	Chemo Events	Chemo Total	Weight	Odds Ratio M-H, Fixed, 95% CI
**1.7.1 Pem 2mg/kg q3w**						
NCT01905657	24	339	3	310	49.3%	7.80 [2.32, 26.16]
Subtotal (95% CI)		339		310	49.3%	7.80 [2.32, 26.16]
Total events	24		3			
Heterogeneity: Not applicable						
Test for overall effect: Z = 3.33 (P = 0.0009)						
**1.7.2 Pem 10mg/kg q3w**						
NCT01905657	18	344	3	310	50.7%	5.65 [1.65, 19.37]
Subtotal (95% CI)		344		310	50.7%	5.65 [1.65, 19.37]
Total events	18		3			
Heterogeneity: Not applicable						
Test for overall effect: Z = 2.75 (P = 0.006)						
**Total (95% CI)**		683		620	100.0%	6.71 [2.83, 15.89]
Total events	42		6			
Heterogeneity: Chi^2 = 0.13, df = 1 (P = 0.71); I^2 = 0%						
Test for overall effect: Z = 4.33 (P < 0.0001)						
Test for subgroup differences: Chi^2 = 0.13, df = 1 (P = 0.71), I^2 = 0%						

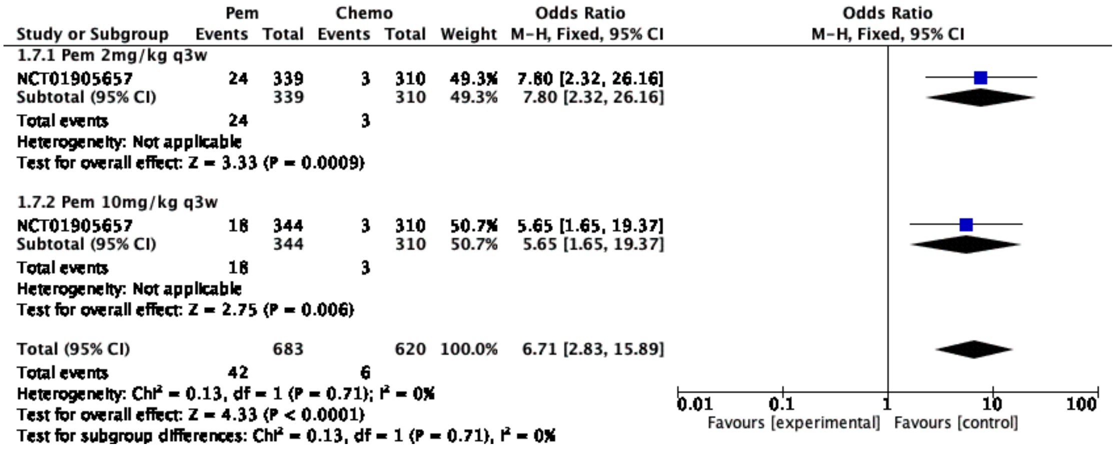

Study or Subgroup	Ipi Events	Ipi Total	PD1 Events	PD1 Total	Weight	Odds Ratio M-H, Fixed, 95% CI
1.8.1 Ipi 10mg/kg q3w						
Ascierto,2020	1	453	2	452	20.6%	0.50 [0.04, 5.51]
Subtotal (95% CI)		453		452	20.6%	0.50 [0.04, 5.51]
Total events	1		2			
Heterogeneity: Not applicable						
Test for overall effect: Z = 0.57 (P = 0.57)						
1.8.2 Ipi 3mg/kg q3w						
Hodi,2018	2	311	2	313	20.4%	1.01 [0.14, 7.19]
Robert1,2015	1	256	5	277	49.2%	0.21 [0.02, 1.84]
Robert2,2015	1	256	1	278	9.8%	1.09 [0.07, 17.46]
Subtotal (95% CI)		823		868	79.4%	0.52 [0.16, 1.75]
Total events	4		8			
Heterogeneity: Chi^2 = 1.36, df = 2 (P = 0.51); I^2 = 0%						
Test for overall effect: Z = 1.05 (P = 0.29)						
Total (95% CI)		1276		1320	100.0%	0.52 [0.18, 1.53]
Total events	5		10			
Heterogeneity: Chi^2 = 1.36, df = 3 (P = 0.71); I^2 = 0%						
Test for overall effect: Z = 1.19 (P = 0.23)						
Test for subgroup differences: Chi^2 = 0.00, df = 1 (P = 0.97), I^2 = 0%						

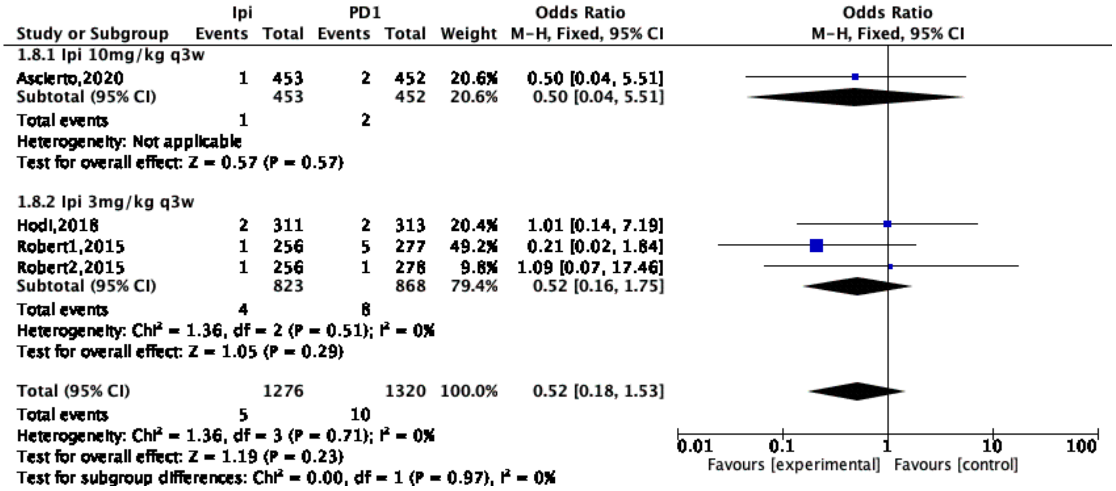

Study or Subgroup	Niv+Ipi Events	Niv+Ipi Total	Niv Events	Niv Total	Weight	Odds Ratio M-H, Fixed, 95% CI
1.9.1 Niv 1mg/kg q3w+Ipi3 mg/kg q3w						
Hodi,2018	8	313	2	313	25.9%	4.08 [0.86, 19.36]
Subtotal (95% CI)		313		313	25.9%	4.08 [0.86, 19.36]
Total events	8		2			
Heterogeneity: Not applicable						
Test for overall effect: Z = 1.77 (P = 0.08)						
1.9.2 Niv 3mg/kg q2w+Ipi 1mg/kg q6w						
Gettinger,2021	9	124	6	123	74.1%	1.53 [0.53, 4.43]
Subtotal (95% CI)		124		123	74.1%	1.53 [0.53, 4.43]
Total events	9		6			
Heterogeneity: Not applicable						
Test for overall effect: Z = 0.78 (P = 0.44)						
Total (95% CI)		437		436	100.0%	2.19 [0.93, 5.16]
Total events	17		8			
Heterogeneity: Chi^2 = 1.05, df = 1 (P = 0.30); I^2 = 5%						
Test for overall effect: Z = 1.79 (P = 0.07)						
Test for subgroup differences: Chi^2 = 1.04, df = 1 (P = 0.31), I^2 = 4.1%						

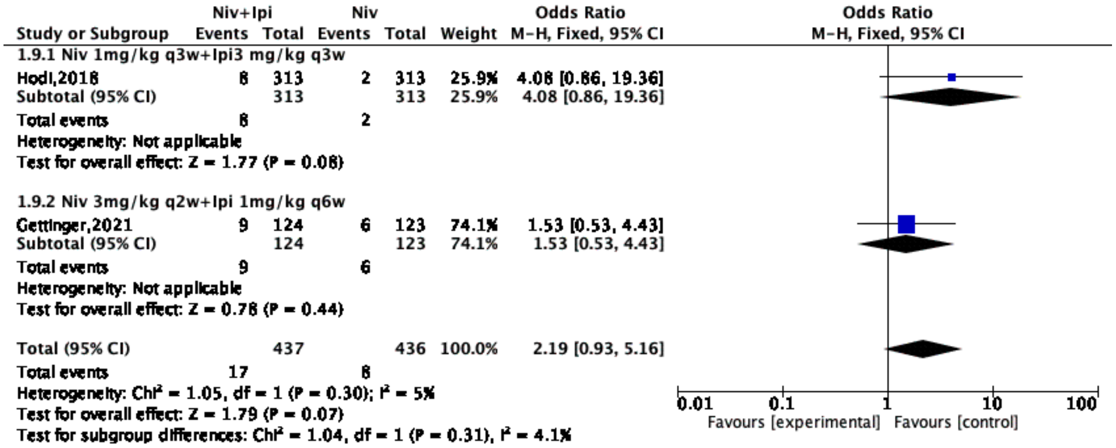

Study or Subgroup	Niv+Ipi Events	Niv+Ipi Total	Ipi Events	Ipi Total	Weight	Odds Ratio M-H, Fixed, 95% CI
1.10.1 Niv 1mg/kg+Ipi3 mg/kg						
Hodi,2018	8	313	2	313	38.0%	4.08 [0.86, 19.36]
NCT03033576	4	68	0	23	13.5%	3.28 [0.17, 63.26]
Subtotal (95% CI)		381		336	51.5%	3.87 [0.97, 15.45]
Total events	12		2			
Heterogeneity: Chi^2 = 0.02, df = 1 (P = 0.90); I^2 = 0%						
Test for overall effect: Z = 1.91 (P = 0.06)						
1.10.2 Niv 3mg/kg+Ipi 1mg/kg						
NCT02985957	4	73	2	38	48.5%	1.04 [0.18, 5.97]
Subtotal (95% CI)		73		38	48.5%	1.04 [0.18, 5.97]
Total events	4		2			
Heterogeneity: Not applicable						
Test for overall effect: Z = 0.05 (P = 0.96)						
Total (95% CI)		454		374	100.0%	2.50 [0.87, 7.19]
Total events	16		4			
Heterogeneity: Chi^2 = 1.38, df = 2 (P = 0.50); I^2 = 0%						
Test for overall effect: Z = 1.70 (P = 0.09)						
Test for subgroup differences: Chi^2 = 1.33, df = 1 (P = 0.25), I^2 = 24.8%						

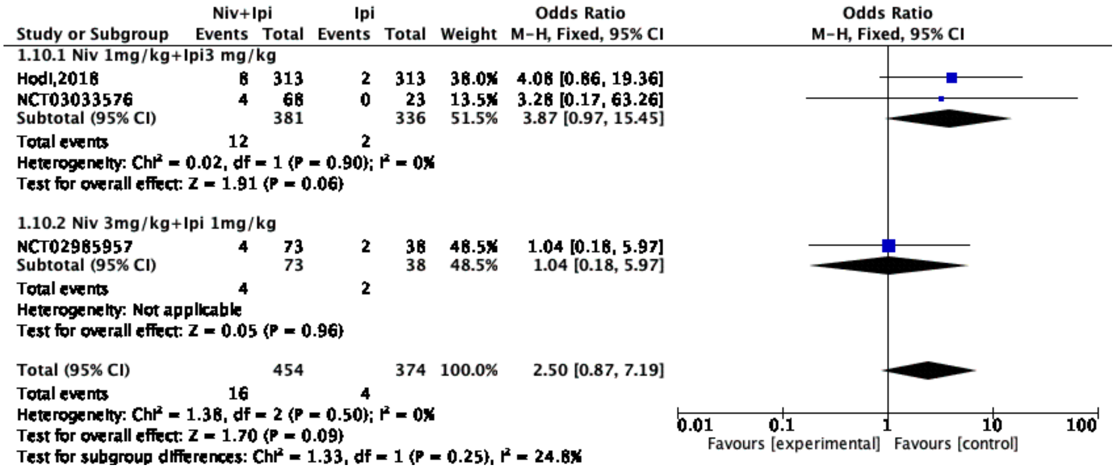

B. Grade 3-4 PAEs

Study or Subgroup	Nivolumab Events	Nivolumab Total	Soc Events	Soc Total	Weight	Odds Ratio M-H, Random, 95% CI
**1.1.1 Niv 240mg q2w**						
Bajorin,2021	1	351	0	348	14.0%	2.98 [0.12, 73.47]
Fennell,2021	1	221	0	111	13.9%	1.52 [0.06, 37.54]
Hamanishi,2021	0	156	0	155		Not estimable
Owonikoko,2021	4	279	0	273	16.7%	8.93 [0.48, 166.75]
Subtotal (95% CI)		1007		887	44.6%	3.65 [0.61, 21.88]
Total events	6		0			
Heterogeneity: $Tau^2 = 0.00$; $Chi^2 = 0.69$, df = 2 (P = 0.71); $I^2 = 0\%$						
Test for overall effect: Z = 1.41 (P = 0.16)						
**1.1.2 Niv 360mg q3w**						
Sugawara,2021	6	273	2	275	55.4%	3.07 [0.61, 15.33]
Subtotal (95% CI)		273		275	55.4%	3.07 [0.61, 15.33]
Total events	6		2			
Heterogeneity: Not applicable						
Test for overall effect: Z = 1.37 (P = 0.17)						
Total (95% CI)		1280		1162	100.0%	3.31 [1.00, 10.97]
Total events	12		2			
Heterogeneity: $Tau^2 = 0.00$; $Chi^2 = 0.70$, df = 3 (P = 0.87); $I^2 = 0\%$						
Test for overall effect: Z = 1.96 (P = 0.05)						
Test for subgroup differences: $Chi^2 = 0.02$, df = 1 (P = 0.89), $I^2 = 0\%$						

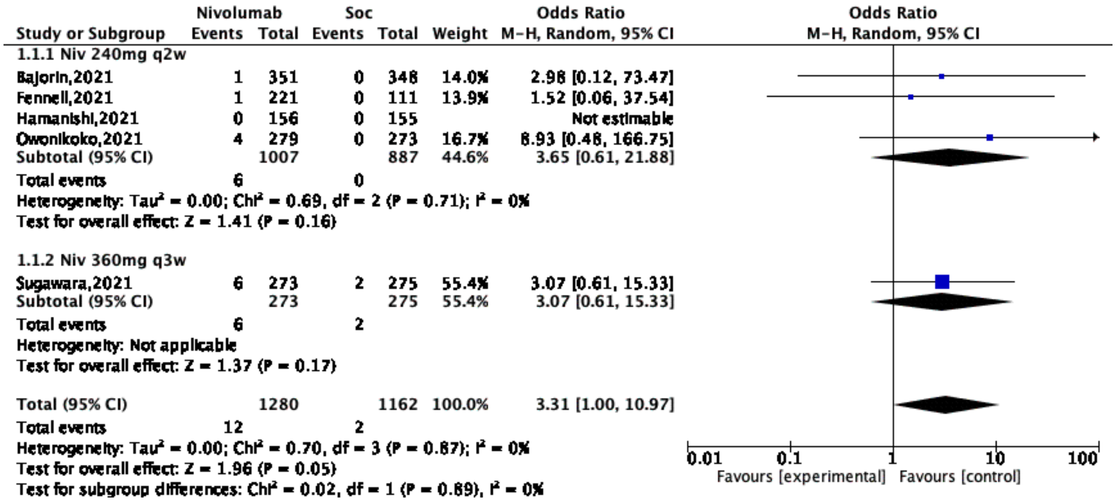

Study or Subgroup	Ate+Chemo Events	Ate+Chemo Total	Chemo Events	Chemo Total	Weight	Odds Ratio M-H, Fixed, 95% CI
**1.2.1 Ate 840mg q2w+Chemo**						
Emens,2021	1	460	0	430	8.0%	2.81 [0.11, 69.18]
Subtotal (95% CI)		460		430	8.0%	2.81 [0.11, 69.18]
Total events	1		0			
Heterogeneity: Not applicable						
Test for overall effect: Z = 0.63 (P = 0.53)						
**1.2.2 Ate 1200mg q3w+Chemo**						
Horn,2018	1	198	2	196	30.9%	0.49 [0.04, 5.47]
West,2019	8	473	3	232	61.1%	1.31 [0.35, 5.00]
Subtotal (95% CI)		671		428	92.0%	1.04 [0.34, 3.21]
Total events	9		5			
Heterogeneity: $Chi^2 = 0.49$, df = 1 (P = 0.49); $I^2 = 0\%$						
Test for overall effect: Z = 0.06 (P = 0.95)						
Total (95% CI)		1131		858	100.0%	1.18 [0.41, 3.39]
Total events	10		5			
Heterogeneity: $Chi^2 = 0.81$, df = 2 (P = 0.67); $I^2 = 0\%$						
Test for overall effect: Z = 0.31 (P = 0.76)						
Test for subgroup differences: $Chi^2 = 0.33$, df = 1 (P = 0.57), $I^2 = 0\%$						

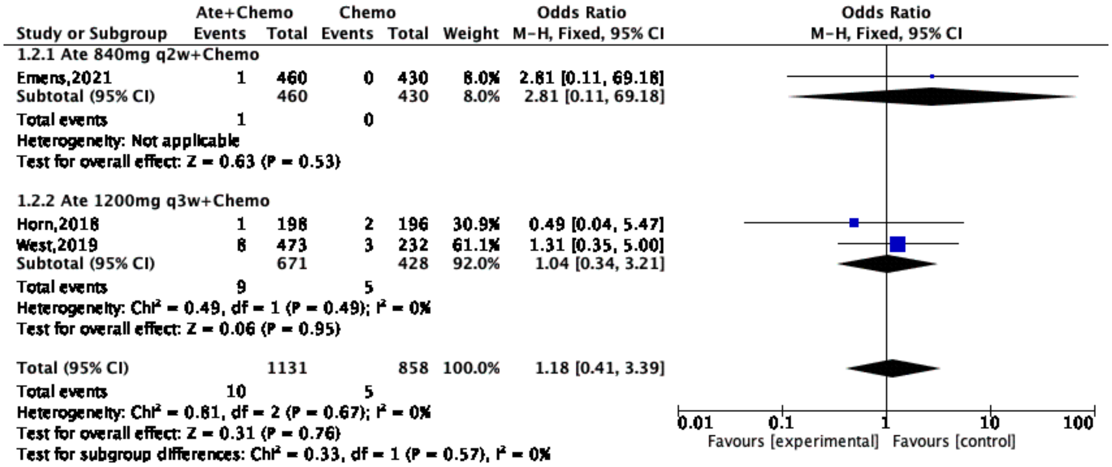

Study or Subgroup	Ate+TTD Events	Ate+TTD Total	TTD Events	TTD Total	Weight	Odds Ratio M-H, Fixed, 95% CI
**1.3.1 Ate 840mg q2w+TTD**						
Eng,2019	1	179	0	80	42.1%	1.35 [0.05, 33.57]
Gutzmer,2020	2	230	0	281	27.5%	6.16 [0.29, 128.95]
Subtotal (95% CI)		409		361	69.6%	3.25 [0.37, 28.26]
Total events	3		0			
Heterogeneity: $Chi^2 = 0.46$, df = 1 (P = 0.50); $I^2 = 0\%$						
Test for overall effect: Z = 1.07 (P = 0.29)						
**1.3.2 Ate 1200mg q3w+TTD**						
McDermott,2018	0	101	0	101		Not estimable
Rini,2019	8	451	0	446	30.4%	17.11 [0.98, 297.42]
Subtotal (95% CI)		552		547	30.4%	17.11 [0.98, 297.42]
Total events	8		0			
Heterogeneity: Not applicable						
Test for overall effect: Z = 1.95 (P = 0.05)						
Total (95% CI)		961		908	100.0%	7.46 [1.40, 39.86]
Total events	11		0			
Heterogeneity: $Chi^2 = 1.43$, df = 2 (P = 0.49); $I^2 = 0\%$						
Test for overall effect: Z = 2.35 (P = 0.02)						
Test for subgroup differences: $Chi^2 = 0.83$, df = 1 (P = 0.36), $I^2 = 0\%$						

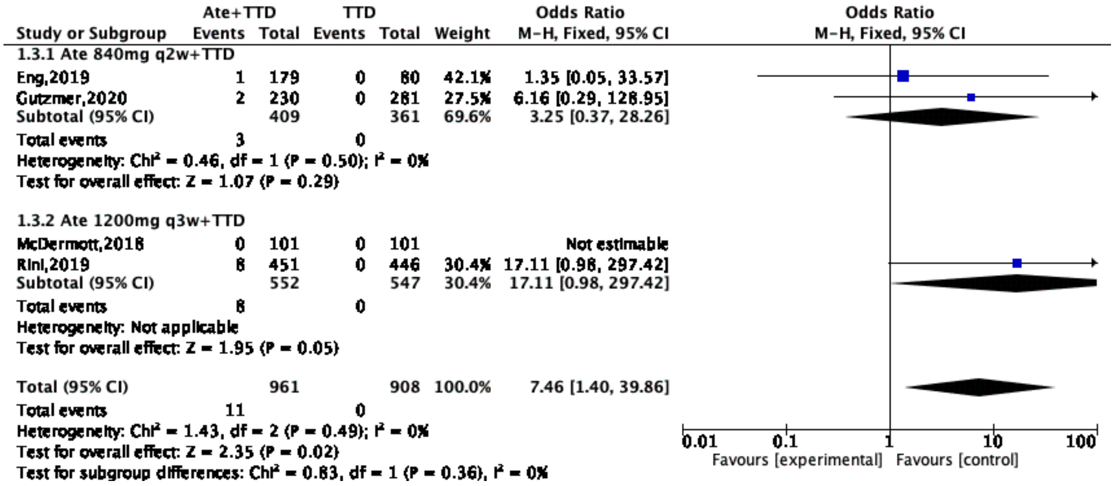

Study or Subgroup	Dur Events	Dur Total	Dur+Tre Events	Dur+Tre Total	Weight	Odds Ratio M-H, Fixed, 95% CI
1.4.1 Dur 20ma/kg q4w						
Rizvi,2020	5	369	3	371	27.0%	1.68 [0.40, 7.10]
Subtotal (95% CI)		369		371	27.0%	1.68 [0.40, 7.10]
Total events	5		3			
Heterogeneity: Not applicable						
Test for overall effect: Z = 0.71 (P = 0.48)						
1.4.2 Dur 10mg/kg q2w						
Ferris,2020	3	237	3	246	26.6%	1.04 [0.21, 5.20]
NCT02319044	1	67	1	133	6.0%	2.00 [0.12, 32.48]
Planchard,2020	0	117	5	173	40.4%	0.13 [0.01, 2.38]
Subtotal (95% CI)		421		552	73.0%	0.61 [0.20, 1.92]
Total events	4		9			
Heterogeneity: Chi² = 2.19, df = 2 (P = 0.33); I² = 9%						
Test for overall effect: Z = 0.84 (P = 0.40)						
Total (95% CI)		790		923	100.0%	0.90 [0.38, 2.14]
Total events	9		12			
Heterogeneity: Chi² = 2.77, df = 3 (P = 0.43); I² = 0%						
Test for overall effect: Z = 0.23 (P = 0.82)						
Test for subgroup differences: Chi² = 1.16, df = 1 (P = 0.28), I² = 13.7%						

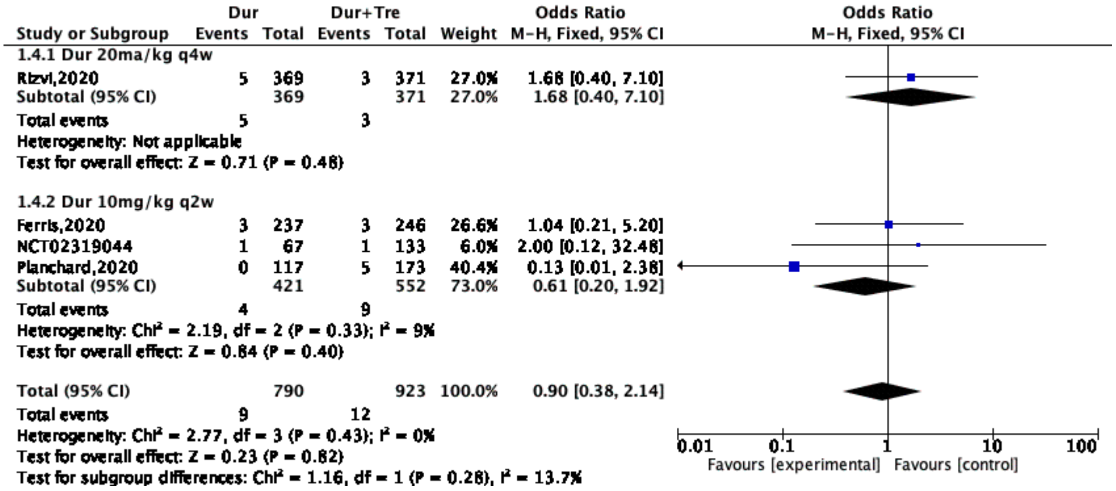

Study or Subgroup	Dur Events	Dur Total	Placebo Events	Placebo Total	Weight	Odds Ratio M-H, Random, 95% CI
1.5.1 Dur 750mg q2w						
Loibl,2019	0	92	0	82		Not estimable
Subtotal (95% CI)		92		82		Not estimable
Total events	0		0			
Heterogeneity: Not applicable						
Test for overall effect: Not applicable						
1.5.2 Dur 1500mg q4w						
NCT02551159	2	202	2	196	100.0%	0.97 [0.14, 6.95]
Subtotal (95% CI)		202		196	100.0%	0.97 [0.14, 6.95]
Total events	2		2			
Heterogeneity: Not applicable						
Test for overall effect: Z = 0.03 (P = 0.98)						
Total (95% CI)		294		278	100.0%	0.97 [0.14, 6.95]
Total events	2		2			
Heterogeneity: Not applicable						
Test for overall effect: Z = 0.03 (P = 0.98)						
Test for subgroup differences: Not applicable						

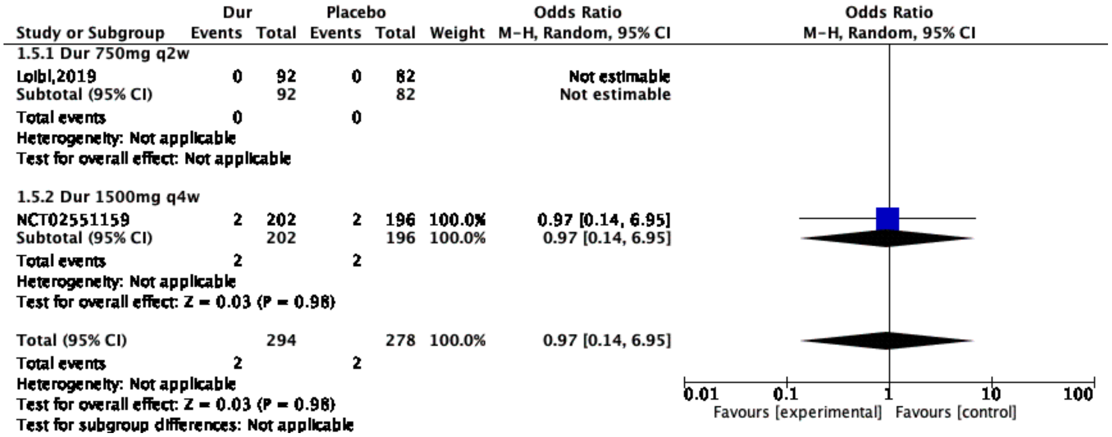

Study or Subgroup	Pem Events	Pem Total	Ipi Events	Ipi Total	Weight	Odds Ratio M-H, Fixed, 95% CI
1.6.1 Pem 10mg/kg q2w						
Robert2,2015	0	278	1	256	60.1%	0.31 [0.01, 7.54]
Subtotal (95% CI)		278		256	60.1%	0.31 [0.01, 7.54]
Total events	0		1			
Heterogeneity: Not applicable						
Test for overall effect: Z = 0.72 (P = 0.47)						
1.6.2 Pem 10mg/kg q3w						
Robert1,2015	1	277	1	256	39.9%	0.92 [0.06, 14.85]
Subtotal (95% CI)		277		256	39.9%	0.92 [0.06, 14.85]
Total events	1		1			
Heterogeneity: Not applicable						
Test for overall effect: Z = 0.06 (P = 0.96)						
Total (95% CI)		555		512	100.0%	0.55 [0.07, 4.20]
Total events	1		2			
Heterogeneity: Chi² = 0.26, df = 1 (P = 0.61); I² = 0%						
Test for overall effect: Z = 0.57 (P = 0.57)						
Test for subgroup differences: Chi² = 0.26, df = 1 (P = 0.61), I² = 0%						

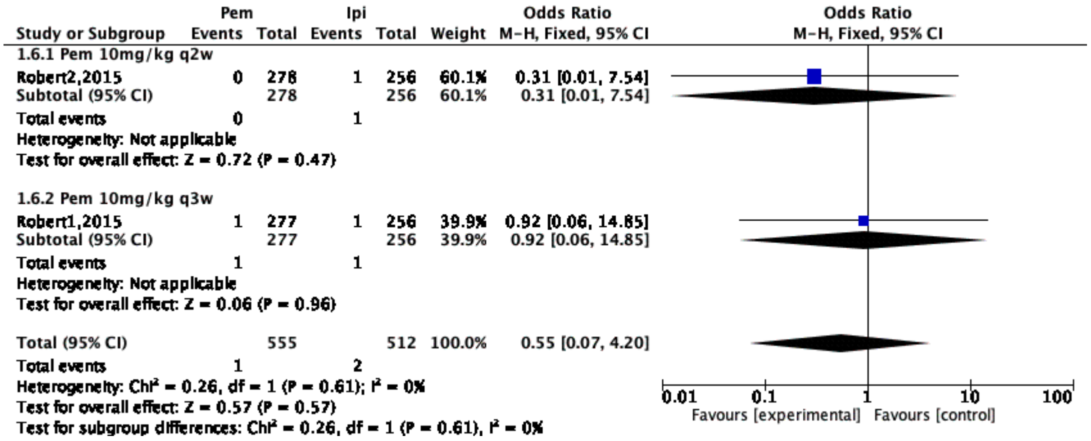

Study or Subgroup	Pem Events	Pem Total	Chemo Events	Chemo Total	Weight	Odds Ratio M-H, Fixed, 95% CI
1.7.1 Pem 2mg/kg q3w						
NCT01905657	15	339	2	310	49.4%	7.13 [1.62, 31.43]
Subtotal (95% CI)		339		310	49.4%	7.13 [1.62, 31.43]
Total events	15		2			
Heterogeneity: Not applicable						
Test for overall effect: Z = 2.59 (P = 0.009)						
1.7.2 Pem 10mg/kg q3w						
NCT01905657	10	344	2	310	50.6%	4.61 [1.00, 21.21]
Subtotal (95% CI)		344		310	50.6%	4.61 [1.00, 21.21]
Total events	10		2			
Heterogeneity: Not applicable						
Test for overall effect: Z = 1.96 (P = 0.05)						
Total (95% CI)		683		620	100.0%	5.86 [2.03, 16.91]
Total events	25		4			
Heterogeneity: Chi² = 0.16, df = 1 (P = 0.69); I² = 0%						
Test for overall effect: Z = 3.27 (P = 0.001)						
Test for subgroup differences: Chi² = 0.16, df = 1 (P = 0.69), I² = 0%						

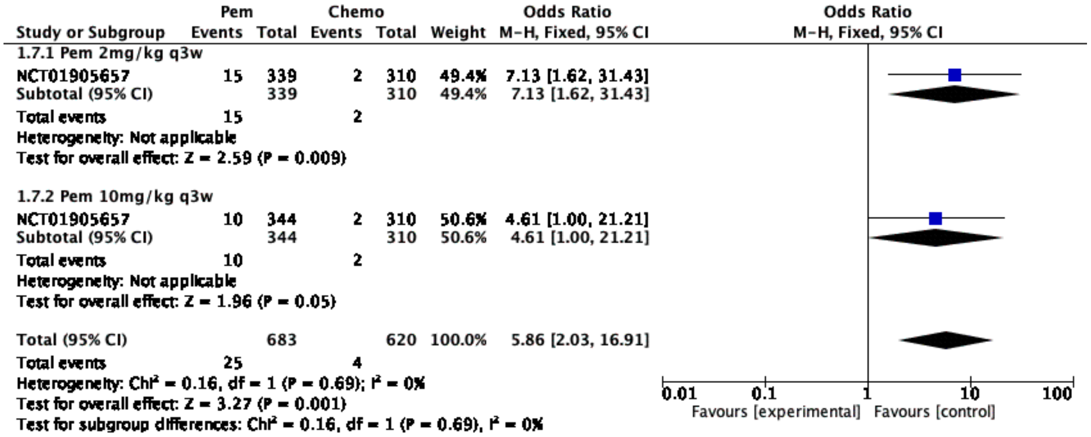

Study or Subgroup	Ipi Events	Ipi Total	PD1 Events	PD1 Total	Weight	Odds Ratio M-H, Fixed, 95% CI
1.8.1 Ipi 10mg/kg q3w						
Ascierto,2020	0	453	1	452	30.5%	0.33 [0.01, 8.17]
Subtotal (95% CI)		453		452	30.5%	0.33 [0.01, 8.17]
Total events	0		1			
Heterogeneity: Not applicable						
Test for overall effect: Z = 0.67 (P = 0.50)						
1.8.2 Ipi 3mg/kg q3w						
Hodi,2018	2	311	2	313	40.3%	1.01 [0.14, 7.19]
Robert1,2015	1	256	0	277	9.7%	3.26 [0.13, 80.34]
Robert2,2015	1	256	1	278	19.4%	1.09 [0.07, 17.46]
Subtotal (95% CI)		823		868	69.5%	1.34 [0.33, 5.44]
Total events	4		3			
Heterogeneity: Chi² = 0.40, df = 2 (P = 0.82); I² = 0%						
Test for overall effect: Z = 0.41 (P = 0.68)						
Total (95% CI)		1276		1320	100.0%	1.03 [0.30, 3.58]
Total events	4		4			
Heterogeneity: Chi² = 0.98, df = 3 (P = 0.81); I² = 0%						
Test for overall effect: Z = 0.05 (P = 0.96)						
Test for subgroup differences: Chi² = 0.62, df = 1 (P = 0.43), I² = 0%						

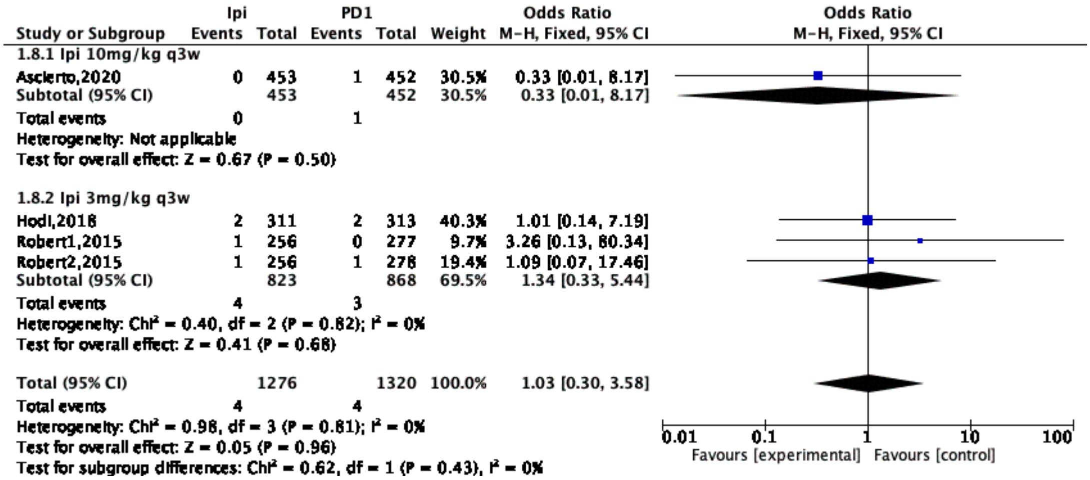

Study or Subgroup	Niv+Ipi Events	Niv+Ipi Total	Niv Events	Niv Total	Weight	Odds Ratio M-H, Fixed, 95% CI
1.9.1 Niv 1mg/kg q3w+Ipi3 mg/kg q3w						
Hodi,2018	8	313	2	313	29.1%	4.08 [0.86, 19.36]
Subtotal (95% CI)		313		313	29.1%	4.08 [0.86, 19.36]
Total events	8		2			
Heterogeneity: Not applicable						
Test for overall effect: Z = 1.77 (P = 0.08)						
1.9.2 Niv 3mg/kg q2w+Ipi 1mg/kg q6w						
Gettinger,2021	7	124	5	123	70.9%	1.41 [0.44, 4.58]
Subtotal (95% CI)		124		123	70.9%	1.41 [0.44, 4.58]
Total events	7		5			
Heterogeneity: Not applicable						
Test for overall effect: Z = 0.58 (P = 0.57)						
Total (95% CI)		437		436	100.0%	2.19 [0.88, 5.45]
Total events	15		7			
Heterogeneity: Chi² = 1.15, df = 1 (P = 0.28); I² = 13%						
Test for overall effect: Z = 1.68 (P = 0.09)						
Test for subgroup differences: Chi² = 1.14, df = 1 (P = 0.29), I² = 11.9%						

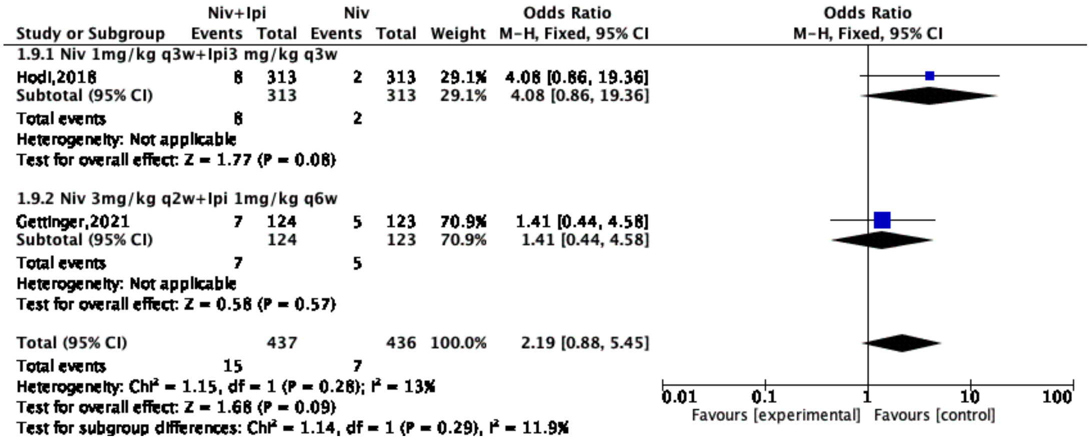

Study or Subgroup	Niv+Ipi Events	Niv+Ipi Total	Ipi Events	Ipi Total	Weight	Odds Ratio M-H, Fixed, 95% CI
1.10.1 Niv 1mg/kg+Ipi3 mg/kg						
Hodi,2018	8	313	2	313	75.2%	4.08 [0.86, 19.36]
NCT03033576	0	68	0	23		Not estimable
Subtotal (95% CI)		381		336	75.2%	4.08 [0.86, 19.36]
Total events	8		2			
Heterogeneity: Not applicable						
Test for overall effect: Z = 1.77 (P = 0.08)						
1.10.2 Niv 3mg/kg+Ipi 1mg/kg						
NCT02985957	1	73	0	38	24.8%	1.59 [0.06, 40.05]
Subtotal (95% CI)		73		38	24.8%	1.59 [0.06, 40.05]
Total events	1		0			
Heterogeneity: Not applicable						
Test for overall effect: Z = 0.28 (P = 0.78)						
Total (95% CI)		454		374	100.0%	3.46 [0.86, 13.99]
Total events	9		2			
Heterogeneity: Chi² = 0.27, df = 1 (P = 0.61); I² = 0%						
Test for overall effect: Z = 1.74 (P = 0.08)						
Test for subgroup differences: Chi² = 0.26, df = 1 (P = 0.61), I² = 0%						

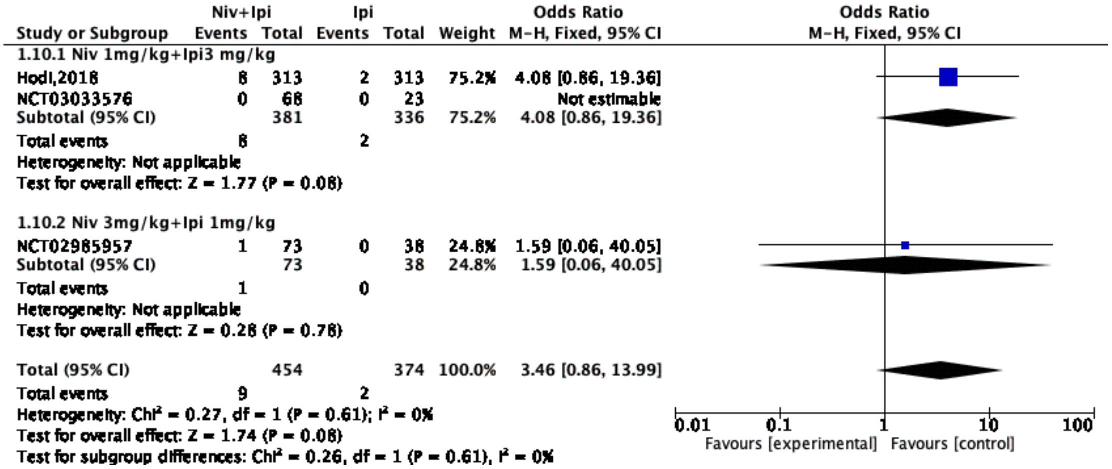

Abbreviations:PAEs, pulmonary adverse events;Chemo, Chemotherapy; Niv, Nivolumab; Ipi, Ipilimumab;Chemo, Chemotherapy;Ate, Atezolizumab.
